# A systematic methodology to assess the identity of plants in historical texts: A case study based on the Byzantine pharmacy text John the Physician’s *Therapeutics*

**DOI:** 10.1016/j.jep.2023.117622

**Published:** 2023-12-19

**Authors:** Andreas Lardos, Kristina Patmore, Robert Allkin, Rebecca Lazarou, Mark Nesbitt, Andrew C. Scott, Barbara Zipser

**Affiliations:** a ZHAW Zurich University of Applied Sciences, School of Life Sciences and Facility Management, Institute of Chemistry and Biotechnology, Natural Product Chemistry and Phytopharmacy Group, Einsiedlerstrasse 31, 8820, Wädenswil, Switzerland; b Royal Botanic Gardens, Kew, Richmond, London, TW9 3AE, UK; c Royal Holloway, University of London, Department of Earth Sciences, Egham, Surrey, TW20 0EX, UK; d Royal Holloway, University of London, Department of History, Egham, Surrey, TW20 0EX, UK

**Keywords:** Materia medica, Herbals, Dioscorides, Mediterranean

## Abstract

**Ethnopharmacological relevance:**

In recent decades, the study of historical texts has attracted research interest, particularly in ethnopharmacology. All studies of the *materia medica* cited in ancient and medieval texts share a concern, however, as to the reliability of modern identifications of these substances. Previous studies of European or Mediterranean texts relied mostly on authoritative dictionaries or glossaries providing botanical identities for the historical plant names in question. Several identities they suggest, however, are questionable and real possibility of error exists.

**Aim of the study:**

This study aims to develop and document a novel and interdisciplinary methodology providing more objective assessment of the identity of the plants (and minerals) described in these resources.

**Materials and methods:**

We developed an iterative experimental approach, using the 13th century Byzantine recipe text John the Physician’s *Therapeutics* in its Commentary version (JC) as a case study. The methodology has six stages and relies on comparative analyses including statistical evaluation of botanical descriptions and information about medicinal uses drawn from both historical and modern sources. Stages 1–4 create the dataset, stage 5 derives the primary outcomes to be reviewed by experts in stage 6.

**Results:**

Using Disocorides’ *De Materia Medica* (DMM) (1st century CE) as the culturally related reference text for the botanical descriptions of the plants cited in JC, allowed us to link the 194 plants used medicinally in JC with 252 plants cited in DMM. Our test sample for subsequent analyses consisted of the 50 JC plant names (corresponding to 61 DMM plants) for which DMM holds rich morphological information, and the 130 candidate species which have been suggested in the literature as potential botanical identities of those 50 JC plant names. Statistical evaluation of the comparative analyses revealed that in the majority of the cases, our method detected the candidate species having a higher likelihood of being the correct attribution from among the pool of suggested candidates. Final assessment and revision provided a list of the challenges associated with applying our methodology more widely and recommendations on how to address these issues.

**Conclusions:**

We offer this multidisciplinary approach to more evidence-based assessment of the identity of plants in historical texts providing a measure of confidence for each suggested identity. Despite the experimental nature of our methodology and its limitations, its application allowed us to draw conclusions about the validity of suggested candidate plants as well as to distinguish between alternative candidates of the same historical plant name. Fully documenting the methodology facilitates its application to historical texts of any kind of cultural or linguistic background.

## 1 Introduction

This study is part of the project “Plants and minerals in Byzantine popular pharmacy. A new multidisciplinary approach” which is focused on the development of transferrable methodologies for the analysis of *materia medica* in premodern texts.

### 1.1 Potential and problems of historical texts in ethnopharmacology

Many cultures have documented their knowledge of the medicinal use of plants, minerals and other natural products in written form. Since cultures evolve or disappear, these texts offer a unique gateway to knowledge which would otherwise be lost. In recent decades, the study of historical sources has attracted research interest, particularly in ethnopharmacology. Studies have undertaken to improve our understanding of how medicinal knowledge evolved, or to re-utilise this information, for example to develop new medicines based on historical uses of plants (Lardos, 2015). A prominent example illustrating such potential was the development of artemisinin from *Artemisia annua* L. (Asteraceae) as a medication for malaria, based on a 4th century CE text of Chinese medicine. Remarkably, the plant’s active principle could only be successfully isolated by adhering strictly to the extraction protocol suggested in the historical text (Hsu, 2006; Tu, 2016). Today, combination therapies of artemisinin or its derivatives constitute an essential therapeutic option when treating malaria (WHO, 2022). Historical studies with a pharmacological focus have also been conducted, using texts from both European and Mediterranean traditions, for example, Renaissance herbals from Germany and Italy (Adams et al., 2009, 2011a), post-Byzantine *iatrosophia* texts from Cyprus (Lardos et al., 2011), Anglo-Saxon sources (Harrison and Connelly, 2019), or a 14th century Welsh medical manuscript (Wagner et al., 2017). Some plants identified through the study of these texts have also been tested and found to exhibit pharmacological activities corroborating their historical uses (Adams et al., 2011b; Zimmermann et al., 2012; Wagner et al., 2017).

The investigation of historical texts offers numerous challenges which vary considerably depending on a text’s language, date or cultural background (Riddle, 2007). Linguistic and philological barriers, retrospective diagnosis and the laborious process of making the content accessible to modern analytical tools, are common. An issue of particular difficulty and complexity in the ethnopharmacological investigation of these resources is the identification of the plants or plant substances mentioned in the texts (Riddle, 1996; Lev and Amar, 2006; Lardos et al., 2011). Most previous studies on European or Mediterranean texts relied on authoritative dictionaries or glossaries which provide botanical identities for the historical plant names in question. Cross-referencing a name mentioned in the text with names in such references suggests the putative identity of the herbal *materia medica* concerned. However, there are various problems and uncertainties: i) different authorities often disagree how plant names from, for example, Greek or Roman antiquity should be interpreted. Some of the suggested identities are questionable and potentially erroneous, as pointed out by Riddle (1996) or Raven et al. (2000) and as illustrated in detail by Evergetis and Haroutounian (2015); ii) botanical names provided in those references are themselves often ambiguous or imprecise due to the use of synonyms, illegitimate or invalid names, and through lacking complete author citations. The use of inappropriate or erroneous scientific nomenclature may compromise the reliability or value of the published work (Rivera et al., 2014) and prevent comprehensive access to relevant past research (Allkin and Patmore, 2022); iii) uncritical adoption of previously suggested identities can be misleading. Various studies indicate that consideration of the specific phytogeographical, cultural-historical and philological aspects of a given text is indispensable for drawing reliable conclusions (see e.g. Riddle, 1996; Lev and Amar, 2006, Lardos et al., 2011; Touwaide and Appetiti, 2013).

In their study of the Umbelliferae (Apiaceae) mentioned in the *Codex Neapolitanus Graecus 1*, a 7th century CE recension of Dioscorides’ treatise *De materia medica* (*Peri ylēs iatrikēs*, 1st century CE), Evergetis and Haroutounian (2015) demonstrated one possibility for circumventing uncertainties regarding previously suggested botanical identities. They used images and text from the historical source to construct plant descriptions and compare with information available in modern floristic works. In doing so they were able to establish the botanical identities of the respective Dioscoridean plants and re-assess previous identifications. Clearly their approach was tailored to texts with a pharmacognostic emphasis, such as the illustrated editions of Dioscorides’ *De materia medica*, containing images and detailed descriptions of the plants.

However, a class of historical texts which generally lacks plant descriptions or illustrations, but which is of particular interest to pharmacological investigation, is the class of recipe texts found in the medical traditions of various cultures. Only a few of these have so far been the subject of systematic ethnopharmacological investigation, e.g. the Taylor-Schechter Genizah Collection from the Jewish community of medieval Cairo (Lev and Amar, 2006), post-Byzantine *iatrosophia* texts from Ottoman Cyprus (Lardos and Heinrich, 2013), the herbal medicines in Nikolaos Myrepsos’ *Dynameron* from the late Byzantine era (Valiakos et al., 2015, 2017), or *The Physicians of Myddfai*, a Welsh medieval manuscript (Wagner et al., 2017). The problem with these texts is that the only information given for the plants mentioned is their name in the local vernacular from that place, time and culture.

How might we, therefore, identify plants cited in these kinds of texts with a therapeutic emphasis, but which lack illustrations or descriptions of plants? An integrated approach becomes necessary, considering botanical features (plant morphology, ecology and geography) alongside archaeobotanical, ethnopharmacological, cultural-historical and philological aspects.

### 1.2 The case-study: a 13th century byzantine text

In order to address this problem, we took the position that an interdisciplinary approach was essential, assembling a multidisciplinary team from philology, history, bioinformatics, archaeology, geology, botany, palaeobotany and ethnopharmacology. The recipe text selected by the team to develop and trial its methodology is a medieval pharmaceutical text from the Byzantine tradition: The *Therapeutics* of John the Physician (Ioannes Arkhiatros in Greek). This recipe text has a long history, ultimately going back to Paul of Aegina from the 7th century Byzantine Empire (Zipser et al., 2020). We are uncertain of its early development until we reach the original version of John the Physician’s *Therapeutics*, a concise if occasionally disorganised therapeutic handbook (Zipser, 2009). It is written in a simplified version of Classical Greek that was commonly used in writing in the Middle Ages. The work was then translated into the vernacular in which a medical practitioner or a person of similar knowledge explained how to prepare each medication. This version of the text is also one of the earliest longer testimonies of vernacular Greek, an idiom that was used in everyday discourse but rarely in writing. Several indicators point to Cyprus as its origin and allow us to date it to the late 13th or early 14th century (Zipser, 2009). This vernacular version of the text with commentary, specified as version ω in Zipser (2009), forms the basis for the present paper and is referred to here as John’s Commentary (JC).

Collections of Byzantine recipes vary in genre, covering content from therapy to pharmacy and cosmetics, and from scholarly texts to household lists. These recipes played an important role in Byzantine medicine and can be considered to distil local traditional knowledge (Stannard, 1984). They reflect the ability of Byzantine medicine to compile and hand down the best and most useful treatments (Bennett, 2000). The recipes in these collections are often straightforward statements of the ailment to be treated, the ingredients needed, their preparation, and how to apply them.

Typically, the recipes’ ingredients are specified only by their name without any further description. This leads to two important questions: How did people know what plant, mineral or animal part a given name referred to? And how did they know where that material could be sourced? Even if we presuppose that anyone who dealt with these texts, whether author or user, had the necessary botanical or pharmacognostic knowledge, there must have existed reference texts to consult for confirmation and to provide additional details where there was uncertainty.

Dioscorides’ *De materia medica* (DMM) had this role in the Byzantine line of tradition. His treatise is one of the most influential historical pharmacy texts, quoted for more than 1600 years by authors from Europe to the Middle East (Riddle, 1985). Recent findings suggest a substantial influence of Dioscorides’ *De materia medica* on the development of both the *materia medica* in European historical texts (De Vos, 2010), and orally transmitted popular medicine in the Mediterranean (Leonti et al. 2009, 2010). Throughout the Byzantine era copies of DMM were produced, not primarily as a means of preserving a tradition but rather for practical use and consultation (Riddle, 1984). Practically all Byzantine pharmaceutical texts show an influence from DMM in terms of the drugs used (Stannard, 1984), including recipe texts such as the *iatrosophia* (Touwaide, 2007) and in these texts as late as the 19th century (Lardos, 2006). As the general reference for *materia medica*, DMM also serves as a guide to drug descriptions and nomenclature. We therefore conclude that the identity of plants or minerals mentioned in Byzantine recipes can only be elucidated by considering the descriptions of these materials in culturally and geographically associated pharmacognostic texts, in this case DMM. Besides its cultural-historical links, DMM is particularly appropriate for our study because of its biogeographical setting. Dioscorides came from Anazarbos, a historical town in the southeast of today’s Turkey. Places of origin for plants in his treatise were most frequently cited as locations in Anatolia and the Eastern Mediterranean region (Riddle, 1985). This, in addition to the presumed Cyprus origin of JC (Zipser, 2009), suggests a likely strong connection between the herbal *materia medica* mentioned and the flora of these adjoining regions.

### 1.3 Research questions

The present study addresses an underlying concern regarding the reliability of published identifications of the plants and minerals cited in ancient and medieval texts. Our primary research question is to what extent it is possible to identify the plants (or minerals) from a particular source by comparing the available historical data with modern descriptions of the possible candidate plants: their morphology and uses. Is it possible to analyse that data and apply botanical and pharmacological expertise to differentiate between the alternative candidate plants and to objectively assign relative likelihood of each being the correct attribution?

The aim of the study therefore was the iterative development of an interdisciplinary methodology providing workflows and data procedures for future use by other researchers. We have sought to make the methodology and the data we use transparent so that other researchers can re-use or critique our work. Our wider goal is for a methodology transferable to analysis of other texts independent of their cultural or linguistic setting. For the development and testing of our methodology we used John’s Commentary (JC) for the plant and mineral names and their uses, and consider Dioscorides’ *De materia medica* (DMM) as the associated pharmacognostic reference.

This paper focuses on the plants in JC. Scott et al. (2022) and further papers in preparation describe our research and treatment of minerals and burnt substances in JC.

## 2 Material and methods

### 2.1 Primary sources

John’s Commentary (JC) is the recipe text used as the reference for both plant and mineral names and their uses. The edition is based on the Leithandschrift L and a group of other manuscripts of slightly lower quality (Zipser, 2009).

Dioscorides’ *De materia medica* (DMM) is the pharmacognostic reference text used for the descriptions of the plant names mentioned in JC. Botanical information about the plants from DMM was compiled from Beck’s (2005) English translation. The Greek plant names were adopted from Wellmann’s (1907) edition. Crucial passages were consulted in the Greek original as presented in Wellmann’s edition.

### 2.2 Development of the methodology

Our novel approach to determine the identity of plants is based on comparative analyses of botanical descriptions and information about their medicinal uses drawn from both historical and modern sources. We followed an iterative approach: repeated cycles of design, test, evaluate and adapt. We processed plants in batches as we enhanced our methodology. We followed the Consensus Statement for Ethnopharmacological Field Studies (ConSEFS) best practice guidelines with special consideration of points relating to historical studies (Heinrich et al., 2018). The final version of the methodology (version 12) was applied to all plants and consisted of six stages, each containing one or more individual steps (Supplementary material, Figure S1). These six stages were.

1) Gathering data from the historical texts
2) Establishing the list of suggested candidate plants
3) Gathering data from modern sources
4) Building the data matrices and comparative analyses
5) Statistical evaluation
6) Reflection and review

**Fig. 1:**
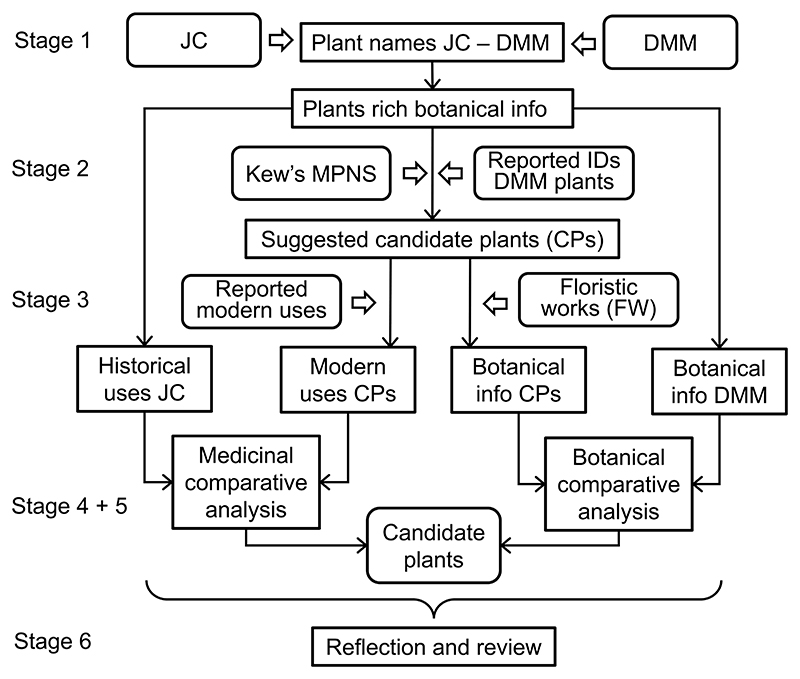
Flowchart illustrating the six stages of the methodology: Stage 1 – Gathering data from historical texts; Stage 2 – Establishing the list of suggested candidate plants; Stage 3 – Gathering data from modern sources; Stage 4 – Building the data matrices and comparative analyses; Stage 5 – Statistical evaluation; Stage 6 – Reflection and review of the methodology. The figure shows the outputs of each stage of the methodology as well as the resources used (Abbreviations: JC, John the Physician’s Commentary; DMM, Dioscorides’ *De Materia Medica*; Kew’s MPNS, Kew’s Medicinal Plant Names Services; FW, Floristic works).

Stages 1–4 are concerned with creating the dataset upon which our conclusions are based, Stage 5 derives our primary outcomes, which were then reviewed by experts in Stage 6 (Fig. 1).

#### 2.2.1 Gathering data from the historical texts (stage 1)

##### 2.2.1.1 Step 1.1 – word tagging and translation of JC

A concordance of the JC text was built using AntConc, a freeware corpus toolkit for text analysis (Anthony, 2022). Every recipe contained in JC was assigned a unique signature consisting of the chapter number followed by the recipe number (e.g. 178.03). A list of all words contained in the recipes was then produced using the word list feature in AntConc (Anthony, 2022). Every word was tagged manually, assigning it an identifier and categorising it into one of the following six categories: “Plant (JCP)”, “Mineral (JCM)”, “Animal (JCA)”, “Multiple (JCX, including compound preparations of plant, mineral or animal origin, and ambiguous materials)”, “Other (JCO, for words that were neither plants, minerals nor animals)”, “Unknown (JCU)”. Each word tag is composed of its word category and a unique four digit number (e.g. JCP_2853).

To categorise the *materia medica* the respective elements were assessed from scratch. First, words that could be clearly identified from descriptions in other primary sources (e.g. words referring to water, honey or olive oil), as well as words unambiguously referring to animal and mineral products were placed in their respective categories. Words clearly referring to plants because they appeared together with plant parts (e.g. leaves of, flowers of), were moved to the category JCP. Finally, every remaining word of the *materia medica* was assessed individually by running a query in the full corpus of Thesaurus Linguae Graecae® (TLG, n.d.). Once this philological work was completed, the categories were checked from a modern (Cypriot) Greek and pharmacognostic perspective.

A complete English translation of the recipes in JC was produced. For the further investigation of the recipes, this translation was used alongside the original Greek text.

##### 2.2.1.2 Step 1.2 – lemmatising names of plants and minerals in JC

To analyse which plants and minerals were mentioned in JC all tagged plant names (and mineral names) were lemmatised. Words with the same root were grouped together so that all inflected forms of a name and its spelling variants or combinations were given the same lemma tag (the respective code and three digit number). The lemma tag JCLP_197, for example, included all words linked with the root ^*^σκóρδ (e.g., σκóρδα, σκóρδον, σκóροδα, σκóροδον, σκορóδου) and were given the lemma (dictionary form) “σκóρδον”. Compound words such as πηγανέλαιον (JCLP_157), referring to ἔλαιον (oil) of the plant πήγανον (JCLP_157), were listed under the respective plant name. In general, a common sense approach seemed to be the best way forward, in combination with individual checks of any such compound words throughout the process.

##### 2.2.1.3 Step 1.3 – cross-referencing of JC plant names to DMM plant names

Taking Dioscorides’ *De materia medica* (DMM) as the primary pharmacognostic reference for the plants cited in JC, each lemmatised JC plant name was cross-referenced to the corresponding entry in DMM according to Wellmann (1907). The connections between each JC name and a DMM name was classified as belonging to one of the following categories: 1) Same: the JC name being identical to the DMM name; 2) Synonym: the JC name is considered a synonym of the DMM name; 3) Source plant: the JC name refers to a part of or product made from the DMM plant; 4) Unclear: the link between the JC and DMM names is unclear. 5) No connection: the JC name has no connection with any DMM names. These links were assessed using dictionaries and glossaries of Greek plant names (Langkavel, 1866; Gennadios, 1914; Sophocles, 1914; LSJ, 2022).

##### 2.2.1.4 Step 1.4 – capturing botanical information from DMM

For each JC plant name connected to a DMM entry, we compiled the botanical information available in DMM from Beck (2005), consulting Wellmann’s (1907) edition in the original language where necessary. The data compiled were assigned to data categories across three themes.

i) Morphological information: including various categories relating to the life form, habit, height, stem, leaf, flower, fruit, seed, root;
ii) Ecological information: categories relating to the habitat of the plant;
iii) Geographical information: categories relating to the distribution of the plant or its place of origin (in the case of traded substances).

Having substantial morphological information available was considered absolutely key. We therefore classified each JC plant name into one of three categories, depending on the extent and level of detail of the morphological information available in DMM.

1) Rich: morphological information on several plant parts including some specific details;
2) Moderate: morphological information on a few plant parts, though mostly lacking details;
3) Poor: morphological information is lacking or imprecise and unspecific.

Further analyses of the herbal *materia medica* could only be conducted for JC plant names for which we had rich botanical information available in DMM. For these plants we collated additional information from DMM relating to their smell and taste, or ethnobotanical information concerning any other (non-medicinal) uses. Any botanical information available in JC was also captured in the same spreadsheet.

##### 2.2.1.5 Step 1.5 – capturing medicinal plant uses from JC

For each JC plant name connected to a DMM entry with rich botanical information we extracted the use records from JC. We defined a use record as being a reference within JC to a specific herbal substance having a particular medicinal use. For each use record, we compiled: the plant name, the plant part used, the medicinal use. Each use record was assigned to one of 21 medicinal use groups representing human body parts, systems or pathologies: AN – Andrology, BS – Blood, spleen, CV – Cardiovascular, DE – Dermatology, FV – Fevers, GI – Gastrointestinal tract, GY – Gynaecology, ID – Infectious diseases, LG – Liver and gall-bladder, MA – Maternity, MC – Mental conditions, MN – Metabolic and nutritional disorders, MS – Musculo-skeletal, NC – Neurological conditions, OP – Ophthalmology, OC – Oral cavity, OT – Otology, RE – Respiratory tract, RL – Rhino-Laryngology, UR – Urology, XY – Residual category. This classification system follows the recommendations of Staub et al. (2015) for cross-cultural comparisons in ethnopharmacological studies (Model 6.II in Staub et al., 2015) and is largely based on the WHO’s International Classification of Primary Care (ICPC) (https://www.who.int).

##### 2.2.1.6 Step 1.6 – collating burnt substances from JC

Burnt substances of plant, mineral or animal origin that are cited as ingredients in JC recipes were also extracted from the text. Samples of these findings will be discussed in other publications (Scott et al., 2022, and further paper in preparation).

Therefore, the subsequent stages 3–6 of the methodology were followed only for the plants cited in JC for which rich botanical information was available in DMM.

#### 2.2.2 Establishing the list of suggested candidate plants (stage 2)

##### 2.2.2.1 Step 2.1 – compiling identities of plants reported in the literature

For each JC plant name in our sample, we compiled a list of the possible botanical identities previously suggested in the literature. Information was drawn from.

i) Earlier scholars who studied the flora of Greece and the Eastern Mediterranean and suggested identifications for plants mentioned in DMM (Sibthorp, eds. Smith and Lindley, 1806–1840; Billerbeck, 1824; Fraas, 1845; Lenz, 1859);
ii) Modern ethnobotanical field studies from Greece and Cyprus reporting Greek plant names (Arnold-Apostolides, 1985; Hanlidou et al., 2004; Della et al., 2006; Karousou and Deirmentzoglou, 2011; Lardos, 2016; Axiotis et al., 2018; Tsioutsiou et al., 2019).

Any botanical taxon suggested in the literature was included as a candidate if the respective Greek plant name corresponded to the plant name appearing in our sample.

##### 2.2.2.2 Step 2.2 – cross-checking botanical names with MPNS

We employed Kew’s Medicinal Plant Names Services (MPNS, 2023) to validate and harmonise the scientific nomenclature and taxonomy of all reported candidate species names. MPNS derives its scientific nomenclature and taxonomy from the *World Checklist of Vascular Plants* (Govaerts, 2017). MPNS thus provided the currently accepted scientific name of each candidate and a complete list of scientific synonyms. Where multiple possible matches were found, an expert familiar with the regional flora manually selected the most appropriate choice(s) in each case. Validation via MPNS served to resolve spelling mistakes or inconsistencies in the names as published in the literature and ensured scientific integrity. This avoided a single plant being listed multiple times under alternative synonyms (Allkin and Patmore, 2022).

##### 2.2.2.3 Step 2.3 - preparation of the list of suggested candidate plants

A de-duplicated list was prepared of all suggested candidate plants for each JC plant name in our sample, using their accepted scientific names as seen at point of MPNS validation. This served as the basis for stages 3, 4, and 5 of this study.

#### 2.2.3 Gathering data from modern sources (stage 3)

We gathered botanical and medicinal information from modern publications for each of the suggested candidate plants.

##### 2.2.3.1 Step 3.1 – collating botanical information from floristic works

The medicinal flora of DMM is strongly linked to the flora of Anatolia and the Eastern Mediterranean. JC is presumed to have come from Cyprus (see Introduction). We therefore used floristic works of these regions. The *Flora of Turkey and the Eastern Aegean Islands* (FT) by Davis et al. (1966–85, suppl. 1988 and 2001) was our primary reference. The *Flora of Cyprus* (FC) by Meikle (1977, 1985) was used to complement FT and for plants not contained in it. To ensure that we reliably searched the index of each botanical source for candidate plants, we employed the complete list of synonyms from Kew’s MPNS (2023). Using the above sources, we compiled botanical descriptive information on each of the candidate plants and, where necessary, on its variety, subspecies or genus. To permit a direct comparison, data were extracted using the botanical criteria which had been used to extract information from DMM.

##### 2.2.3.2 Step 3.2 – collating medicinal uses from herbal medicine and ethnobotany

Information on modern plant uses of each candidate plant was compiled from: i) Standard texts of herbal medicine: *British Herbal Pharmacopoeia 1983*; BHMA, 1983), *British Herbal Pharmacopoeia 1996*; BHMA, 1996), *Potter’s Cyclopaedia of Botanical Drugs and Preparations* (Wren, 1975), *Potter’s Herbal Cyclopaedia* (Williamson, 2003), *Principles and Practice of Phytotherapy* (Bone and Mills, 2013); ii) Ethnobotanical field studies conducted in the Near East, Turkey, Greece and Cyprus (Arnold-Apostolides, 1985; Karousou and Deirmentzoglou, 2011; Rivera et al., 2012; Lardos and Heinrich, 2013). The medicinal plant uses reported were then categorised into the same medicinal use classification as applied to plant uses in JC (see step 1.5).

##### 2.2.3.3 Step 3.3 – compiling medicinal uses from human clinical trials

For each candidate plant, information on human clinical trials was compiled from PubMed Central® (PMC, 2022). To achieve comprehensive retrieval of publications it was necessary to employ all known scientific synonyms for each plant. Kew’s MPNS portal (2023) offers this functionality and was used to carry out comprehensive searches. The remedies indicated by published studies were categorised using the same medicinal use classification (see step 1.5).

#### 2.2.4 Building the data matrices and comparative analyses (stage 4)

Comparative analyses of the historical and modern data required compatible data matrices of both the botanical and the medicinal information.

##### 2.2.4.1 Step 4.1 – botanical comparative analysis

To facilitate comparison of historical and modern descriptions of the plants involved, a data matrix was built containing comparable data categories drawn from DMM (step 1.4) and from modern floristic works for each candidate plant (see Step 3.1) (see Supplementary material, Table S2 for an illustration of the botanical analysis using the example of the JC plant name alyssos (ἄλυσσος)).

For each JC plant name in our sample, details of botanical features reported for the associated plant in DMM, were compared with corresponding details from the modern floristic works (FT, FC) for each candidate plant. Whenever possible the information was drawn from the species description in the floristic works, while the genus description was only consulted to complete missing details. Each plant detail was then assigned a “match value” depending upon the degree of congruence between the historical and modern observations. For the purposes of later analysis, each of these classes was then converted to a score. Where no correlation was seen between available descriptions, a negative score was applied:

Good match (score = 2): There is a good congruence between the description of the respective characteristic in DMM and the corresponding information in FT or FC;

Partial match (1): The information stated in the historical text and in the floristic works appear to describe the same characteristic, but there is some doubt, e.g. due to different terminologies or different perspectives;

No match (−2): No correlation was observed between the description of the respective characteristic in DMM and the corresponding information in FT or FC;

Absent (0): No information was found in FT and/or FC which corresponded to the respective characteristic described in DMM.

##### 2.2.4.2 Step 4.2 – medicinal comparative analysis

To facilitate comparison of historical uses with modern evidence, two data matrices were built to reflect uses drawn from herbal medicine and ethnobotanical sources (step 3.2), and from human clinical trials (step 3.3), for all candidate plants. Each used the same use group categories as applied to the historical uses drawn from JC (see step 1.5) and these were then combined into a single consolidated modern uses matrix in which, for each candidate plant, a True or False (“1” or “0”) value was assigned to each use group depending on whether any modern sources had reported such a category of use or not.

#### 2.2.5 Statistical evaluation (stage 5)

The comparative analyses conducted in stage 4 provided the data for the statistical evaluation of botanical and medicinal information from modern and historical sources.

##### 2.2.5.1 Step 5.1 – data processing of the botanical features matrix

In an intermediate step before the statistical analysis of the botanical features matrix (see step 4.1), high level feature categories were created to group associated botanical details. The available detail scores for each candidate plant were averaged within these feature categories. This was done to avoid undue emphasis on those features reported in greater detail, compared with those reported in less detail in DMM. For example, a total of up to seven details were reported for leaves (including size, shape, edge and surface), where only up to 3 details were reported for flowers (general, colour and detail). The final scored feature categories were as follows, with the total number of possible details included in each in parentheses: lifeform (1), habit (3), height (1), stem (4), leaf (7), flower (3), fruit (5), seed (3), root (4), properties (2), ethnobotany (1), habitat (1) and distribution (1).

From this consolidated matrix, a total score for positively corresponding feature categories and a total score for negatively corresponding feature categories was derived for each candidate plant. The negative score (S_*neg*_) was then subtracted from the positive score (S_*pos*_) to give a final score of degree of congruence between each JC plant name and the respective candidate plant (Degree of congruence = S_*pos*_ – S_*neg*_).

These final scores were used to establish a measure of candidate strength as follows:

Strong (score ≥5): The total number of congruent feature categories is significantly higher than the number of incongruent feature categories;

Moderate (3–4): The total number of congruent feature categories is nominally higher than the number of incongruent feature categories;

Weak (≤2): The total number of congruent feature categories was lower, or not substantially higher, than the number of incongruent feature categories;

Not Found (0): The corresponding candidate plant is not mentioned in the floristic works used.

Applying a measure of this type enables differentiation of candidate plants into broad groups of species that are more or less likely to be the plant intended in DMM or JC respectively (see Supplementary material, Table S3 for an illustration of the data processing using the example of the JC plant name *alyssos* (ἄλυσσος)). The score thresholds applied to do this were based on a holistic overview of the dataset outputs and can be easily adjusted to further analyse or refine results. More detailed manual analysis would likely be needed to truly differentiate between candidate plants within each group.

##### 2.2.5.2 Step 5.2 – data processing of the medicinal features matrix

The consolidated modern uses matrix (step 4.2) was combined with the historical uses from JC (step 1.5) to produce a final matrix in which, for each candidate plant, every use group was allocated one of four tags:

Historical & Modern: Both historical (JC) and modern sources report that this species has been used in this use category.

Historical Only: Only JC reports that this species has been used in this use category, no modern reports seen.

Modern Only: Only modern sources report that this species has been used in this use category, no reports seen in JC.

Absent: Neither historical (JC) nor modern sources report this species as having this category of use.

From this, basic scores were derived indicating the number of instances of correlating usage (“Historical & Modern” or “Absent”), and the number of instances of non-correlating usage (“Historical Only” or “Modern Only”). For the purposes of final analysis, only the percentages of JC-only uses (“Historical Only”) and shared uses (“Historical & Modern”) were considered meaningful and used to calculate a measure of candidate strength similar to that applied to the botanical analysis:

Strong (score = 3): More than 50% of total uses seen in JC are also reported in the modern sources.

Moderate (2): Between 20% and 50% of total uses seen in JC are also reported in the modern sources.

Weak (1): Less than 20% of total uses seen in JC are also reported in the modern sources.

No modern uses found (0): No modern uses were found across any use categories for this species.

As for the botanical analysis, the score thresholds can be adjusted to further analyse or refine results.

##### 2.2.5.3 Step 5.3 – statistical evaluation

As part of the data processing in steps 5.1 and 5.2, each candidate plant was allocated two measures of candidate strength.

Because of the transitory nature of plant use over regions, time and medical traditions, we considered the medicinal uses analysis a far less robust measure than the botanical descriptive information which is based on features that are more objectively observable and constant over time.

For this reason, in the final statistical evaluation, candidate plants were grouped alongside their respective JC plant names under the broad categories “strong”, “moderate” and “weak”, according to the results of the botanical analysis (step 5.1). These measures of candidate strength constitute the core degree of confidence we have determined that each candidate plant may be a match for its respective JC plant name.

To supplement this, for each candidate plant the result of the medicinal uses analysis (step 5.2) has also been included as a score in parentheses after the scientific name. This approach enables a simple overview of all candidate plants ascertained for each JC plant name, with priority candidates for further research easily identified, first by the strength of their botanical congruence, and then further differentiated by the medicinal uses score.

For example, looking at the JC plant name *alyssos* (ἄλυσσος; Lemma tag JCLP_013), stage 2 of the process identified three candidate plants from the literature (*Fibigia clypeata* (L.) Medik., *Odontarrhena alpestris* (L.) Ledeb. and *Scutellaria galericulata* L.). Of these, only *Fibigia clypeata* demonstrated strong botanical congruence with descriptions from DMM in the botanical analysis (Supplementary material, Table S3). However, we found no medicinal uses reported in the modern literature scrutinised. In contrast, only *Scutellaria galericulata* is known to have medicinal uses according to the modern literature, but this plant demonstrated weak botanical congruence with descriptions from DMM.

As part of developing this evaluation a number of other methods of analysis were also explored and some were used informally to assess our findings and guide development of the above scoring parameters (see **Research data, Data file 11**, https://figshare.com/s/2e0f64b5794892ed4679).

#### 2.2.6 Reflection and review (stage 6)

This stage involved an expert secondary review of the methodology, the outcomes, and reflection upon successes, challenges and lessons. For this purpose a two-day workshop was conducted at the Royal Botanic Gardens, Kew on March 14th to 15th, 2023 with participation of all contributing authors, our collaborator Efraim Lev (University of Haifa), and independent scholar Leigh Chipman.

## 3 Results

Textual analysis of JC allowed us to distinguish 1414 recipes citing 289 plant names and 43 mineral names. Each recipe provided information about the symptom or disease treated, the *materia medica* to be used, the method of preparation and how to apply the remedy. We found 26,967 words in recipes that could be tagged to the following categories: Plants (JCP) – 2558; Minerals (JCM) – 469; Animals (JCA) – 749; Multiple (JCX) – 176; Other (JCO) – 22,989; Unknown (JCU) – 26. A linguistic approach to lemmatisation together with a pharmacognostic cross-check enabled a rapid and unambiguous grouping of most words (**Research data, Data file 1**, https://figshare.com/s/2e0f64b5794892ed4679).

Burnt substances (from plants or other natural origins) play an important role in many JC recipes. In many cases it is not clear why this should be. To better understand the processes and uses of such materials as well as derive possible candidates, a series of experiments was undertaken. These results will be published elsewhere (Scott et al., 2022, Scott, in preparation).

The following sections focus on the plants mentioned in JC. They show the development of both the historical dataset (section 3.1) and modern dataset (3.2, stages 2, 3, 4) followed by comparative analyses of these datasets (3.3, stage 5) and the subsequent revision and reflection (3.4, stage 6).

### 3.1 Historical dataset

Among the 289 plant names mentioned in JC, we can distinguish names referring to whole plants (203 cases), products of plant origin (45), or plant parts (25) and plant substances with dedicated names (16). The most frequently mentioned plants or plant substances, by a priori assessment, are πήγανον – rue 44x, πέπερι – pepper 42x, ἀψíνθιον – wormwood 34x, λíβανος – olibanum 29x, and ἀμμωνιακóν – gum ammoniac 27x. While the most frequently mentioned products are κρασíν – wine 231x, ὄξος – vinegar 229x, οἶνον – wine (synonym) 99x, ἔλαιον – olive oil 72x, χρηστέλαιον – lower quality olive oil 57x, and ῥοδέλαιον – rose oil 54x (Supplementary material, Table S1).

Cross-referencing the 289 JC plant names with DMM reveals the close relationship of the two texts in terms of the nomenclature of herbal *materia medica*. The great majority of the plant names, 274 cases (95%), have a connection to plant names in DMM. These 274 JC plant names can be traced back to 194 source plant names. No fewer than 165 (85%) of these are addressed in JC with the same name as in DMM. For the remaining 29 (15%) plants, the name used in JC is considered a synonym to a plant name in DMM (Supplementary material, Table S1).

Because some plant names in JC are under-differentiated, meaning that one JC plant name can relate to one or more DMM plants, the 194 unique JC plant names are associated with 252 DMM plants. For example, the JC plant name ἀρνóγλωσσον (JCLP_023) refers to two types of the DMM plant ἀρνóγλωσσον – μικρóν and μεῖζον (DMM II, 126) (Supplementary material, Table S1).

Of the 252 plants with botanical information available in DMM, 61 (24%) hold rich morphological data, 74 (29%) moderate data and 117 (47%) poor information. Many of the plants with only poor information are generally identified as important crop plants (e.g. έλαíα – olive, σŨκα – figs), common aromatic herbs (e.g. ἄνησσον, ἡδÚοσμον), or exotic herbal drugs (e.g. ζιγγίβερι, λίβανος) (**Research data, Data file 2**, https://figshare.com/s/2e0f64b5794892ed4679). Interestingly, many of these plants still have the same name in Modern Greek.

The 61 plants with rich information in DMM are linked with 50 plant names in JC. We used these 50 JC plant names and the associated 61 information-rich DMM plants as the sample for all subsequent analyses (Table 1).

While these 61 selected DMM plants hold comparatively rich botanical information, the details available is often fragmentary and unevenly distributed across the categories (**Research data, Data file 3**, https://figshare.com/s/2e0f64b5794892ed4679). This is also illustrated in Table 1 by a cross-check of the information available for the morphological, ecological or geographical criteria.

Analysis of the historical medicinal data shows that the 50 JC plant names selected for analysis are associated with 242 unique use records. These involve 124 different medicinal plant uses distributed across 20 of the 21 medicinal use groups, with dermatological uses being by far the most frequent and diverse use group (31 different uses) (**Research data, Data file 4**, https://figshare.com/s/2e0f64b5794892ed4679).

### 3.2 Modern dataset

Implementing stage 2 of the methodology provides a list of the potential botanical identities reported in the literature (candidate plants) for the 50 JC plant names (Supplementary material, Document S1). Analysis shows that, on average, five different scientific names had been suggested in the literature for each single JC plant name (including cases of incomplete or misspelled scientific names, synonyms, and homonyms) (**Research data, Data file 5**, https://figshare.com/s/2e0f64b5794892ed4679). MPNS (2023) validation of these names resolved ambiguities and provided the currently accepted scientific name for each candidate plant, as well as all known synonyms. This reduced the average number of candidate plants derived from the literature to two species per JC plant name. Validation of the scientific names in use in the literature reveals that the majority were either outdated synonyms, incomplete or inconsistent names, or names citing unrecognisable authors. In five cases the identity of the intended plant could not be established and no accepted scientific name was allocated. The cleaned and analysed list of candidate plant identities for the 50 JC plant names (61 DMM plants) contains 130 taxonomically validated accepted scientific names.

Botanical and medicinal information was collected for these 130 candidate plants from modern sources as described in methodology stage 3 (**Research data, Data files 6, 7, 8**, https://figshare.com/s/2e0f64b5794892ed4679). In stage 4, data matrices of the botanical and the medicinal information were built enabling data from historical and modern sources to be compared and, in stage 5, subjected to statistical evaluation.

### 3.3 Comparative analyses and statistical evaluation

Juxtaposing the historical data collected for the 50 JC plant names (61 DMM plants) with the corresponding data from modern sources for the 130 candidate plants provides the data basis for the comparative analyses. Because some candidate plants were reported as possible identities for more than one JC plant name, the total number of comparative data records is 141 (Table 2).

In the comparative botanical analysis, we observed quite variable levels of congruence between the descriptive data culled from modern floras and those descriptions obtained from DMM. In total, 1213 individual data items from the historical text were compared with a corresponding data value taken from modern floras (**Research Data, data file 9**, https://figshare.com/s/2e0f64b5794892ed4679). Of these, 435 (36%) were classed as “good matches” (see section 2.2, step 5.1 for definition), 290 (24%) as “partial matches” and 83 (7%) as “no matches”. A significant number of comparisons, 405 (33%), proved to be impossible because data were “absent”. That this occurred was not a surprise since a modern flora might not be expected to record equivalent observations to those mentioned in DMM, but the absence of data observations clearly prevented comparison of some features. The high percentage of such cases impacted on the overall comparisons made. A further 21 of the 130 taxa could not be assessed at all since they were not listed in either FT or FC. Given our assumptions regarding a mainly Eastern Mediterranean origin of the herbal *materia medica* cited in DMM (see section 1), some of these taxa would presumably be less qualified as candidate plants.

In the comparative analysis of medicinal uses, 37 JC plant names (74%) showed congruence in one or more medicinal categories with at least one of the relevant suggested candidate plants. Viewed as a whole, however, the distribution of the 21 medicinal use categories differed considerably between the historical data from JC and the uses of the candidate plants as described in modern literature. For two JC plant names, no medicinal plant uses were reported at all in any of the modern sources (**Research data, Data file 10**, https://figshare.com/s/2e0f64b5794892ed4679).

The results of the comparative botanical and medicinal analyses were statistically evaluated to establish which of the candidate plants are the most likely identity of each JC plant name (**Research data, Data file 11**, https://figshare.com/s/2e0f64b5794892ed4679).

Following evaluation of the botanical analysis, a total of 37 (26%) of 141 comparative records of candidate plants demonstrated “Strong” botanical congruence with their respective JC plant names, 51 (36%) candidate plants demonstrated “Moderate” congruence, and 33 (23%) demonstrated “Weak” congruence. A further 20 (14%) candidate plants could not be assessed due to lack of comparable data (not listed in FT or FC, see above) (Table 2).

In the equivalent evaluation of medicinal uses, 53 (38%) candidate plants demonstrated “Strong” (score = 3) congruence between historically reported uses from JC and the modern sources analysed. 20 (14%) candidate plants demonstrated “Moderate” (score = 2) congruence, and 33 (23%) demonstrated “Weak” (score = 1) congruence. 35 (25%) candidate plants were not found in the modern medicinal literature (Table 2). Of note, 35 (66%) of the candidate plants assessed as “Strong” in terms of their medicinal uses were not associated with any “Strong” candidate plants as a result of our botanical analysis.

As mentioned, the medicinal uses analysis was evaluated as supplementary evidence only. However, in terms of internal consistency between the two analyses, 32 (23%) candidate plants demonstrated consistent results across both analyses (e.g. “Strong” for botanical congruence and “Strong” for medicinal uses congruence), and 24 (17%) demonstrated inconsistent results (e.g. “Strong” for botanical congruence, but “Weak” for medicinal uses congruence). 41 (29%) could not be assessed due to missing data in one or both analyses (Table 2).

### 3.4 Reflection and revision

With the goal of conducting a final assessment and revision of our methodology, workflows and data analyses, key outputs of the workshop were 1) a list of the challenges associated with applying the methodology developed within this study to historical texts in different languages and from different cultural contexts, and 2) specific recommendations on how to address challenges linked with the application of the methodology and how to improve it (for details, see section 4.4.2 and Table 3).

## 4 Discussion

### 4.1 General considerations

This study should be seen as taking an experimental approach in developing a systematic methodology for more objectively assessing the reliability of existing (and potentially new) identifications of plants cited in ancient manuscripts. We do not claim to have accumulated exhaustive datasets, nor to have the final word on the plant identities achieved. The limitations inherent in our study of JC were.

1) Only plants with rich botanical information in DMM were included in the comparative analysis of botanical and medicinal traits. We disregarded species for which DMM provided less detailed descriptions;
2) We compared the historical descriptions of the plants and their uses only against a subset of modern species which had been previously suggested as candidates. We did not compare the historical descriptions with the entire (Eastern) Mediterranean flora;
3) We only used Floras that cover the native plants of Turkey, the Eastern Aegean islands and Cyprus. We were thus unable to assess the likelihood of suggested candidate plants which are native to other regions;
4) Apart from Mediterranean ethnobotanical sources and global clinical trials, plant uses were only captured from modern herbal textbooks popular in the United Kingdom. Herbal textbooks from other European countries may have indicated further uses.

Dealing with a recipe text such as JC added complexity. Historical texts of this genre usually focus on medicinal practice rather than providing plant descriptions or images. It was therefore necessary to access culturally and historically associated botanical or pharmacognostic texts containing plant descriptions. This study relied on DMM as the source of information on the plants used in JC. Use of DMM is supported by its age-long importance and unique position as a pharmaco-botanical reference text in the Greek line of tradition (Temkin, 1962; Riddle, 1985; Collins, 2000). Our study demonstrates the close relationship between the herbal *materia medica* in DMM with those used in JC (see section 4.2).

The significance of the results of this study depends on the quality of the resources used. Wellmann’s reconstruction of Dioscorides’ work is based on several manuscripts of the five book recension of DMM containing more than 600 plants, and is thus the most comprehensive source (Collins, 2000; Janick and Stolarczyk, 2012). Nonetheless it contains ambiguities (Evergetis and Haroutounian, 2015). Illustrated versions of DMM containing plant drawings (e.g. Vienna Dioscorides, 1998 and 1999 or the Naples Dioscorides, 2000) may have offered additional evidence but postdate the autograph and would have added further potential cause for confusion and misjudgement.

### 4.2 Historical consistency of plant names

The work of John the Physician (Ioannes Arkhiatros) is regarded as an outstanding text of the *iatrosophia* (Temkin, 1962) which is a typical genre of recipe texts of Byzantine origin (Garzya, 2003; Touwaide, 2007; Lardos et al., 2011). This study points to the possible importance of the text as a resource for ethnopharmacological research because of the empirical knowledge contained. An exhaustive investigation of the plants and their uses in JC was not in the scope of this study. We note, however, a remarkable historical consistency in the use of plant names. Most of the JC plant names have the same name as in DMM (see section 3.1). This substantiates a cultural continuity regarding herbal *materia medica* in Greek-speaking societies from Antiquity until the late Byzantine era (a period of at least 13 centuries). It also suggests a remarkable stability of the pharmacopoeia from Dioscorides to writers of the later Byzantine era. These findings also support our argument (see section 1) for using DMM as the pharmacognostic reference.

### 4.4 Thoughts regarding transferability of the methodology

Considering the wider goal of this study “to provide a transferable methodology”, we separate the discussion here into issues relating specifically to our case study (section 4.4.1) and issues concerning wider application of the methodology (section 4.4.2).

#### 4.4.1 Points specific to the present case study

Here we focus on the botanical and medicinal comparative analysis, highlighting the more important points concerning the investigation of JC or DMM, respectively, and their cultural and geographical setting.

One issue frequently met when undertaking botanical comparisons was that DMM often described the characteristics of a given plant by comparing it to another plant. The shape of the leaves of ἀσφóδελος (II, 169), for example, are compared to the leaves of πράσον κεφαλωτόν (II, 150); the morphology of the flower of ὑοσκÚαμος (IV, 68) likened to the flower of ῥóα (I, 110); and the fruit capsule of μελάνθιον (III, 79) compared to that of μήκων (IV, 64) (Beck, 2005). Evidently this prevented us from obtaining explicit diagnostic features for comparison with modern plant descriptions. The reference plants used in the comparison are often well-known crop plants or other prominent plants, for which DMM usually does not provide any descriptions at all. A systematic study of the plant comparisons in DMM might shed further light on the understanding of plant morphology during Greek-Roman antiquity. A second issue observed was that DMM described plants at different developmental stages from the descriptions of plants found in FT or FC. For example, DMM describes only the “stem”, leaves and fruits of δρακοντíα μεγáλη (II, 166) (Beck, 2005), but not its flowers, preventing us from making a comparison with the showy flowers of the suggested candidate *Dracunulus vulgaris* L. or any other plant in the Araceae.

A further complication impacting botanical assessment was the under-differentiation observed for some JC plant names (194 JC plant names are associated with 252 DMM plants, see section 3.1). Where one JC plant name leads to two or more DMM plants a question arises how users of the text dealt with such vague specifications when sourcing herbs for the recipes. There are two possible explanations: 1) In case of morphologically similar plants of the same genus, distinguishing between different species is sometimes of minor importance in traditional medicinal systems, since they are often used interchangeably. Such cases are regarded as ethnotaxa, in which closely related plant species have identical uses (Leonti et al., 2015); 2) Many of these plants can be considered plant complexes: a group of different species consisting of one label plant and several substitutes sharing the same basic name and having common qualities and uses (Linares and Bye, 1987). In some cases this is made more explicit by DMM: The JC plant name χαμαíπιτυς (Table 1) is listed in DMM III, 158 as having three different types (χαμαíπιτυς, - έτέρα, - τρíτη). Dioscorides writes that all these plants have the same properties, although suggesting that the first mentioned was stronger (Beck, 2005). Another example is ἀρνóγλωσσον with its two types, μεῖζον and μικρóν (DMM II, 126), of which one (μεῖζον) is said to be more useful (Beck, 2005).

Our study relied on suggested candidate plants for the botanical assessment of historical plant names. This, however, involves the risk of not capturing all possible candidate plants. Other species, particularly those from within the same genus, might prove to be equally strong candidates. For example, given the number of matched plant characteristics, ἵππουρις (DMM IV, 46) might also be identified as *Equisetum palustre* L. (Davis et al., 1965–85: 1, 32), as an alternative to the *Equisetum* species suggested in the literature (Table 2). Similarly, εὐπατώριος (DMM IV, 41) would match equally well to *Agrimonia repens* L. (Davis et al., 1965–85: 4, 74) rather than only to *A. eupatoria* L., or the ὑοσκύαμος type ἄνθη ὐποπóρφυρα (DMM IV, 68) might match to *Hyoscyamus reticulatus* L. Specific recommendations to this issue are available in Table 3, under step 2.1.

For the medicinal comparisons it was striking that historical and modern plant uses often differed considerably from each other. Such disparities may be seen to disqualify the attribution of the historical plant name with that particular candidate plant. However, in the cases documented, the botanical analysis often qualifies the species as a likely candidate (Table 2) and changes in the use of medicinal plants over time is considered feasible. Substantial temporal changes in how plants are used medicinally has previously been observed in Central Europe over the last 2000 years (Dal Cero et al., 2023).

A key assumption in our work is that the flora of the study region is broadly similar, at least in the presence and absence of plant species, to the times of Dioscorides and John the Physician. The substantial body of archaeobotanical research carried out on ancient seeds recovered from archaeological sites, in many cases identifiable to species, demonstrates that ancient flora can usually be identified by comparison with nearby modern flora (e.g. Rivera et al., 2012). Evidence from pollen and other sources demonstrates periods of aridity and resulting changes in vegetation composition during the Holocene (the last 10,000 years), but also highlights human impact as the most important factor in vegetation change (Roberts et al., 2011). Overall there is evidence that the abundance of individual plant species might change, but not of large-scale migrations of plants, except when moved by humans.

#### 4.4.2 General points to the application of the methodology

Here we consider the wider application of the methodology to analyse historical texts from different cultural or linguistic settings. Many issues are inherent in all ethnopharmacological investigations of historical texts and should be considered alongside the checklist for historical studies in the consensus paper on best-practice for ethnopharmacological field studies (Heinrich et al., 2018). In Table 3 the points are discussed individually and recommendations made as to how to address these issues.

Especially noteworthy were the many idiosyncrasies of working in an international multidisciplinary team. In addition to the differing practices of institutions located in different countries, challenges to be negotiated included differences between specialist work modalities, the particular needs and emphases within each discipline, and differing understanding and perspectives in both devising the methodological approach and interpreting the outcomes. In this respect we recommend assessing whether institutional data management plans and disciplinary practices are compatible across the project. Some team members may need to use specialist software, data may need to be compatible for transfer between formats even where mainstream software packages are used. Some circumstances, such as bidirectional text or non-Latin alphabets, may render some standard software unstable. An ongoing dialogue between all team members and regular discussion of plans and outputs at each stage are essential.

### 4.3 Evaluation of the analytical and statistical results

Detailed plant descriptions from historical texts were a basic pre-requisite for the application of our methodology. Only plants exhibiting significant morphological information could be selected for botanical analysis (Methodology step 1.4). Altogether 50 (26%) of the 194 JC plant names, corresponding to 61 (24%) of 252 DMM plants, met this condition and results of the comparative analyses are restricted to this group of plants (**Research data, Data file 2**, https://figshare.com/s/2e0f64b5794892ed4679). It is therefore necessary to observe that only a modest percentage of the plants mentioned in JC or DMM, can be identified with any degree of certainty based on written botanical criteria. On the other hand, many of the 117 plants (47%) lacking detailed botanical information appear to be crop plants or common aromatic herbs, suggesting that these plants were well known and did not require detailed descriptions.

With the above limitations in mind, the comparative botanical analyses show a degree of similarity between the historical and the modern descriptions of plant characteristics could be established in the majority of comparisons undertaken (60%, i.e. 36% “good match” and 24% “partial match”). Only in 83 cases (7%) were the descriptions being compared considered not to match (**Research data, Data file 9**, https://figshare.com/s/2e0f64b5794892ed4679). This, however, does not enable us to conclude that most of the suggested species are strong candidates. Good or partial matches are usually clustered around one or two candidates in each case.

A significant issue was that in 33% of cases characteristics described in DMM were not mentioned in FT or FC and were thus recorded as “absent”, precluding any comparisons. This weakness is illustrated in Table 3 step 5.1. For future application of our method we suggest considering also using less specialised texts of plant descriptions, or referring to living plants or herbarium specimens. However this would add significantly to the time required for the study.

A statistical proximity was assigned to each viable candidate plant recording a “high”, “moderate” or “weak” measure of confidence of that candidate being the correct attribution following both the botanical analysis and the medicinal analysis. These assessments were based on qualitative assessment of the dataset as a whole. As shown in Table 2, for 27 (82%) of the 33 JC plant names or 26 (74%) of the 35 DMM plants, respectively, for which more than one candidate plant was available, the respective candidates were distributed over two or more categories of the botanical analysis (“Strong”, “Moderate”, “Weak”, “Not contained”). In some cases, the candidates are distributed over all four categories, such as the 8 candidates from 5 different genera of the JC plant name ἀμáραντον. Thus our method, in the majority of cases, was able to detect those candidate(s) with a higher likelihood of being the correct attribtuion in a pool of suggested candidates. In only 6 (18%) or 9 (26%) of cases, did the method fail to differentiate between alternative candidates. For example, the JC plant name χαμαíμηλον (*khamaimelon*) had previously been associated with two potential species (*Matricaria chamomilla* L. and *Anthemis chia* L.). Each demonstrates “Strong” botanical congruence with the JC plant name. In such cases, the medicinal uses comparison provides useful additional guidance. For example, *M. chamomilla*, with a high congruence in the medicinal uses, is considered to be a more likely candidate than *A. chia*, for which no modern uses were found.

## 5 Conclusions

This study outlines a workflow and a set of procedures for use by scholars researching historical texts who seek a more objective and evidence-based approach to establishing the potential identities of the plants cited in those texts. It involves a comparative analysis using descriptions included in the historical text and descriptions taken from modern scientific sources, enabling calculation of a relative likelihood of each candidate (suggested) species being the correct identity. This approach offers a means to challenge past assumptions regarding the identity of plants described in Greek texts from antiquity and medieval times. It also contributes to the creation of a Greek plant name census, providing the tools for addressing the plant names in such texts from Antiquity to the Byzantine era and beyond.

Despite the experimental nature of our methodology and its limitations, the results demonstrate that our approach allows certain conclusions to be drawn about the validity of alternative (and previously suggested) candidate plants as well as to distinguish between different candidates of the same historical plant name. This provides *i)* a rational basis to make an informed choice when searching for candidate plants from historical texts as starting points for natural product based research, and *ii)* a higher security in studies about the evolution of herbal pharmacopoeias.

The methodology is fully documented to facilitate its application to historical texts of most cultural or linguistic backgrounds. To this end, the study also provides a checklist (Table 3) of the major challenges of each process step and offers recommendations as to how to address those issues. We hope that future applications of the methodology in other contexts will extend and improve upon the workflows and procedures presented here. We also hope to stimulate further interdisciplinary discourse among all relevant disciplines with regard to investigation of *materia medica* in historical texts.

## Supplementary Material

**Appendix A. Supplementary data**

Supplementary data to this article can be found online at https://doi.org/10.1016/j.jep.2023.117622.

Document S1

Figure S1

Figure S1 legend

Table S1

Table S2

Table S3

## Figures and Tables

**Table 1:** The 50 plant names in John’s Commentary (JC) and the corresponding 61 plants of Dioscorides’ *De Materia Medica* (DMM) with rich morphological information.

Lemma tag	JC plant name		Connection with DMM	DMM plant name		DMM type		DMM chapter	Life form	Habit	Height	Stem	Leaf	Flower	Fruit	Seed	Root	Habitat	Distribut.	Origin
JCLP_003	άγριοσταφίδα	*agriostaphida*	Synonym^1^	σταφίς άγρία	*staphis agria*	–	–	IV, 152				x	c,x	c,x		c,x				
JCLP 004	άδίαντον	*adianton*	Same name	άδίαντον	*adianton*	–	–	IV, 134			x	x	c	x		x	x			
JCLP_008	άκάνθη άσπρη	*akanthe aspre*	Synonym^2^	άκανθα λευκή	*akanthe leuke*	–	–	III, 12				x	c	x		c	c	x		
JCLP_012	άλόη	*aloe*	Same name	άλόη	*aloe*	–	–	III, 22				x	c,x	c,x	c		x		x	x
JCLP_013	άλυσσος	*alyssos*	Same name	άλλυσσον	*alysson*	–	–	III, 91		x		x	x		x	x		x		
JCLP_078	άμάραντον	*amaranton*	Same name	άμάραντον,	*amaranton,*	–	–	IV, 57		x			c,x	x			x	x		
				άλίχρυσον	*elikhryson*															
JCLP_016	ἄμπελος	*ampelos*	Same name	άμπελος άγρια	*ampelos agria*	–	–	V, 2; IV, 181		x		c, x	c	x	x	x				
JCLP_022	άριστολοχία	*aristolokhia*	Same name	άριστολοχεία	*aristolokheia*	στρογγύλη,	*strongyle,*	III, 4		x		x	c,x	c,x	x		c,x	x		
						θύλεια	*thyleia*													
						μακρά, άρρην	*makra, arren*	III, 4		x		x	c,x	c,x	x		c,x	x		
JCLP_023	άρνόγλωσσον	*arnoglosson*	Same name	άρνόγλωσσον	*arnoglosson*	μικρόν	*mikron*	II, 126			x	x	x	x		x	x			
						μειζόν	*meizon*	II, 126			x	x	x	x		x	x			
JCLP_025	άσαρ	*asar*	Same name	ἄσαρον	*asaron*	–	–	I, 10					c	c,x	c		c,x			
JCLP_027	άσφόδελος	*asphodelos*	Same name	άσφόδελος	*asphodelos*	–	–	II,169				x	c	x			x			
JCLP_035	βετονίκη	*betonike*	Same name	βεττονίκη, in	*bettonike, in*	–	–	IV, 1		x	x	x	c,x	c,x			c,x	x		
				κέστρον	*kestron*															
JCLP_037	βρυωνία	*bryonia*	Same name	άμπελος μέλαινα,	*ampelos melaina,*	–	–	IV, 183	x			x	c	x			c,x			
				βρυωνία	*bryonia*															
JCLP_039	γεντιανή	*gentiane*	Same name	γεντιανή	*gentiane*	–	–	III, 3			x	x	c,x		x	c	c	x		
JCLP_043	γλυκόριζον	*glykorizon*	Same name	γλυκύρριζα	*glykyrrhiza*	–	–	III, 5	x	x	x		c,x	c	c,x		c		x	
JCLP_048	δαυκίν	*daukin*	Same name	δαοκος	*daukos*	κρητικός	*Kretikos*	III, 72			x		c	c,x	x		x	x		
			Synonym^1^	σταφυλίνος	*staphylinos*	άγριος	*agrios*	III, 52				x	c	c,x			x			
JCLP_051	δρακοντία	*drakontia*	Same name	δρακοντία	*drakontia*	μεγάλη	*megale*	II, 166			x	x	c		x		c,x	x		
						μικρά	*mikra*	II, 167			x	x	c		x		c,x			
JCLP_057	εύπατώριος	*eupatorios*	Same name	εύπατόριος	*eupatorios*	–	–	IV, 41		x	x	x	c,x			x				
JCLP_069	ϊππουρις	*ippouris*	Same name	ϊππουρις	*ippouris*	–	–	IV, 46	x	x	x	x	x				x	x		
JCLP_070	ἶρις	*iris*	Same name	ἶρις	*iris*	–	–	I, 1					c,x	x			x			x
JCLP_076	κάππαρις	*kapparis*	Same name	κάππαρις	*kapparis*	–	–	II,173	x	x		c,x	c	c,x	x	c,x				
JCLP_081	καυκαλίδα	*kaukalida*	Same name	καυκαλίς	*kaukalis*	–	–	II, 139			x	x	c,x	x						
JCLP_084	κενταύριον	*kentaurion*	Same name	κενταύριον	*kentaurion*	μέγα	*mega*	III, 6		x	x	c,x	c,x	c,x	c,x		x	x	x	
						λεπτόν	*lepton*	III, 7		c	x	x	c,x	c,x	c		x	x	x	
JCLP_095	κόνυζα	*konyza*	Same name	κόνυζα	*konyza*	μικρά	*mikra*	III, 121		x	x		c,x	x	x					
						μείζων	*meizon*	III, 121		x	x		c,x	x	x					
JCLP_104	κυκλάμινος	*kyklaminos*	Same name	κυκλάμινος	*kyklaminos*	–	–	II, 164				x	c,x	c,x			c,x			
JCLP_149	κύπερος	*kyperos*	Same name	κύπερος	*kyperos*	–	–	I, 4			x	c,x	c			x	c,x	x	x	
JCLP_107	κύπρος	*kypros*	Same name	κύπρος	*kypros*	–	–	I, 95	x				c,x	x		c				
JCLP_109	κώνειον	*koneion*	Same name	κώνειον	*koneion*	–	–	IV, 78				c	c	x		c	x			
JCLP_113	λειχήνη	*leikhene*	Same name	μυρσίνη άγρια,	*myrsine agria,*	–	–	IV, 144		x	x	x	c,x		x	x	x	x		
				λειχήνη	*leikhene*															
JCLP_122	μανδραγόρας	*mandragoras*	Same name	μανδραγόρας	*mandragoras*	θήλυς, μέλας	*thelys, melas*	IV, 75				x	c,x		c,x	c	x			
						άρρην, λευκός	*arren, leukos*	IV, 75				x	c,x		c,x	c	x			
JCLP_126	μελάνθιον	*melanthion*	Same name	μελάνθιον	*melanthion*	–	–	III,79	x		x		c		c,x	x				
JCLP_165	πολυπόδιον	*polypodion*	Same name	πολυπόδιον	*polypodion*	–	–	IV, 186		c		x	c				x	x		
JCLP_241	πράσιον	*prasion*	Same name	πράσιον	*prasion*	–	–	III, 105	x	x		x	x	x	x			x		
JCLP_174	ρ́άμνος	*ramnos*	Same name	ρ́άμνος	*ramnos*	μέλας	*melas*	I, 90	x	c,x		x	x		x			x		
JCLP_180	ρ́οδάφνη	*rodaphne*	Same name	νέριον,	*nerion,*	–	–	IV, 81	x				c,x	c	x		x	x		
				ροδοδάφνη	*rododaphne*															
JCLP_242	ρούδιν	*roudin*	Synonym^1^	ῥοῦς	*rous*	–	–	I, 108	x		x		x		c,x			x		
JCLP_191	σιδερίτις	*sideritis*	Same name	σιδερίτις	*sideritis*	–	–	IV, 33	x		x	x	c	c		x		x		
JCLP_196	σκολοπένδριον	*skolopendrion*	Same name	ἄσπληνος,	*asplenos,*	–	–	III, 134		x		x	c,x					x
				σκολοπένδριον	*skolopendrion*													
JCLP_198	σκορπίουρον	*skorpiouron*	Same name	ἡλιοτρόπιον τὸ μέγα,	*heliotropion to mega,*	–	–	IV, 190		x			c	c, x			x	x
				σκορπίουρον	*skorpiouron*													
JCLP_204	στρύχνον	*strykhnon*	Synonym^1^	στρύχνον	*strykhnon*	–	–	IV, 70	x	x			c,x		x			
	(βρωμοβότανον)	*(bromobotanon)*		κηπαĩον	*kepaion*													
JCLP_210	τετράγκανθον	*tetrangkanthon*	Synonym^1^	τραγάκανθα	*tragakantha*	–	–	III, 20		x		x	x				x	
JCLP_214	τρίβολον	*tribolon*	Same name	τρίβολος	*tribolos*	ένυδρος	*enydros*	IV, 15		x		x	x		c			x
JCLP_215	τριφύλλι	*triphylli*	Same name	τρίφυλλον	*triphyllon*	–	–	III, 109	x	x	x			x		x	x	
JCLP_217	ύοσκύαμος	*hyoskyamos*	Same name	ύοσκύαμος	*hyoskyamos*	άνθη	*anthe*	IV, 68	x			x	c,x	c,x		c,x		
						υποπόρφυρα	*hypoporphyra*											
						άνθη	*anthe*	IV, 68	x			x	c,x	c,x		c,x		
						μηλινοειδή	*melinoeide*											
						τρίτος, άνθη	*tritos, anthe*	IV, 68	x			x	c,x	c,x		c,x		x
						λευκά	*leuka*											
JCLP_221	φλόμος	*phlomos*	Same name	φλόμος	*phlomos*	λευκή,	*leuke, thyleia*	IV,		x		x	c,x	c,x		x	x	x
						θηλεία		103.1										
JCLP_225	χαμαιδάφνη	*khamaidaphne*	Same name	χαμαίδαφνη	*khamaidaphne*	–	–	IV, 147	x		x	x	c	x	x			x
JCLP_227	χαμαίμηλον	*khamaimilon*	Same name	χαμαίμηλον, in	*khamaimelon, in*	–	–	III, 137		x	x		x	c,x				x
				άνθεμίς	*anthemis*													
JCLP_228	χαμαίπιτυς	*khamaipitys*	Same name	χαμαίπιτυς	*khamaipitys*	χαμαίπιτυς	*khamaipitys*	III, 158	x	x			c,x	x		x	c	
						ετέρα	*hetera*	III, 158			x	x	c,x	x		x		
						τρίτη	*trite*	III, 158			x	x	x	x		x		
JCLP_231	χελιδόνιον	*khelidonion*	Same name	χελιδόνιον μέγα	*khelidonion mega*	–	–	II, 180		x	x		c,x	c	c,x	c,x	x	
JCLP_235	ψύλλεον	*psylleon*	Same name	ψύλλιον	*psyllion*	–	–	IV, 69		x	x		c,x	x		x		

**Table columns:** **Lemma tag**: Unique identifier of the lemmatised JC plant name. **JC plant name**: Spelling variants of the respective name also mentioned in JC are not shown. **Connection to DMM**: Same name – JC plant name is identical with DMM plant name; Synonym – JC plant name is regarded as a synonym of the respective DMM plant name, based on 1) Gennadios (1914) and Langkavel (1866), or 2) the linguistic context. Corresponding cases are indicated (Synonym1 or Synonym2, respectively). **DMM plant name**: Name in DMM according to Beck (2005) and cross-checked with Wellmann (1907). **DMM type**: Name of “types” of the respective DMM plant as stated in Wellmann (1907). **DMM chapter**: Book volume and chapter in DMM according to Beck (2005). **Botanical information**: Botanical information stated in DMM for the respective plant, including morphological (life form, habit, height, stem, leaf, flower, fruit, seed, root), ecological (habitat), and geographical (distribution or origin – in case of traded goods) information (x). Some morphological characteristics are described in DMM only by comparison to another plant “c”, or in combination with specific botanical information “c,x”.

**Table 2:** Results of the statistical evaluation of the botanical and the medicinal comparative analyses. The 50 plant names in John’s Commentary (JC) and the associated 61 plants in Dioscorides’ *De Materia Medica* (DMM) are listed with their respective candidate plants (CP) classified according to the results of the statistical evaluation of the botanical analysis (“Strong”, “Moderate”, “Weak candidate”, or “Not contained in FT or FC”). The results of the statistical evaluation of the medicinal analysis are indicated in parenthesis for each CP (3 – “Strong”, 2 – “Moderate”, 1 – “Weak candidate”, 0 – No comparison with the JC plant uses was possible, because no uses were reported for the respective CP).

Lemma tag	JC plant name	DMM plant name	# Candidate plants (CPs)	Strong candidate	Moderate candidate	Weak candidate	Not contained in FT or FC
JCLP_003	όγριοσταφίδα	σταφίς άγρία	1			*Delphinium staphisagria* L. (1)	
JCLP_004	αδίαντον	άδίαντον	1			*Adiantum capillus-veneris* L. (3)	
JCLP_008	άκάνθη άσπρη	ἄκανθα λευκή	4		*Onopordum bracteatum *Boiss. & Heldr. (1) *Picnomon acarna* (L.) Cass. (1)		*Echinops graecus* Mill. (0) *Cirsium ferox* (L.) DC. (0)
JCLP_012	αλόη	άλόη	2		*Aloe vera* (L.) Burm.f. (2)		*Aloe perfoliata* L. (2)
JCLP_013	ἄλυσσος	ἄλλυσσον	3	*Fibigia clypeata* (L.) Medik. (0)	*Odontarrhena alpestris* (L.) Ledeb. (0)	*Scutellaria galericulata* L. (1)	
JCLP_078	άμάραντον	άμάραντον, έλίχρυσον	8	*Helichrysum stoechas *subsp. *barrelieri* (Ten.) Nyman (1) *Helichrysum italicum *(Roth) G.Don (1) *Helichrysum orientale* (L.) Gaertn. (0)	*Petrosedum ochroleucum* (Chaix) Niederle (0) *Sedum eriocarpum* Sm. (0)	*Teucrium polium* L. (3)	*Helichrysum stoechas* (L.) Moench (1) *Tanacetum annuum* L. (0)
JCLP_016	άμπελος	άμπελος άγρια	3			*Clematis vitalba* L. (1) *Dioscorea communis *(L.) Caddick & Wilkin (3) *Vitis vinifera* L. (3)	
JCLP_022	άριστολοχεία	άριστολοχεία: μακρά, ἄρρην	3	*Aristolochia parvifolia* Sm. (3)		*Aristolochia sempervirens* L. (3)	*Aristolochia fontanesii* Boiss. & Reut. (0)
JCLP_022	άριστολοχεία	άριστολοχεία: στρογγύλη, θήλεια	3		*Aristolochia pallida* Willd. (0) *Aristolochia rotunda* L. (3)	*Aristolochia sempervirens* L. (3)	
JCLP_023	άρνόγλωσσον	άρνόγλωσσον: μεΐζον	3	*Plantago major* L. (3)		*Plantago media* L. (3)	*Plantago asiatica* L. (3)
JCLP_023	άρνόγλωσσον	άρνόγλωσσον: μικρόν	3			*Plantago lagopus* L. (3) *Plantago lanceolata* L. (3) *Plantago maritima* L. (1)	
JCLP_025	ἄσαρ	ἄσαρον	1	*Asarum europaeum* L. (3)			
JCLP_027	ασφόδελος	άσφόδελος	3		*Asphodelus aestivus* Brot. (2) *Asphodelus fistulosus* L. (1) *Asphodelus ramosus* L. (1)		
JCLP_035	βετονίκη	βεττονίκη, in κέστρον	2			*Sideritis cretica* L. (0)	*Betonica alopecuros* L. (0)
JCLP_037	βρυωνία	άμπελος μέλαινα, βρυωνία	3		*Bryonia alba* L. (3) *Dioscorea communis *(L.) Caddick & Wilkin (3)	*Bryonia cretica* L. (3)	
JCLP_039	γεντιανή	γεντιανή	1		*Gentiana lutea* L. (3)		
JCLP_043	γλυκόριζον	γλυκύρριζα	2	*Glycyrrhiza echinata* L. (3) *Glycyrrhiza glabra* L. (3)			
JCLP_048	δαυκίν	δαῦκος: Κρητικός	1				*Athamanta cretensis* L. (0)
JCLP_048	δαυκίν	σταφυλίνος: άγριος	3		*Daucus carota* L. (3)	*Pastinaca lucida* L. (0)	*Daucus guttatus* Sm. (0)
JCLP_051	δρακοντία	δρακοντία: μεγάλη	5	*Dracunculus vulgaris*	*Arum dioscoridis* Sm.	*Arisarum vulgare* O.	
				Schott (2)	(3) *Arum italicum* Mill. (2)	Targ.Tozz. (2) *Arum maculatum* L. (2)	
JCLP_051	δρακοντία	δρακοντία: μικρά	5		*Arisarum vulgare* O. Targ.Tozz. (2) *Arum dioscoridis* Sm. (3) *Arum italicum* Mill. (2)	*Arum maculatum* L. (2) *Dracunculus vulgaris* Schott (2)	
JCLP_057	εύπατώριος	εύπατόριος	1	*Agrimonia eupatoria* L. (1)			*(continued on next page* *)*
JCLP_069	ἵππουρις	ἵππουρις	8	*Equisetum ramosissimum*	*Equisetum arvense* L.	*Ephedra major* Host	*Ephedra fragilis* Desf. (2)
				Desf. (3)	(3)	(2)	
				*Equisetum telmateia* Ehrh.	*Equisetum sylvaticum*		
				(3)	L. (1)		
				*Ephedra foeminea* Forssk.	*Equisetum fluviatile* L.		
				(0)	(0)		
JCLP_070	ἶρις	ἶρις	2			*Iris* × *germanica* L. (3) *Iris fiorentina* L. (3)	
JCLP_076	κάππαρις	κάππαρις	1	*Capparis spinosa* L. (3)			
JCLP_081	καυκαλίδα	καυκαλίς	3		*Pimpinella saxifraga* L. (1) *Tordylium aegyptiacum* (L.) Poir. (1)	*Papaver* L. (0)	
JCLP_084	κευταύριον	κενταύριον: λεπτόν	1	*Centaurium erythraea* Rafn (2)			
JCLP_084	κευταύριον	κενταύριον: μέγα	1				*Rhaponticoides centaurium *(L.) M.V.Agab. & Greuter (0)
JCLP_095	κόνυζα	κόνυζα: μείζων	1	*Dittrichia viscosa* (L.) Greuter (3)			
JCLP_095	κόνυζα	κόνυζα: μικρά	1		*Dittrichia graveolens *(L.) Greuter (1)		
JCLP_104	κυκλάμινος	κυκλάμινος	5	*Cyclamen graecum* Link		*Cyclamen cyprium*	*Cyclamen purpurascens*
				(0) *Cyclamen hederifolium* Aiton (0) *Cyclamen persicum* Mill. (3)		Kotschy (3)	Mill. (0)
JCLP_149	κύπερος	κύπερος	2	*Cyperus longus* L. (1) *Cyperus rotundus* L. (2)			
JCLP_107	κύπρος	κύπρος	1			*Lawsonia inermis* L. (1)	
JCLP_109	κώνειον	κώνειον	1	*Conium maculatum* L. (3)			
JCLP_113	λειχήνη	μυρσίνη άγρια, λειχήνη	1	*Ruscus aculeatus* L. (1)			
JCLP_122	μανδραγόρας	μανδραγόρας:	2		*Mandragora*		
		ἄρρην, λευκός			*autumnalis* Bertol. (3) *Mandragora officinarum* L. (3)		
JCLP_122	μανδραγόρας	μανδραγόρας:	2			*Mandragora*	
		θήλυς, μέλας				*autumnalis* Bertol. (3) *Mandragora officinarum* L. (3)	
JCLP_126	μελάνθιον	μελάνθιον	2	*Nigella sativa* L. (3)		*Nigella damascena* L. (2)	
JCLP_165	πολυπόδιον	πολυπόδιον	3		*Polypodium cambricum*		*Phegopteris connectilis*
					L. (3) *Polypodium vulgare* L. (3)		(Michx.) Watt (0)
JCLP_241	πράσιον	πράσιον	1	*Marrubium vulgare* L. (3)			
JCLP_174	ρ́άμνος	ρ́άμνος: μέλας	3		*Rhamnus oleoides* L.		*Frangula purshiana* (DC.)
					(1)		A.Gray ex J.G.Cooper (0) *Rhamnus lycioides* L. (0)
JCLP_180	ρ́οδάφνη	νέριον, ρ́οδοδάφνη	1		*Nerium oleander* L. (3)		
JCLP_242	ρ́ούδιν	ῥοῦς	1		*Rhus coriaria* L. (3)		
JCLP_191	σιδερίτις	σιδερίτις	8	*Sideritis montana* L. (1)	*Sideritis cypria* Post (1)		*Sideritis scardica* Griseb.
				*Sideritis perfoliata* L. (1)	*Stachys recta* L. (0)		(1)
				*Sideritis romana* L. (0) *Sideritis sipylea* Boiss. (0)	*Ajuga reptans* L. (0)		
JCLP_196	σκολοπένδριον	ἄσπληνος,	1		*Asplenium ceterach* L.		
		σκολοπένδριον			(3)		
JCLP_198	σκορπίουρον	ήλιοτρόπιον τò	1		*Heliotropium*		
		μέγα, σκορπίουρον			*europaeum* L. (1)		
JCLP_204	στρύχνον	στρύχνον κηπαĩον	3		*Solanum nigrum* L. (3)	*Solanum melongena* L.	
					*Solanum villosum* Mill. (2)	(2)	
JCLP_210	τετράγκαθον	τραγάκανθα	3		*Astragalus creticus*		*Astragalus sempervirens*
					Lam. (0)		Lam. (1) *Astragalus prusianus* Boiss. (0)
JCLP_214	τρίβολος	τρίβολος: ένυδρος	1		*Trapa natans* L. (0)		
JCLP_215	τριφύλλι	τρίφυλλον	1	*Bituminaria bituminosa *(L.) C.H.Stirt. (0)			
JCLP_217	υοσκύαμος	ύοσκύαμος: άνθη	1		*Hyoscyamus aureus* L.		
JCLP_217	υοσκύαμος	ὑοσκύαμος: ἄνθη ὑποπόρφυρα	1		*Hyoscyamus niger* L. (2)		
JCLP_217	υοσκύαμος	ὑοσκύαμος: τρίτος, ἄνθη λευκά	1	*Hyoscyamus albus* L. (3)			
JCLP_221	φλόμος	φλόμος: λευκή, θηλεία	4	*Verbascum phlomoides* L. (3)	*Verbascum sinuatum* L. (3) *Verbascum thapsus* L. (3)		*Verbascum undulatum* Lam. (0)
JCLP_225	χαμαίδαφνη	χαμαιδάφνη	2		*Ruscus hypophyllum* L. (1)	*Danae racemosa* (L.) Moench (1)	
JCLP_227	χαμαίμιλον	χαμαίμηλον, in	2	*Anthemis chia* L. (0)			
		άνθεμίς		*Matricaria chamomilla* L. (3)			
JCLP_228	χαμαίπιτυς	χαμαίπιτυς: έτέρα	2	*Thymelaea hirsuta* (L.) Endl. (3)		*Ajuga iva* (L.) Schreb. (1)	
JCLP_228	χαμαίπιτυς	χαμαίπιτυς: τρίτη	1		*Ajuga chamaepitys* (L.) Schreb.(1)		
JCLP_228	χαμαίπιτυς	χαμαίπιτυς: χαμαίπιτυς	2		*Ajuga iva* (L.) Schreb. (1)	*Ajuga reptans* L. (3)	
JCLP_231	χελιδονεα	χελιδόνιον μέγα	1		*Chelidonium majus* L. (3)		
JCLP_235	ψύλλεον	ψύλλιον	2	*Plantago afra* L. (1)	*Plantago indica* L. (1)		

**Table columns:** **Lemma tag**: Unique identifier of the lemmatised JC plant name. **JC plant name**: Spelling variants of the respective name also mentioned in JC are not shown. **DMM plant name**: Name in DMM according to Beck (2005) and Wellmann (1907); Possible “types” of DMM plants are listed on separate lines. **# Candidate plants (CPs)**: Number of candidate plants (CPs) for each JC/DMM plant; In case of “types” of DMM plants the number refers to CPs for the respective type. Because the same CP can be associated with different JC/DMM plants or “types” of DMM plants, their sum (141) is greater the number of unique CPs (130). **Strong candidate**: Candidate plant with a total score of ≥5 in the botanical analysis. **Moderate candidate**: Candidate plant with a total score of 3–4 in the botanical analysis. **Weak candidate**: Candidate plant with a total score of <3 in the botanical analysis. **Not contained in FT or FC**: Candidate plant not contained in FT (Davis et al., 1966–85, suppl. 1988 and 2001) or FC (Meikle, 1977, 1985).

**Table 3:** General issues regarding the application of the methodology and specific recommendations as to how to address them.

Step	Topic	Problem	Recommendation	
General	Data Management	This methodology generates numerous data outputs across multiple disciplines and team members, often with complex (one to many) relationships between records in the resulting tables.	Nominate a competent member of the team from start as “data manager” to oversee outputs and ensure integrity and compatibility to facilitate later analysis.	
General	Data compatibility for analysis	Plant names (historical or scientific) are liable to vary in different reports and are not a reliable datum for combining or comparing records between sources and tables.	Implement a comprehensive system of IDs for each major data output from the start (e.g. JC lemma tags at Stage 1, DMM suggested candidate species IDs at Stage 2.1 and unique taxonomic plant IDs at Stage 2.2). Consider devising a relational database to store and assess data as its collected if possible.	
1.1	Translation of the text	Differing meaning of words depending on the temporal context	This step should be overseen by philologists/linguists and, if appropriate, native speakers of the respective language or idiom. Use dictionaries with appropriate temporal context.	
1.1	Coding and interpretation of recipes	Misinterpretation of the information in the text	Define a multidisciplinary team (humanities and sciences) for the interpretation of the recipes.	
1.1–2	Tagging and lemmatising of the words	Application of either a linguistic or a pharmacognostic approach can lead to differing categorisations of words.	Ensure the close collaboration of philologists and pharmacologists throughout the process.	
1.3	Information about plants in recipe texts	Recipe collections usually do not contain plant descriptions or illustrations of plants.	Identify and gain access to culturally and historically associated botany or pharmacognosy texts with pertinent plant descriptions.	
1.3	Cross-referencing plant names	A given historical plant name can be associated with different botanical species over time.	Consider the appropriate temporal context when using lexica or glossaries of plant names.	
1.4	Botanical information in historical texts	Botanical terminology in historical plant descriptions is based on pre-modern understanding of plant anatomy and therefore prone to misinterpretation.	Assign this task to botanists experienced with historical texts. Study the plant descriptions in the original language. Generally, and especially when working with translations, consider different possibilities of interpreting the information. See also point 1.5.	
1.5	Plant uses in historical texts	Medicinal terminology in historical texts is based on pre-modern understanding of human anatomy or health and disease and therefore prone to misinterpretation.	Assign this task to historians of pharmacy/medicine or ethnopharmacologists experienced with historical texts. See also point 1.4.	
1.5	Medicinal use groups	Classifying historical plant uses into categories of modern medical conditions is often difficult.	Use straightforward classification systems based on organ systems or body parts and which facilitate cross-cultural comparisons.	
2.1	Suggested botanical identities of ancient plant names	Reliability of the references used to establish the list of suggested candidate species.	Use culturally and historically appropriate primary literature from botany, pharmacognosy and ethnobotany or ethnopharmacology. If using data from modern field studies, take into consideration potential temporal changes in the plants associated with the respective name.	
2.1	Suggested candidate plants	Relying on suggested candidate plants for the botanical assessment involves the risk of not capturing all possible candidate plants.	Consider the complete flora of the geographical area associated with the text rather than only suggested candidate plants from other sources. This requires the capacity to process large and complex data sets.	
2.2	Taxonomic verification of botanical names	Botanical names reported, especially in older literature, are often incomplete, outdated or ambiguous.	Verify botanical names with Kew’s MPNS or other appropriate and up-to-date databases of plant taxonomy and nomenclature. Be aware that a scientific binomial without its author citation may map to more than one recognised scientific name (“homonyms”), which may relate to entirely different species.	
2.2	Use of different synonyms of botanical names	The sources employed for the investigations in stage 3 often use different synonyms for the same plant and the taxonomic relationships between plant names are liable to shift as taxonomic knowledge improves.	Choose a snapshot date to output a full taxonomy from MPNS (or other appropriate sources) for the suggested candidate species and use the currently accepted names from this, as a primary index of species to be explored. Cross-reference this with a full list of synonyms from the same taxonomy to enable a comprehensive search of relevant literature for each plant.	
3.1	Plant taxonomy books	Depending on the geographical region covered by the plant taxonomy books selected, candidate plants for the herbal *materia medica* imported from neighbouring regions may be missed.	Include also plant taxonomy books from neighbouring regions in the botanical analysis.	
3.1	Full text descriptions as diagnostic tools	Full text descriptions of species as used in this study are a rich source of diagnostic observations, but extracting that data for analysis is arduous and often requires some level of interpretation.	Digital diagnostic descriptive matrices on the other hand (such as those built to support online interactive identification systems) would be a perfect source for this type of information, but such matrices exist for very few plant groups.	
3.2	Categorisation of modern plant uses	Classifying traditional or local plant uses into categories of modern medical conditions is often difficult.	Use straightforward classification systems based on organ systems or body parts and which facilitate cross-cultural comparisons.	
3.2	Plant uses reported in modern resources	Depending on the number and the quality of the resources used, the subsequent comparative medicinal analysis may lead to incomplete results.	Ensure the use of a representative number of appropriate resources. The ethnobotanical studies used should derive from culturally or geographically associated areas and the plants reported should be taxonomically verified.	
3.3	Data from human clinical trials	Because for many plants no information on patient studies is available, the data basis remains patchy.	Combine the data available from human clinical trials with the data from herbal medicinal or ethnobotanical resources as per step 3.2. Treat this data as purely supplementary information.	
4.1–3	Establishing the data matrices	Matching of historical with modern data is a critical step and often requires some level of interpretation.	See below, points 5.1 and 5.2.	
5.1	Comparative botanical analysis	Specific morphological characteristics described in the historical text, may not be highlighted in the floristic works. Consequently, no comparison of the concerned characteristic is possible.	Beside standard floristic works, also include less specialised texts of plant descriptions.	
5.1	Botanical terminology in historical plant descriptions	The terminology used in historical texts to describe a certain feature of a plant or refer to a specific plant part often does not correspond to modern botanical or morphological understanding.	Take into account the differing understanding of plant anatomy or botany in pre-modern societies when matching historical with modern information. Assign this task to a specialist well-acquainted with the flora of the respective region.	
5.2	Comparative medicinal analysis	The comparability of the historical and modern plant uses depends on the appropriateness of the classification system of the use groups selected.	Use a classification system facilitating cross-cultural comparisons (see point 1.5).
5.3	Statistical evaluation	Due to the inherent limitations of available data (sparse or unusable historical data points, difficulties mapping historical data against modern information about corresponding candidate plants, etc.), traditional statistical tests are not possible and analysis and evaluation must necessarily remain broad and open to interpretation.	Our approach seeks to define broad categories of confidence in each candidate plant to help rule out less likely candidates and identify those which warrant further research. This method can be refined through adjustment of the scoring thresholds to better fit available data and expose further nuance between candidate plants or extended through application of additional techniques.

**Footnotes:** **Step**: Step of the methodology in the respective stage (e.g. 5.2 = Stage 5, step 2). **Problem**: Explanation of the specific challenge encountered in processing the respective step of the methodology. **Recommendation**: Recommendation as to how to address the issue and/or improve the respective step of the methodology.

## Data Availability

We have shared the link to our data at the Results section.

## Abbreviations

DMM: Dioscorides’ *De Materia Medica*
JC: Commentary version of John the Physician’s *Therapeutics*
MPNS: Kew’s Medicinal Plant Names Services
FT: Flora of Turkey and the Eastern Aegean islands
FC: Flora of Cyprus
