# Supplementary material for: A systematic methodology to assess the identity of plants in historical texts: A case study based on the Byzantine pharmacy text John the Physician’s Therapeutics
Source: J Ethnopharmacol. Author manuscript; Available in PMC 2024 Mar 25. (PMC7615571; doi:10.1016/j.jep.2023.117622)
Supplement: Document S1 [file EMS193501-supplement-Document_S1.pdf]

Document S1. Reports of suggested botanical identities according to the literature. As per methodology stage 2 step 2.1, for each of the 50 JC plant names a list of the possible botanical identities previously suggested in the literature was compiled. For each JC plant name the results of this step were summarised in separate report.

Reports of suggested botanical identities according to the literature

Methodology Stage 2 – Establishing the list of suggested candidate plants

Step 2.1 – Compiling identities of plants reported in the literature

For each of the 50 JC plant name in our sample, we compiled a list of the possible botanical identities previously suggested in the literature. Information was drawn from:

i) Earlier scholars who studied the flora of Greece and the Eastern Mediterranean and suggested identifications for plants mentioned in DMM (Sibthorp, eds. Smith and Lindley, 1806-1840; Billerbeck, 1824; Fraas, 1845; Lenz, 1859);

ii) Modern ethnobotanical field studies from Greece and Cyprus reporting Greek plant names (Arnold-Apostolides, 1985; Hanlidou et al., 2004; Della et al., 2006; Karousou and Deirmentzoglou, 2011; Lardos, 2016; Axiotis et al., 2018; Tsioutsiou et al., 2019).

Any botanical taxon suggested in the literature was included as a candidate if the respective Greek plant name corresponded to the plant name appearing in DMM.

(Reference list, see last page)

Supplementary Material to the paper

A systematic methodology to assess the identity of plants in historical texts: A case study based on the Byzantine pharmacy text John The Physician's *Therapeutics*

July 2023

Andreas Lardos, Kristina Patmore, Robert Allkin, Rebecca Lazarou, Mark Nesbitt, Andrew C. Scott, Barbara Zipser

Corresponding author:
Andreas Lardos, E-mail address: andreas.lardos@zhaw.ch; Tel.: +41 58 934 50 00

Suggested botanical identity according to the literature

Correspondence between plant name in JC and DMM

Plant name JC (Headword)	Plant name in DMM	Chapter in DMM (Wellmann edition)
αδίαντον	αδίαντον	IV, 134
**Comments**: The names cited in JC and DMM are consistent.		

Information from the literature

a) Botanical texts with reference to the plants in DMM

Plant name in DMM	Chapter in DMM (edition)	Additional names	Plant	Relevant details	Reference
αδίαντον	IV, 136	καλλίτριχον	Adiantum capillus-veneris		Billerbeck, 1824
αδίαντον	IV, 134	πολυτρίχι	Adiantum capillus-veneris L.		Fraas, 1845
αδίαντον	IV, 134	DMM: Polytrichon, Kallitrichon, Trichomanes, Ebenotrichon	Adiantum capillus-veneris L.		Lenz, 1859
αδιαντον	not mentioned		Adiantum capillus-veneris L.		Sibthorp, 1806-40

b) Ethnobotanical field studies

Plant name	Plant	Study region	References
pollitrichi, skorpithi	Adiantum capillus-veneris L.	Cyprus	Arnold-Apostolides, 1985
adiantos, polytrihi	Adiantum capillus-veneris L.	Thessaloniki (herbal markets)	Hanlidou, 2004
--	--	Cyprus (wild food plants)	Della, 2006
--	--	Cyprus (herbal markets)	Karousou, 2011
--	--	Cyprus (monasteries)	Lardos, 2016
--	--	North Aegean islands	Axiotis, 2018
--	--	Central Macedonia	Tsioutsiou, 2019

Reference details: see separate list

Suggested botanical identity according to the literature

Correspondence between plant name in JC and DMM

Plant name JC (Headword)	Plant name in DMM	Chapter in DMM (Wellmann edition)
αγριοσταπίδα, αγριοσταφίδα	σταφίς αγρία	IV, 152
**Comments**: The names cited in JC and DMM are consistent.		

Information from the literature

a) Botanical texts with reference to the plants in DMM

Plant name in DMM	Chapter in DMM (edition)	Additional names	Plant	Relevant details	Reference
σταφίς αγρία	IV, 156	άγρια σαφίδα	Delphinium staphisagria		Billerbeck, 1824
σταφίς αγριά	IV, 153	άγρια σαφίδα	Delphinium staphisagria L.		Fraas, 1845
σταφίς αγριά	IV, 153	άγρια σταφίδα	Delphinium staphisagria L.		Lenz, 1859
σταφις αγρια	IV, 156	άγριο σαφίδα	Delphinium staphisagria L.		Sibthorp 1806-40

b) Ethnobotanical field studies

Plant name	Plant	Study region	References
aghryostaphydha	Delphinium staphisagria L.	Cyprus	Arnold-Apostolides, 1985
papazoto	Delphinium staphisagria L.	Thessaloniki (herbal markets)	Hanlidou, 2004
--	--	Cyprus (wild food plants)	Della, 2006
--	--	Cyprus (herbal markets)	Karousou, 2011
--	--	Cyprus (monasteries)	Lardos, 2016
--	--	North Aegean islands	Axiotis, 2018
--	--	Central Macedonia	Tsioutsiou, 2019

Reference details: see separate list

Suggested botanical identity according to the literature

Correspondence between plant name in JC and DMM

Plant name JC (Headword)	Plant name in DMM	Chapter in DMM (Wellmann edition)
άκανθη άσπρη	άκανθη λευκή	III, 12
**Comments**: The names cited in JC and DMM are consistent.		

Information from the literature

a) Botanical texts with reference to the plants in DMM

Plant name in DMM	Chapter in DMM (edition)	Additional names	Plant	Relevant details	Reference
άκανθη λευκή	not mentioned		Echinops lanuginosa		Billerbeck, 1824
άκανθη λευκή	III, 12	Modern: βουνάγκαδα	Cnicus ferox L.		Fraas, 1845
--	--		--		Lenz, 1859
ακανθη λευκη	not mentioned	Modern: άσπρη αγκάθα	Picnomon acarna		Sibthorp 1806-40
ακανθη λευκη	not mentioned		Echinops graecus		Sibthorp 1806-40

b) Ethnobotanical field studies

Plant name	Plant	Study region	References
--	--	Cyprus	Arnold-Apostolides, 1985
--	--	Thessaloniki (herbal markets)	Hanlidou, 2004
asprangatho	Onopordum bracteatum Boiss. & Heldr.	Cyprus (wild food plants)	Della, 2006
--	--	Cyprus (herbal markets)	Karousou, 2011
ασπράγκαθο	Onopordum bracteatum Boiss.	Cyprus (monasteries)	Lardos, 2016
--	--	North Aegean islands	Axiotis, 2018
--	--	Central Macedonia	Tsioutsiou, 2019

Reference details: see separate list

Suggested botanical identity according to the literature

Correspondence between plant name in JC and DMM

Plant name JC (Headword)	Plant name in DMM	Chapter in DMM (Wellmann edition)
άλυσσος	άλλυσσον	III, 91
**Comments**: The names cited in JC and DMM are consistent. It should be noted, that the άλλυσσος of Galen (de antidot. 2, 168) and the Alysson of Pliny do not seem to be the same plants as the άλλυσσον of Dioscorides. According to Fraas (1845) the plant described by Galen might be *Echium plantagineum* (or another Boraginaceae), while the plant described by Pliny could be *Rubia lucida*. However, both identifications are highly uncertain. It is unknown to which of these types the JC plant refers to.		

Information from the literature

a) Botanical texts with reference to the plants in DMM

Plant name in DMM	Chapter in DMM (edition)	Additional names	Plant	Relevant details	Reference
άλλυσσον	III, 105		Scutellaria galericulata	Fraas disapproves this identification	Billerbeck, 1824
άλλυσσον	III, 95		Farsetia clypeata Br.		Fraas, 1845
άλλυσσον	III, 95		Alyssum clypeatum L.		Lenz, 1859
άλλυσσον	III, 105		Alyssum alpestre L.		Sibthorp 1806-40
άλλυσσον	III, 105		Fibigia clypeata	Identity stated as uncertain	Sibthorp 1806-40

b) Ethnobotanical field studies

Plant name	Plant	Study region	References
--	--	Cyprus	Arnold-Apostolides, 1985
--	--	Thessaloniki (herbal markets)	Hanlidou, 2004
--	--	Cyprus (wild food plants)	Della, 2006
--	--	Cyprus (herbal markets)	Karousou, 2011
--	--	Cyprus (monasteries)	Lardos, 2016
--	--	North Aegean islands	Axiotis, 2018
--	--	Central Macedonia	Tsioutsiou, 2019

Reference details: see separate list

Suggested botanical identity according to the literature

Correspondence between plant name in JC and DMM

Plant name JC (Headword)	Plant name in DMM	Chapter in DMM (Wellmann edition)
αλόη	αλόη	III, 22
**Comments**: The names cited in JC and DMM are consistent.		

Information from the literature

a) Botanical texts with reference to the plants in DMM

Plant name in DMM	Chapter in DMM (edition)	Additional names	Plant	Relevant details	Reference
αλόη	III, 22		Aloe perfoliata, syn. Aloe vulgaris		Billerbeck, 1824
αλόη	III, 22		Aloe perfoliata		Fraas, 1845
αλόη	III, 22		Aloe L.		Lenz, 1859
αλοη	not mentioned	αλοε	Aloe vulgaris Bauh.		Sibthorp 1806-40

b) Ethnobotanical field studies

Plant name	Plant	Study region	References
allàs	Aloe vera (L.) Burm.f.	Cyprus	Arnold-Apostolides, 1985
aloi	Aloe vera (L.) Burm.fil.	Thessaloniki (herbal markets)	Hanlidou, 2004
--	--	Cyprus (wild food plants)	Della, 2006
aloi	Aloe vera (L.) Burm.fil.	Cyprus (herbal markets)	Karousou, 2011
αλόι	Aloe vera (L.) Burm.f.	Cyprus (monasteries)	Lardos, 2016
--	--	North Aegean islands	Axiotis, 2018
--	--	Central Macedonia	Tsioutsiou, 2019

Reference details: see separate list

Suggested botanical identity according to the literature

Correspondence between plant name in JC and DMM

Plant name JC (Headword)	Plant name in DMM	Chapter in DMM (Wellmann edition)
αμάραντον	ελίχρυσος, χρυσάνθεμος, αμάραντος	IV, 57
**Comments**: The names cited in JC is consistent with one of the alternative names cited in DMM.		

Information from the literature

a) Botanical texts with reference to the plants in DMM

Plant name in DMM	Chapter in DMM (edition)	Additional names	Plant	Relevant details	Reference
αμάραντον	IV, 57	DMM: ελίχρυσος, χρυσ-άνθεμος; Modern: καλοκοιμιθίκος, CY: δάκρυα της Παναγιάς	Gnaphalium stoechas		Billerbeck, 1824
			Tanacetum annuum L.	acc. to Schneider	Billerbeck, 1824
			Gnaphalium orientale	acc. to Sprengel	Billerbeck, 1824
αμάρανθος	IV, 57	DMM: ελίχρυσος, χρυσ-άνθεμος; Modern: καλοκοιμιθίκος, CY: δάκρυα της Παναγιάς	Gnaphalium stoechas		Fraas, 1845
			Tanacetum annuum L.	acc. to Sprengel; however, Fraas claims the plant was not found in Greece	Fraas, 1845
αμάρανθον	IV, 57	DMM: ελίχρυσον, χρυσάνθεμον; Modern: αμάρανθον	Gnaphalium stoechas L.		Lenz, 1859
αειζον	not mentioned	αμάραντο	Sedum eriocarpum		Sibthorp 1806-40
αειζον	not mentioned	αμάραντο	Sedum ochroleucum		Sibthorp 1806-40
πολιον	III.124	αμάραντο	Teucrium polium L.		Sibthorp 1806-40

b) Ethnobotanical field studies

Plant name	Plant	Study region	References
athanaton	Helichrysum conglobatum (Viv.) Steudel	Cyprus	Arnold-Apostolides, 1985
athanaton	Helichrysum italicum (Roth) Don	Cyprus	Arnold-Apostolides, 1985
--	--	Thessaloniki (herbal markets)	Hanlidou, 2004
--	--	Cyprus (wild food plants)	Della, 2006
--	--	Cyprus (herbal markets)	Karousou, 2011
--	--	Cyprus (monasteries)	Lardos, 2016
αμάραντο	Helichrysum stoechas DC.	North Aegean islands	Axiotis, 2018
--	--	Central Macedonia	Tsioutsiou, 2019

Reference details: see separate list

Suggested botanical identity according to the literature

Correspondence between plant name in JC and DMM

Plant name JC (Headword)	Plant name in DMM	Chapter in DMM (Wellmann edition)
άμπελος	άμπελος οινόφορος	V, 1
	άπμελος αγρία	V, 2; IV, 182
**Comments**: The names cited in JC and DMM are basically consistent. It can be assumed that the plant referred to in JC corresponds to άμπελος οινόφορος, the cultivated type of DMM.		

Information from the literature

a) Botanical texts with reference to the plants in DMM

Plant name in DMM	Chapter in DMM (edition)	Additional names	Plant	Relevant details	Reference
άπμελος αγρία	V	αγριάμπελος	Vitis sativa		Billerbeck, 1824
άπμελος αγρία	IV, 183	αγριομπέλι	Clematis vitalba		Billerbeck, 1824
άμπελος οινόφορος	V	αμπελόνα, κλήμα	Vitis sylvestris		Billerbeck, 1824
άμπελος οινόφορος	V, 1	κλήμα	Vitis vinifera L.		Fraas, 1845
--	--	Theophr.: άμπελος	Vitis vinifera L.	cultivated grape vine	Lenz, 1859
--	--	Theophr.: άμπελος αγρία	Vitis vinifera L.	wild grape vine	Lenz, 1859
άμπελος αγρία	IV, 180		Tamus communis L.		Lenz, 1859
αμπελος αγρια	V, 2	αμπελόνα	Vitis vinifera L.		Sibthorp 1806-40
αμπελος αγρια	Not stated	αβρυά	Tamus communis L.		Sibthorp 1806-40
αμπελος οινοφορος	V, 2	αμπελόνα	Vitis vinifera L.		Sibthorp 1806-40

b) Ethnobotanical field studies

Plant name	Plant	Study region	References
ambeli, ambelos i oinofora	Vitis vinifera L.	Cyprus	Arnold-Apostolides, 1985
ambellos i lefki tou Dhioskoridhi	Tamus communis L.	Cyprus	Arnold-Apostolides, 1985
ambeli	Vitis vinifera L. ssp. vinifera	Thessaloniki (herbal markets)	Hanlidou, 2004
--	--	Cyprus (wild food plants)	Della, 2006

--	--	Cyprus (herbal markets)	Karousou, 2011
άμπελος, αμπέλι	Vitis vinifera L.	Cyprus (monasteries)	Lardos, 2016
αμπέλι	Vitis vinifera L. ssp. sylvestris (C.C.Gmel.)	North Aegean islands	Axiotis, 2018
--	--	Central Macedonia	Tsioutsiou, 2019

Reference details: see separate list

Suggested botanical identity according to the literature

Correspondence between plant name in JC and DMM

Plant name JC (Headword)	Plant name in DMM	Chapter in DMM (Wellmann edition)
αριστολοχία	αριστολοχεία	III, 4 (2 types)
**Comments**: The names cited in JC and DMM are consistent.		

Information from the literature

a) Botanical texts with reference to the plants in DMM

Plant name in DMM	Chapter in DMM (edition)	Additional names	Plant	Relevant details	Reference
αριστολοχία στρογγύλη	III, 4		Aristolochia rotunda		Billerbeck, 1824
αριστολοχία μακρά	III, 4		Aristolochia longa		Billerbeck, 1824
αριστολοχία κληματίτις	III, 4		Aristolochia clematitis		Billerbeck, 1824
αριστολοχία στρογγύλη	III, 4	Modern: πικρουνιά	Aristolochia pallida W.		Fraas, 1845
αριστολοχία μακρά	III, 4	Modern: πικρόρριζα	Aristolochia parvifolia Sibth.		Fraas, 1845
αριστολοχία κληματίτις	III, 4		Aristolochia boetica L.		Fraas, 1845
αριστολοχία	III, 4-6	Modern: πικρουνιά	Aristolochia pallida Willdeonow	in Greece	Lenz, 1859
αριστολοχία δακτυλίτις	III, 4-6	Modern: πικρόρριζα	Aristolochia parviflora Sibthorp	in Greece	Lenz, 1859
αριστολοχία κληματίτις	III, 4-6		Aristolochia bätica L.	in Greece	Lenz, 1859
αριστολοχία	III, 4-6		Aristolochia rotunda Matth.	in Italy	Lenz, 1859
αριστολοχία	III, 4-6		Aristolochia parviflora Sibthorp	in Italy	Lenz, 1859
αριστολοχια στρογγυλη	not mentioned		Aristolochia pallida Willd.		Sibthorp 1806-40
αριστολοχια μακρα	not mentioned	Modern: πικρόρριζα	Aristolochia parvifolia		Sibthorp 1806-40
αριστολοχια κληματιτις	not mentioned		Aristolochia baetica L.		Sibthorp 1806-40

b) Ethnobotanical field studies

Plant name	Plant	Study region	References
aristolochia	Aristolochia sempervirens L.	Cyprus	Arnold-Apostolides, 1985
aristolochia	Aristolochia parvifolia Sm.	Cyprus	Arnold-Apostolides, 1985
--	--	Thessaloniki (herbal markets)	Hanlidou, 2004
--	--	Cyprus (wild food plants)	Della, 2006
--	--	Cyprus (herbal markets)	Karousou, 2011
--	--	Cyprus (monasteries)	Lardos, 2016
--	--	North Aegean islands	Axiotis, 2018
--	--	Central Macedonia	Tsioutsiou, 2019

Reference details: see separate list

Suggested botanical identity according to the literature

Correspondence between plant name in JC and DMM

Plant name JC (Headword)	Plant name in DMM	Chapter in DMM (Wellmann edition)
αρνόγλωσσον	αρνόγλωσσον	II, 126
**Comments**: The names cited in JC and DMM are consistent.		

Information from the literature

a) Botanical texts with reference to the plants in DMM

Plant name in DMM	Chapter in DMM (edition)	Additional names	Plant	Relevant details	Reference
αρνόγλωσσον	II, 153	πολύνευρον, πεντάνευρον	Plantago major		Billerbeck, 1824
αρνόγλωσσον επταπλευρόν	II, 153		Plantago media		Billerbeck, 1824
αρνόγλωσσον μικρόν	II, 153		Plantago lagopus		Billerbeck, 1824
αρνόγλωσσον μείζον	II, 152	πεντάνευρον	Plantago asiatica L.		Fraas, 1845
αρνόγλωσσον μικρόν	II, 152		Plantago lagopus L.		Fraas, 1845
αρνόγλωσσον (grössere)	II, 152	πεντάνευρον	Plantago asiatica L.		Lenz, 1859
αρνόγλωσσον (kleinere)	II, 152		Plantago lagopus L.		Lenz, 1859
αρνογλωσσον	Not stated	πεντάνευρον	Plantago major L.		Sibthorp 1806-40
αρνογλωσσον	Not stated		Plantago altissima Lois.		Sibthorp 1806-40
αρνογλωσσον μικρον	Not stated		Plantago lagopus L.		Sibthorp 1806-40
αρνογλωσσον μικρον	Not stated		Plantago maritima L.		Sibthorp 1806-40
αρνογλωσσον μικρον	Not stated	πεντάνευρον	Plantago lanceolata L.		Sibthorp 1806-40
αρνογλωσσον μικρον	Not stated		Plantago spp.		Sibthorp 1806-40

b) Ethnobotanical field studies

Plant name	Plant	Study region	References
arnoghlosso	Plantago lagopus L.	Cyprus	Arnold-Apostolides, 1985
arnoghlosson, pendanevron	Plantago major L.	Cyprus	Arnold-Apostolides, 1985

arnoghlosso, pendanevro	Plantago lancelolata L.	Cyprus	Arnold-Apostolides, 1985
pentanevro	Plantago major L.	Thessaloniki (herbal markets)	Hanlidou, 2004
--	--	Cyprus (wild food plants)	Della, 2006
pentanevro	Plantago major L.	Cyprus (herbal markets)	Karousou, 2011
pentanevro, fkia tou laghou	Plantago lanceolata L.	Cyprus (monasteries)	Lardos, 2016
pentanevro	Plantago major L.	Cyprus (monasteries)	Lardos, 2016
--	--	North Aegean islands	Axiotis, 2018
pentanevro	Plantago media L.	Central Macedonia	Tsioutsiou, 2019

Reference details: see separate list

Suggested botanical identity according to the literature

Correspondence between plant name in JC and DMM

Plant name JC (Headword)	Plant name in DMM	Chapter in DMM (Wellmann edition)
άσαρ	άσαρον	I, 10
**Comments**: The names cited in JC and DMM are consistent.		

Information from the literature

a) Botanical texts with reference to the plants in DMM

Plant name in DMM	Chapter in DMM (edition)	Additional names	Plant	Relevant details	Reference
άσαρον	I, 9	άσαρον, νάρδος αγρία	Asarum europaeum		Billerbeck, 1824
άσαρον	I, 9	άσαρον	Asarum europaeum L.		Fraas, 1845
άσαρον	I, 9	άσαρον, νάρδος αγρία	Asarum europaeum		Lenz, 1859
ασαρον	Not stated	ασάρον	Asarum europaeum L.		Sibthorp 1806-40

b) Ethnobotanical field studies

Plant name	Plant	Study region	References
--	--	Cyprus	Arnold-Apostolides, 1985
--	--	Thessaloniki (herbal markets)	Hanlidou, 2004
--	--	Cyprus (wild food plants)	Della, 2006
--	--	Cyprus (herbal markets)	Karousou, 2011
--	--	Cyprus (monasteries)	Lardos, 2016
--	--	North Aegean islands	Axiotis, 2018
--	--	Central Macedonia	Tsioutsiou, 2019

Reference details: see separate list

Suggested botanical identity according to the literature

Correspondence between plant name in JC and DMM

Plant name JC (Headword)	Plant name in DMM	Chapter in DMM (Wellmann edition)
ασφόδελος	ασφόδελος	II, 169
**Comments**: The names cited in JC and DMM are consistent.		

Information from the literature

a) Botanical texts on the plants in DMM

Plant name in DMM	Chapter in DMM (edition)	Additional names	Plant	Relevant details	Reference
ασφόδελος	II, 199		Asphodelus ramosus		Billerbeck, 1824
ασφόδελος	II, 199	σφερδουλάκα, σπούρδακλα	Asphodelus ramosus L.		Fraas, 1845
ασφόδελος	II, 199	σφερδουλάκα, σπούρδακλα	Asphodelus ramosus L.		Lenz, 1859

b) Ethnobotanical field studies

Plant name	Plant	Study region	References
asphondilos, spourtoulla, etc.	Asphodelus aestivus Brot.,	Cyprus	Arnold-Apostolides, 1985
asphondilos, spourtoulla, etc.	Asphodelus fistulosus L.	Cyprus	Arnold-Apostolides, 1985
--	--	Thessaloniki (herbal markets)	Hanlidou, 2004
--	--	Cyprus (wild food plants)	Della, 2006
--	--	Cyprus (herbal markets)	Karousou, 2011
σπούρτουλλος, αρκόσιελλος, ασφόδελος	Asphodelus aestivus Brot.	Cyprus (monasteries)	Lardos, 2016
ασπούρδουλας	Asphodelus aestivus Brot.	North Aegean islands	Axiotis, 2018
--	--	Central Macedonia	Tsioutsiou, 2019

Reference details: see separate list

Suggested botanical identity according to the literature

Correspondence between plant name in JC and DMM

Plant name JC (Headword)	Plant name in DMM	Chapter in DMM (Wellmann edition)
βετονίκη	βεττονίκη, see κέστρον	IV, 1
**Comments**: The names cited in JC and DMM are consistent.		

Information from the literature

a) Botanical texts with reference to the plants in DMM

Plant name in DMM	Chapter in DMM (edition)	Additional names	Plant	Relevant details	Reference
βεττονίκη	IV, 1	DMM: κέστρον	Betonica alopecurus		Billerbeck, 1824
βεττονίκη	IV, 1	DMM: κέστρον; Modern: βετόνικα, βαμβακίτσα, τζάϊ ελληνικόν	Sideritis syriaca L.	fragrant and found in cool places (see DMM)	Fraas, 1845
βεττονίκη	IV, 1	DMM: κέστρον; Modern: βετόνικα, βαμβακίτσα, τζάϊ ελληνικόν	Sideritis cretica L.	fragrant and found in cool places (see DMM)	Fraas, 1845
βεττονίκη	IV, 1	DMM: κέστρον	Betonica alopecurus	not fragrant	Fraas, 1845
--	--	--	--	--	Lenz, 1859
κέστρον		Modern: βετονική	Stachys alopercuros		Sibthorp 1806-40

b) Ethnobotanical field studies

Plant name	Plant	Study region	References
--	--	Cyprus	Arnold-Apostolides, 1985
--	--	Thessaloniki (herbal markets)	Hanlidou, 2004
--	--	Cyprus (wild food plants)	Della, 2006
--	--	Cyprus (herbal markets)	Karousou, 2011
--	--	Cyprus (monasteries)	Lardos, 2016
--	--	North Aegean islands	Axiotis, 2018
--	--	Central Macedonia	Tsioutsiou, 2019

Reference details: see separate list

Suggested botanical identity according to the literature

Correspondence between plant name in JC and DMM

Plant name JC (Headword)	Plant name in DMM	Chapter in DMM (Wellmann edition)
βρυωνία	βρυωνία, see άμπελος λευκή / μέλαινα	IV, 182 / 183
**Comments**: The name cited in JC corresponds to one of the synonyms mentioned in DMM for άμπελος λευκή (IV, 182) and άμπελος μέλαινα (IV, 183). It remains unknown to which of the two types the JC plant refers to.		

Information from the literature

a) Botanical texts with reference to the plants in DMM

Plant name in DMM	Chapter in DMM (edition)	Additional names	Plant	Relevant details	Reference
άμπελος λευκή	IV, 184	Roman authors: βρυωνιάς, βρυωνίς	Bryonia alba		Billerbeck, 1824
not mentioned	IV, 185	Roman authors: βρυωνίς μέλαινα	Bryonia dioica	Identity stated as uncertain	Billerbeck, 1824
not mentioned	IV, 185	Pliny: bryonia, ampelos agria	Tamus communis	Identity stated as uncertain	Billerbeck, 1824
βρυωνία	IV, 181	DMM: άμπελος λευκή;<br>Modern: άγρια κολοκυθιά, αγριόκλημα	Bryonia cretica L.		Fraas, 1845
βρυωνία μέλαινα	IV, 182	DMM: άμπελος μέλαινα	Bryonia alba L.		Fraas, 1845
βρυωνία	IV, 180	DMM: άμπελος λευκή	Bryonia dioica L.		Lenz, 1859
άμπελος μέλαινα	IV, 181	Modern: αγριόκλημα	Bryonia alba L.		Lenz, 1859
--	--	Modern: άγρια κολοκυθιά, αγριόκλημα	Bryonia cretica L.		Lenz, 1859
άμπελος μέλαινα	not stated		Bryonia alba L.		Sibthorp 1806-40
άμπελος λευκή	not stated	Modern: άγρια κολοκυθιά, αγριόκλημα	Bryonia cretica L.		Sibthorp 1806-40
άμπελος λευκή	not stated	Modern: άγρια κολοκυθιά, αγριόκλημα	Bryonia dioica L.		Sibthorp 1806-40

b) Ethnobotanical field studies

Plant name	Plant	Study region	References
ambelouridha, psevdhovryonia i kritiki	Bryonia cretica L.	Cyprus	Arnold-Apostolides, 1985
vryoniya, avronya, vryonia i kini	Tamus communis L.	Cyprus	Arnold-Apostolides, 1985

--	--	Thessaloniki (herbal markets)	Hanlidou, 2004
--	--	Cyprus (wild food plants)	Della, 2006
--	--	Cyprus (herbal markets)	Karousou, 2011
--	--	Cyprus (monasteries)	Lardos, 2016
--	--	North Aegean islands	Axiotis, 2018
--	--	Central Macedonia	Tsioutsiou, 2019

Reference details: see separate list

Suggested botanical identity according to the literature

Correspondence between plant name in JC and DMM

Plant name JC (Headword)	Plant name in DMM	Chapter in DMM (Wellmann edition)
δαυκίν (*να γενηται εις αλλοτριον τοπον χωρις ινα το σπειρωσιν του το σπερμα)	σταφυλίνος: άγριος / κηπαίος	III, 52 (2 types)
	δαύκος: Κρητικός, έτερος, τρίτος	III, 72 (3 types)
**Comments**: The name cited in JC seems to refer to wild growing varieties of the carrot. This can be concluded based on the additional information cited in JC (*). The name δαυκίν is a modern synonym of σταφύλινος, as suggested in the below references.		

Information from the literature

a) Botanical texts with reference to the plants in DMM

Plant name in DMM	Chapter in DMM (edition)	Additional names	Plant	Relevant details	Reference
σταφυλίνος	III, 59		Daucus carota		Billerbeck, 1824
σταφυλίνος άγριος	III, 59		Daucus guttatus		Billerbeck, 1824
--	--	Theophr. (h. pl. 4,15): δαύκος	Pastinaca lucida L.		Billerbeck, 1824
σταφυλίνος άγριος	III, 52	Modern: αγρία δαυκιά, καρόττα	Daucus carotta		Fraas, 1845
σταφυλίνος άγριος	III, 52	Modern: αγρία δαυκιά, καρόττα	Daucus guttatus Sibth.		Fraas, 1845
σταφυλίνος άγριος	III, 52	Modern: δαυκιά, καρόττα	Daucus carota L.		Lenz, 1859
σταφυλινος	not mentioned	Modern: σταφυλόνα	Daucus carota L.		Sibthorp 1806-40
σταφυλινος αγριος	III, 83		Daucus guttatus		Sibthorp 1806-40
δαυκος	not mentioned		Athamanta cretensis		Sibthorp 1806-40
δαυκος ετερος	not mentioned		Athamanta cervaria		Sibthorp 1806-40
δαυκος τριτος	not mentioned		Seseli ammoides		Sibthorp 1806-40

b) Ethnobotanical field studies

Plant name	Plant	Study region	References
caroto	Daucus carota L.	Cyprus	Arnold-Apostolides, 1985
--	--	Thessaloniki (herbal markets)	Hanlidou, 2004

--	--	Cyprus (wild food plants)	Della, 2006
--	--	Cyprus (herbal markets)	Karousou, 2011
καρόττο, δαυκίν	Daucus carota L.	Cyprus (monasteries)	Lardos, 2016
--	--	North Aegean islands	Axiotis, 2018
--	--	Central Macedonia	Tsioutsiou, 2019

Reference details: see separate list

Suggested botanical identity according to the literature

Correspondence between plant name in JC and DMM

Plant name JC (Headword)	Plant name in DMM	Chapter in DMM (Wellmann edition)
δρακοντία	δρακόντιον, δρακοντία μεγάλη	II, 166
δρακοντία	άρον, δρακοντία μικρά	II, 167
**Comments**: The names cited in JC and DMM are basically consistent. In consideration that the name δρακοντία has two types (μεγάλη and μικρά), both types should be taken into consideration. Another related DMM plant is αρίσαρον II, 168.		

Information from the literature

a) Botanical texts with reference to the plants in DMM

Plant name in DMM	Chapter in DMM (edition)	Additional names	Plant	Relevant details	Reference
δρακόντιον	II, 196	DMM: δρακόντιον μέγα	Arum dracunculus		Billerbeck, 1824
δρακοντία μεγάλη	II, 195	δρακοντίον (in Hippocrates)	Dracunuclus polyphyllus Tournef.		Fraas, 1845
δρακοντία	II, 195		Arum dracunculus L.		Lenz, 1859
δρακόντιον, δρακοντια μεγαλη	not mentioned	δρακοντιά	Arum dracunculus L.		Sibthorp 1806-40
δρακοντια μιρκα	not mentioned		Arum italicum		Sibthorp 1806-40
not mentioned	not mentioned	δρακοντιά	Arum maculatum		Sibthorp 1806-40
αρίσαρον	not mentioned	δρακοντιά	Arisarum vulgare L.		Sibthorp 1806-40

b) Ethnobotanical field studies

Plant name	Plant	Study region	References
dhrakondhya, arkokolokassi	Arum dioscoridis Sm.	Cyprus	Arnold-Apostolides, 1985
dhrakondia	Arum italicum Miller	Cyprus	Arnold-Apostolides, 1985
--	--	Thessaloniki (herbal markets)	Hanlidou, 2004
--	--	Cyprus (wild food plants)	Della, 2006
--	--	Cyprus (herbal markets)	Karousou, 2011
--	--	Cyprus (monasteries)	Lardos, 2016

--	--	North Aegean islands	Axiotis, 2018
drakontia	Arum italicum Mill.	Central Macedonia	Tsioutsiou, 2019
drakontia	Arum maculatum L.	Central Macedonia	Tsioutsiou, 2019

Reference details: see separate list

Suggested botanical identity according to the literature

Correspondence between plant name in JC and DMM

Plant name JC (Headword)	Plant name in DMM	Chapter in DMM (Wellmann edition)
ευπατώριος	ευπατώριος	IV, 41
**Comments**: The names cited in JC and DMM are consistent.		

Information from the literature

a) Botanical texts with reference to the plants in DMM

Plant name in DMM	Chapter in DMM (edition)	Additional names	Plant	Relevant details	Reference
ευπατώριον	IV, 41		Agrimonia eupatoria		Billerbeck, 1824
ευπατώριον	IV, 41	Modern: φονόχορτον	Agrimonia eupatoria L.		Fraas, 1845
ευπατώριον	IV, 41	Modern: φονόχορτον	Agrimonia eupatoria L.		Lenz, 1859
ευπατώριον		Modern: φονόχορτον	Agrimonia eupatoria L.		Sibthorp 1806-40

b) Ethnobotanical field studies

Plant name	Plant	Study region	References
--	--	Cyprus	Arnold-Apostolides, 1985
agrimonio	Agrimonia eupatoria L.	Thessaloniki (herbal markets)	Hanlidou, 2004
--	--	Cyprus (wild food plants)	Della, 2006
--	--	Cyprus (herbal markets)	Karousou, 2011
--	--	Cyprus (monasteries)	Lardos, 2016
αγριμόνια η ευπατόριος	Agrimonia eupatoria L.	North Aegean islands	Axiotis, 2018
--	--	Central Macedonia	Tsioutsiou, 2019

Reference details: see separate list

Suggested botanical identity according to the literature

Correspondence between plant name in JC and DMM

Plant name JC (Headword)	Plant name in DMM	Chapter in DMM (Wellmann edition)
γεντιανή	γεντιανή	III, 3
**Comments**: The names cited in JC and DMM are consistent.		

Information from the literature

a) Botanical texts with reference to the plants in DMM

Plant name in DMM	Chapter in DMM (edition)	Additional names	Plant	Relevant details	Reference
γεντιανή	III, 3		Gentiana lutea L		Billerbeck, 1824
γεντιανή	III, 3		Gentiana lutea L		Fraas, 1845
γεντιανή	III, 3		Gentiana lutea L.		Lenz, 1859
γεντιανη	not mentioned		Gentiana lutea L.		Sibthorp 1806-40

b) Ethnobotanical field studies

Plant name	Plant	Study region	References
--	--	Cyprus	Arnold-Apostolides, 1985
gentiani, agriokapnos	Gentiana lutea L.	Thessaloniki (herbal markets)	Hanlidou, 2004
--	--	Cyprus (wild food plants)	Della, 2006
--	--	Cyprus (herbal markets)	Karousou, 2011
--	--	Cyprus (monasteries)	Lardos, 2016
--	--	North Aegean islands	Axiotis, 2018
--	--	Central Macedonia	Tsioutsiou, 2019

Reference details: see separate list

Suggested botanical identity according to the literature

Correspondence between plant name in JC and DMM

Plant name JC (Headword)	Plant name in DMM	Chapter in DMM (Wellmann edition)
γλυκόριζον	γλυκύρριζα	III, 5
**Comments**: The names cited in JC and DMM are consistent.		

Information from the literature

a) Botanical texts with reference to the plants in DMM

Plant name in DMM	Chapter in DMM (edition)	Additional names	Plant	Relevant details	Reference
γλυκύρριζα	III, 7	γλυκόριζον	Glycyrrhiza echinata		Billerbeck, 1824
γλυκύρριζα	III, 7	γλυκόριζα, ρεγολίτζα	Glycyrrhiza glabra		Billerbeck, 1824
γλυκύρριζα	III, 5		Glycyrrhiza glandulifera L.		Fraas, 1845
γλυκύρριζα	III, 5	γλυκόριζα, ρεγολίτζα	Glycyrrhiza glabra L.		Lenz, 1859
γλυκύρριζα	III, 5		Glycyrrhiza echinata L.		Lenz, 1859
γλυκύρριζα	III, 5		Glycyrrhiza glandulifera Kit.		Lenz, 1859
γλυκυρριζα	Not stated	γλυκόριζα	Glycyrrhiza echinata L.		Sibthorp 1806-40
γλυκυρριζα	Not stated		Glycyrrhiza glandulifera		Sibthorp 1806-40
Not stated	Not stated	γλυκόριζα, ρεγολίτζα	Glycyrrhiza glabra L.		Sibthorp 1806-40

b) Ethnobotanical field studies

Plant name	Plant	Study region	References
ghlikirrhiza	Glycyrrhiza glabra L.	Cyprus	Arnold-Apostolides, 1985
--	--	Thessaloniki (herbal markets)	Hanlidou, 2004
--	--	Cyprus (wild food plants)	Della, 2006
glykoriza, piambali	Glycyrrhiza glabra L.	Cyprus (herbal markets)	Karousou, 2011
--	--	Cyprus (monasteries)	Lardos, 2016
γλυκόρριζα	Glycyrrhiza glabra Torr.	North Aegean islands	Axiotis, 2018

--	--	Central Macedonia	Tsioutsiou, 2019

Reference details: see separate list

Suggested botanical identity according to the literature

Correspondence between plant name in JC and DMM

Plant name JC (Headword)	Plant name in DMM	Chapter in DMM (Wellmann edition)
υοσκύαμος	υοσκύαμος	IV, 68
**Comments**: The names cited in JC and DMM are consistent.		

Information from the literature

a) Botanical texts with reference to the plants in DMM

Plant name in DMM	Chapter in DMM (edition)	Additional names	Plant	Relevant details	Reference
υοσκύαμος μέλας	IV, 69		Hyoscyamus niger		Billerbeck, 1824
υοσκύαμος λευκός	IV, 69	Modern: υοσκύαμος, γερούλι	Hyoscyamus albus		Billerbeck, 1824
υοσκύαμος μηλοειδής	IV, 69		Hyoscyamus aureus		Billerbeck, 1824
υοσκύαμος μέλας	IV, 64		Hyoscyamus niger L.		Fraas, 1845
υοσκύαμος λευκός	IV, 64		Hyoscyamus albus L.		Fraas, 1845
υοσκύαμος μηλοειδής	IV, 64		Hyoscyamus aureus L.		Fraas, 1845
υοσκύαμος (niger)	IV, 64	Modern: γέρως	Hyoscyamus niger L.		Lenz, 1859
υοσκύαμος (albus)	IV, 64		Hyoscyamus albus L.		Lenz, 1859
υοσκύαμος (aureus)	IV, 64		Hyoscyamus aureus L.		Lenz, 1859
υοσκύαμος μέλας	IV, 69		Hyoscyamus niger L.		Sibthorp 1806-40
υοσκύαμος λευκός	IV, 69	Modern: υοσκύαμος, γερούλι	Hyoscyamus albus L.		Sibthorp 1806-40
υοσκύαμος μηλοειδής	IV, 69		Hyoscyamus aureus L.		Sibthorp 1806-40

b) Ethnobotanical field studies

Plant name	Plant	Study region	References
arkokapnos, dontohorton, yeros	Hyoscyamus albus L.	Cyprus	Arnold-Apostolides, 1985
arkokapnos, dontohorton, yeros	Hyoscyamus aureus L.	Cyprus	Arnold-Apostolides, 1985
--	--	Thessaloniki (herbal markets)	Hanlidou, 2004

--	--	Cyprus (wild food plants)	Della, 2006
--	--	Cyprus (herbal markets)	Karousou, 2011
--	--	Cyprus (monasteries)	Lardos, 2016
υοσκύαμος	Hyoscyamus albus L.	North Aegean islands	Axiotis, 2018
--	--	Central Macedonia	Tsioutsiou, 2019

Reference details: see separate list

Suggested botanical identity according to the literature

Correspondence between plant name in JC and DMM

Plant name JC (Headword)	Plant name in DMM	Chapter in DMM (Wellmann edition)
ίππουρις	ίππουρις	IV, 46
**Comments**: The names cited in JC and DMM are consistent.		

Information from the literature

a) Botanical texts with reference to the plants in DMM

Plant name in DMM	Chapter in DMM (edition)	Additional names	Plant	Relevant details	Reference
ίππουρις	IV, 46		Equisetum sylvaticum		Billerbeck, 1824
ίππουρις	IV, 46		Equisetum arvense		Billerbeck, 1824
ίππουρις	IV, 46		Ephedra fragilis L. var. graeca		Fraas, 1845
ίππουρις έτερα	IV, 47	πολυρριχιά	Equisetum limosum L.		Fraas, 1845
--	--	Geopon.: ίππουρις	Equisetum fluviatile L.		Fraas, 1845
ίππουρις	IV, 46		Ephedra fragilis L.		Lenz, 1859
ίππουρις	IV, 46		Equisetum spp.		Lenz, 1859
ιππουρις	Not stated	πολυτρίχι	Equisetum fluviatile L.		Sibthorp 1806-40
ιππουρις ετερα	Not stated		Equisetum limosum L.		Sibthorp 1806-40

b) Ethnobotanical field studies

Plant name	Plant	Study region	References
polykombi, polytrihi	Ephedra fragilis Desf. ssp. campylopoda (C.A.Meyer) Ascherson & Graebner	Cyprus	Arnold-Apostolides, 1985
polykombi	Ephedra major Host	Cyprus	Arnold-Apostolides, 1985
polykombi, ippouris	Equisetum ramosissimum Desf.	Cyprus	Arnold-Apostolides, 1985
polykombi	Equisetum telmateia Ehrh.	Cyprus	Arnold-Apostolides, 1985
ippouris, polykobi	Equisetum spp.	Thessaloniki (herbal markets)	Hanlidou, 2004
--	--	Cyprus (wild food plants)	Della, 2006

--	--	Cyprus (herbal markets)	Karousou, 2011
--	--	Cyprus (monasteries)	Lardos, 2016
--	--	North Aegean islands	Axiotis, 2018
polikombi, ura tu alogu	Equisetum arvense L.	Central Macedonia	Tsioutsiou, 2019

Reference details: see separate list

Suggested botanical identity according to the literature

Correspondence between plant name in JC and DMM

Plant name JC (Headword)	Plant name in DMM	Chapter in DMM (Wellmann edition)
ίρις	ίρις	I, 1
**Comments**: The names cited in JC and DMM are consistent. In DMM three types are mentioned: Ιλλυρική, Μακεδονική, Λιβυκή. It is unknown to which types the JC plant refers to.		

Information from the literature

a) Botanical texts with reference to the plants in DMM

Plant name in DMM	Chapter in DMM (edition)	Additional names	Plant	Relevant details	Reference
ίρις	I, 1		Iris germanica		Billerbeck, 1824
ίρις	I, 1	Modern: κρίνος	Iris germanica L.		Fraas, 1845
ίρις	I, 1	Modern: κρίνος	Iris germanica L.		Lenz, 1859
ίρις	not stated	Modern: κρίνος	Iris germanica L.		Sibthorp 1806-40
ίρις	not stated		Iris florentina L.		Sibthorp 1806-40

b) Ethnobotanical field studies

Plant name	Plant	Study region	References
iridha, krinos	Iris albicans Lange	Cyprus	Arnold-Apostolides, 1985
--	--	Thessaloniki (herbal markets)	Hanlidou, 2004
--	--	Cyprus (wild food plants)	Della, 2006
--	--	Cyprus (herbal markets)	Karousou, 2011
--	--	Cyprus (monasteries)	Lardos, 2016
--	--	North Aegean islands	Axiotis, 2018
--	--	Central Macedonia	Tsioutsiou, 2019

Reference details: see separate list

Suggested botanical identity according to the literature

Correspondence between plant name in JC and DMM

Plant name JC (Headword)	Plant name in DMM	Chapter in DMM (Wellmann edition)
κάππαρις	κάππαρις	II, 173
**Comments**: The names cited in JC and DMM are consistent.		

Information from the literature

a) Botanical texts on the plants in DMM

Plant name in DMM	Chapter in DMM (edition)	Additional names	Plant	Relevant details	Reference
κάππαρις	II, 204	καππαριά	Capparis ovata Wildenov.	plant described by Dioscorides	Billerbeck, 1824
			Capparis spinosa		
			Capparis rupestris	in Crete (acc. to Sibthorp)	
κάππαρις	II, 204	καππαριά, κάππαρα (fruit)	Capparis spinosa L. var. ovata W.		Fraas, 1845
			Capparis rupestris	on rocks near the sea shore	
κάππαρις	II, 204	καππαριά, κάππαρα (fruit)	Capparis spinosa L.		Lenz, 1859

b) Ethnobotanical field studies

Plant name	Plant	Study region	References
kapparca	Capparis spinosa L. var. canescens Cosson	Cyprus	Arnold-Apostolides, 1985
--	--	Thessaloniki (herbal markets)	Hanlidou, 2004
kappari	Capparis spinosa L.	Cyprus (wild food plants)	Della, 2006
--	--	Cyprus (herbal markets)	Karousou, 2011
καππαριά, καππαρκά	Capparis spinosa L.	Cyprus (monasteries)	Lardos, 2016
κάππαρις	Capparis spinosa L. var. spinosa	North Aegean islands	Axiotis, 2018
--	--	Central Macedonia	Tsioutsiou, 2019

Reference details: see separate list

Suggested botanical identity according to the literature

Correspondence between plant name in JC and DMM

Plant name JC (Headword)	Plant name in DMM	Chapter in DMM (Wellmann edition)
καυκαλίδα	καυκαλίς	II, 139
**Comments**: The names cited in JC and DMM are consistent.		

Information from the literature

a) Botanical texts with reference to the plants in DMM

Plant name in DMM	Chapter in DMM (edition)	Additional names	Plant	Relevant details	Reference
καύκαλις			Papaver sp.	identity stated as uncertain	Billerbeck, 1824
καυκαλίς	II, 168	Modern: καυκαλίδα	Pimpinella saxifraga L.	popular wild food in GR (rosette leaves)	Fraas, 1845
καυκαλίς	II, 168	Modern: καυκαλίδα	Pimpinella saxifraga L.		Lenz, 1859
καυκαλις			Caucalis maritima		Sibthorp 1806-40
καυκαλις			Hasselquistia aegyptiaca L.	identity stated as uncertain	Sibthorp 1806-40

b) Ethnobotanical field studies

Plant name	Plant	Study region	References
--	--	Cyprus	Arnold-Apostolides, 1985
--	--	Thessaloniki (herbal markets)	Hanlidou, 2004
--	--	Cyprus (wild food plants)	Della, 2006
--	--	Cyprus (herbal markets)	Karousou, 2011
--	--	Cyprus (monasteries)	Lardos, 2016
--	--	North Aegean islands	Axiotis, 2018
--	--	Central Macedonia	Tsioutsiou, 2019

Reference details: see separate list

Suggested botanical identity according to the literature

Correspondence between plant name in JC and DMM

Plant name JC (Headword)	Plant name in DMM	Chapter in DMM (Wellmann edition)
κευταύριον	κευταύριον το μέγα; το λεπτόν ή το μικρόν	III, 6
	κευταύριον το λεπτόν ή το μικρόν	III, 7
**Comments**: The name cited in JC is consistent with the generic name κευταύριον cited in DMM. However, it is unclear to which of the two types cited in DMM it refers. Therefore both types of DMM have to be taken into account.		

Information from the literature

a) Botanical texts with reference to the plants in DMM

Plant name in DMM	Chapter in DMM (edition)	Additional names	Plant	Relevant details	Reference
κευταύριον το μέγα	III, 8		Centaurea centaurium		Billerbeck, 1824
κευταύριον το μικρόν ή λιμναίον	III, 9	θερμόχορτον	Chironia centaurium Wildenov.		Billerbeck, 1824
κευταύριον το μικρόν ή λιμναίον	III, 9	θερμόχορτον	Gentiana centaurium L.		Billerbeck, 1824
κενταύριον το μικρόν	III, 7	θερμόχορτον, φλουσκούνι	Erythraea centaurium Pers.		Fraas, 1845
κευταύριον μέγα	III, 6	νάρκη	Centaurea centaurium L.		Lenz, 1859
κενταύριον το μικρόν και λεπτόν	III, 7	θερμόχορτον, φλουσκούνι	Gentiana centaurium L.		Lenz, 1859
κενταυριον μικρον	Not stated	θερμόχορτον	Erythraea centaurium Pers.		Sibthorp 1806-40
κενταυριον μεγα	Not stated		Centaurea centaurium		Sibthorp 1806-40

b) Ethnobotanical field studies

Plant name	Plant	Study region	References
erythraia to kentavrio, kentavria	Centaurium erythraea Rafn. ssp. rhodense	Cyprus	Arnold-Apostolides, 1985
kentavrio, thermochorto	Centaurium erythraea Rafn.	Thessaloniki (herbal markets)	Hanlidou, 2004
--	--	Cyprus (wild food plants)	Della, 2006
--	--	Cyprus (herbal markets)	Karousou, 2011
--	--	Cyprus (monasteries)	Lardos, 2016
θερμόχορτο	Centaurium erythraea Rafn.	North Aegean islands	Axiotis, 2018

--	--	Central Macedonia	Tsioutsiou, 2019

Reference details: see separate list

Suggested botanical identity according to the literature

Correspondence between plant name in JC and DMM

Plant name JC (Headword)	Plant name in DMM	Chapter in DMM (Wellmann edition)
κώνειον	κώνειον	IV, 78
**Comments**: The names cited in JC and DMM are consistent.		

Information from the literature

a) Botanical texts with reference to the plants in DMM

Plant name in DMM	Chapter in DMM (edition)	Additional names	Plant	Relevant details	Reference
κώνειον	IV, 97		Conium maculatum		Billerbeck, 1824
κώνειον	IV, 79	Modern: βρωμόχορτον	Conium maculatum L.		Fraas, 1845
κώνειον	IV, 79	Modern: βρωμόχορτον	Conium maculatum L.		Lenz, 1859
κωνειον	not mentioned	Modern: βρομόχορτον	Conium maculatum		Sibthorp 1806-40

b) Ethnobotanical field studies

Plant name	Plant	Study region	References
vromokhorton	Conium maculatum L.	Cyprus	Arnold-Apostolides, 1985
--	--	Thessaloniki (herbal markets)	Hanlidou, 2004
--	--	Cyprus (wild food plants)	Della, 2006
--	--	Cyprus (herbal markets)	Karousou, 2011
--	--	Cyprus (monasteries)	Lardos, 2016
--	--	North Aegean islands	Axiotis, 2018
--	--	Central Macedonia	Tsioutsiou, 2019

Reference details: see separate list

Suggested botanical identity according to the literature

Correspondence between plant name in JC and DMM

Plant name JC (Headword)	Plant name in DMM	Chapter in DMM (Wellmann edition)
κόνυζα	κόνυζα (μείζων, μικρά, τρίτη)	II, 121 (3 types)
**Comments**: The name cited in JC corresponds to the generic name cited in DMM. However, DMM mentioned three different types. It remains unknown to which of these types the JC plant refers to.		

Information from the literature

a) Botanical texts with reference to the plants in DMM

Plant name in DMM	Chapter in DMM (edition)	Additional names	Plant	Relevant details	Reference
κόνυζα μείζων	III, 136	Modern: κονύτζα, ψυλλίστρα	Erigeron viscosum		Billerbeck, 1824
κόνυζα μικρά	III, 136	Modern: ψυλλίστρι	Erigeron graveolens		Billerbeck, 1824
κόνυζα τρίτη	III, 136		Inula dysenterica L.		Billerbeck, 1824
κόνυζα τρίτη	III, 136		Inula britannica		Billerbeck, 1824
κόνυζα η μείζων	III, 136	Modern: κονύτζα, ψυλλίστρα	Erigeron viscosum L.		Fraas, 1845
κόνυζα μικρά	III, 136	Modern: βρωμίτζα	Erigeron graveolens L.		Fraas, 1845
κόνυζα τρίτη	III, 136		Inula britannica		Fraas, 1845
κόνυζα μείζων	III, 126		Erigeron viscosum L.		Lenz, 1859
κόνυζα μικρά	III, 126		Erigeron graveolens L.		Lenz, 1859
κονυζα μειζων	not stated		Inula viscosa		Sibthorp 1806-40
κονυζα μικρα	not stated	Modern: ψυλλίστρι	Erigeron graveolens L.		Sibthorp 1806-40
κονυζα τριτη	not stated		Inula britannica		Sibthorp 1806-40

b) Ethnobotanical field studies

Plant name	Plant	Study region	References
konyza, konyzos, pssillistra	Inula graveolens (L.) Desf.	Cyprus	Arnold-Apostolides, 1985
konyza, nerokonyzos, pssillistra	Inula viscosa (L.) Aiton	Cyprus	Arnold-Apostolides, 1985
--	--	Thessaloniki (herbal markets)	Hanlidou, 2004

--	--	Cyprus (wild food plants)	Della, 2006
--	--	Cyprus (herbal markets)	Karousou, 2011
κόνυζος	Inula viscosa (L.) Greuter	Cyprus (monasteries)	Lardos, 2016
κόνυζος	Dittrichia graveolens (L.) Greuter	Cyprus (monasteries)	Lardos, 2016
--	--	North Aegean islands	Axiotis, 2018
--	--	Central Macedonia	Tsioutsiou, 2019

Reference details: see separate list

Suggested botanical identity according to the literature

Correspondence between plant name in JC and DMM

Plant name JC (Headword)	Plant name in DMM	Chapter in DMM (Wellmann edition)
κυκλάμινος	κυκλάμινος	II, 164
**Comments**: The names cited in JC and DMM are consistent.		

Information from the literature

a) Botanical texts with reference to the plants in DMM

Plant name in DMM	Chapter in DMM (edition)	Additional names	Plant	Relevant details	Reference
κυκλάμινος	II, 194		Cyclamen europeaeum	acc. to Lenz, in Italy	Billerbeck, 1824
ετέρα κυκλάμινος	II, 195	κισσάνθεμον, κισσόφυλλον	Cyclamen hederifolium	acc. to Lenz, in Italy	Billerbeck, 1824
κυκλάμινος	II, 193	τρικλαμινός, τρικλαμίδα	Cyclamen graecum Lk.		Fraas, 1845
κυκλάμινος	II, 193	τρικλαμινός, τρικλαμίδα	Cyclamen persicum		Fraas, 1845
κυκλάμινος	II, 193	τρικλαμινός, τρικλαμίδα	Cyclamen graecum Link		Lenz, 1859
κυκλάμινος	II, 193	τρικλαμινός, τρικλαμίδα	Cyclamen L.		Lenz, 1859
κυκλαμινος	not mentioned		Cyclamen latifolium L.		Sibthorp 1806-40

b) Ethnobotanical field studies

Plant name	Plant	Study region	References
kiklaminon	Cyclamen cyprium Kotschy	Cyprus	Arnold-Apostolides, 1985
kiklaminon	Cyclamen persicum Mill.	Cyprus	Arnold-Apostolides, 1985
--	--	Thessaloniki (herbal markets)	Hanlidou, 2004
--	--	Cyprus (wild food plants)	Della, 2006
--	--	Cyprus (herbal markets)	Karousou, 2011
κυκλάμινο	Cyclamen cyprium Kotschy	Cyprus (monasteries)	Lardos, 2016
κυκλάμινο	Cyclamen persicum Mill.	Cyprus (monasteries)	Lardos, 2016
--	--	North Aegean islands	Axiotis, 2018

--	--	Central Macedonia	Tsioutsiou, 2019

Reference details: see separate list

Suggested botanical identity according to the literature

Correspondence between plant name in JC and DMM

Plant name JC (Headword)	Plant name in DMM	Chapter in DMM (Wellmann edition)
κύπερος	κύπερος	I, 4
**Comments**: The names cited in JC and DMM are consistent.		

Information from the literature

a) Botanical texts with reference to the plants in DMM

Plant name in DMM	Chapter in DMM (edition)	Additional names	Plant	Relevant details	Reference
κύπειρος	I, 4	κυπειρόν, κύπειρη	Cyperus rotundus		Billerbeck, 1824
κύπειρος	I, 4	κυπειρόν, κυπειρίς	Cyperus longus		Billerbeck, 1824
κύπειρος	I, 4	κύπειρον; κύπειρι	Cyperus rotundus L.		Fraas, 1845
--	--	Theophr.: κύπειρος	Cyperus longus L.		Fraas, 1845
--	--	Theophr.: κύπειρον; κύπειρι	Cyperus rotundus L.		Lenz, 1859
--	--	Theophr.: κύπειρος; άγριον κύπειρι	Cyperus longus L.		Lenz, 1859
κυπερος	Not stated	κύπειρη	Cyperus rotundus L.		Sibthorp 1806-40
Not stated	Not stated	κύπειρος	Cyperus longus L.		Sibthorp 1806-40

b) Ethnobotanical field studies

Plant name	Plant	Study region	References
kyperos	Cyperus longus L.	Cyprus	Arnold-Apostolides, 1985
kyperos, karpourtin	Cyperus rotundus L.	Cyprus	Arnold-Apostolides, 1985
--	--	Thessaloniki (herbal markets)	Hanlidou, 2004
--	--	Cyprus (wild food plants)	Della, 2006
--	--	Cyprus (herbal markets)	Karousou, 2011
--	--	Cyprus (monasteries)	Lardos, 2016
--	--	North Aegean islands	Axiotis, 2018

--	--	Central Macedonia	Tsioutsiou, 2019

Reference details: see separate list

Suggested botanical identity according to the literature

Correspondence between plant name in JC and DMM

Plant name JC (Headword)	Plant name in DMM	Chapter in DMM (Wellmann edition)
κύπρος	κύπρος	I, 95
**Comments**: The names cited in JC and DMM are consistent.		

Information from the literature

a) Botanical texts with reference to the plants in DMM

Plant name in DMM	Chapter in DMM (edition)	Additional names	Plant	Relevant details	Reference
κύπρος	I, 124		Lawsonia inermis L.		Billerbeck, 1824
κύπρος	I, 124	Modern: χέννα	Lawsonia alba Lam.	Not found in Greece	Fraas, 1845
κύπρος	I, 124		Lawsonia alba Lam.		Lenz, 1859
					Sibthorp 1806-40
					Sibthorp 1806-40

b) Ethnobotanical field studies

Plant name	Plant	Study region	References
kypross, henna	Lawsonia inermis L.	Cyprus	Arnold-Apostolides, 1985
henna	Lawsonia inermis L.	Thessaloniki (herbal markets)	Hanlidou, 2004
--	--	Cyprus (wild food plants)	Della, 2006
--	--	Cyprus (herbal markets)	Karousou, 2011
--	--	Cyprus (monasteries)	Lardos, 2016
--	--	North Aegean islands	Axiotis, 2018
--	--	Central Macedonia	Tsioutsiou, 2019

Reference details: see separate list

Suggested botanical identity according to the literature

Correspondence between plant name in JC and DMM

Plant name JC (Headword)	Plant name in DMM	Chapter in DMM (Wellmann edition)
λειχήνη	λειχήνη, see μυρσίνη αγρία	IV, 144
**Comments**: The name cited in JC corresponds to one of the synonyms mentioned in DMM for μυρσίνη αγρία.		

Information from the literature

a) Botanical texts with reference to the plants in DMM

Plant name in DMM	Chapter in DMM (edition)	Additional names	Plant	Relevant details	Reference
μυρσίνη αγρία	IV, 146	DMM: οζυμυρσίνη, κέντρομυρσίνη	Ruscus aculeatus L.		Billerbeck, 1824
μυρσίνη αγρία	IV, 144	DMM: οζυμυρσίνη	Ruscus aculeatus L.		Fraas, 1845
μυρσίνη αγρία	IV, 144	DMM: οζυμυρσίνη, μυρτάκανθα;<br>Modern: λαγομηλεά, κοραλλόχορτον, σμυρνάκανθα	Ruscus aculeatus L.		Lenz, 1859
μυρσινη αγρια	not stated	Modern: λαγομιλιά	Ruscus aculeatus L.		Sibthorp 1806-40

b) Ethnobotanical field studies

Plant name	Plant	Study region	References
aghriomerssini, akromersini, laghomillya, myrisini agrio, myrtakantha, kourelya, pirnari	Ruscus aculeatus L.	Cyprus	Arnold-Apostolides, 1985
--	--	Thessaloniki (herbal markets)	Hanlidou, 2004
--	--	Cyprus (wild food plants)	Della, 2006
--	--	Cyprus (herbal markets)	Karousou, 2011
αρκομερσίνι, περνάρι	Ruscus aculeatus L.	Cyprus (monasteries)	Lardos, 2016
--	--	North Aegean islands	Axiotis, 2018
agathi	Ruscus aculeatus L.	Central Macedonia	Tsioutsiou, 2019

Reference details: see separate list

Suggested botanical identity according to the literature

Correspondence between plant name in JC and DMM

Plant name JC (Headword)	Plant name in DMM	Chapter in DMM (Wellmann edition)
μανδραγόρας	μανδραγόρας	IV, 75
**Comments**: The names cited in JC and DMM are consistent.		

Information from the literature

a) Botanical texts with reference to the plants in DMM

Plant name in DMM	Chapter in DMM (edition)	Additional names	Plant	Relevant details	Reference
μανδραγόρας μέλας	IV, 76	DMM: θρυδακίας, αντίμηλον	Atropa mandragoras		Billerbeck, 1824
μανδραγόρας μέλας	III, 134	DMM: κιρκαία, αντίμηλον	Atropa mandragora L. var. autumnalis	flowering in autumn	Fraas, 1845
μανδραγόρας μέλας	III, 134	DMM: κιρκαία, αντίμηλον	Atropa mandragora L. var. vernalis	flowering in spring	Fraas, 1845
μανδραγόρας	IV, 76		Atropa mandragora L.		Lenz, 1859
μανδραγόρας θρυδάκιας			Mandragora autumnalis		Sibthorp 1806-40
μανδραγόρας άρρεν			Mandragora vernalis		Sibthorp 1806-40
--		Modern: μανδραγούρα	Mandragora officinalis		Sibthorp 1806-40

b) Ethnobotanical field studies

Plant name	Plant	Study region	References
mandhraghoras, kalathroposs	Mandragora officinarum L.	Cyprus	Arnold-Apostolides, 1985
--	--	Thessaloniki (herbal markets)	Hanlidou, 2004
--	--	Cyprus (wild food plants)	Della, 2006
--	--	Cyprus (herbal markets)	Karousou, 2011
μανδραγόρας, καλάνθρωπος	Mandragora officinarum L.	Cyprus (monasteries)	Lardos, 2016
μανδραγούδας	Mandragora officinarum L.	North Aegean islands	Axiotis, 2018
--	--	Central Macedonia	Tsioutsiou, 2019

Reference details: see separate list

Suggested botanical identity according to the literature

Correspondence between plant name in JC and DMM

Plant name JC (Headword)	Plant name in DMM	Chapter in DMM (Wellmann edition)
μελάνθιον	μελάνθιον	III, 79
**Comments**: The names cited in JC and DMM are consistent.		

Information from the literature

a) Botanical texts on the plants in DMM

Plant name in DMM	Chapter in DMM (edition)	Additional names cited	Plant	Relevant details	Reference
μελάνθιον	III, 93	μαυροκουκολις (Cyprus)	Nigella sativa		Billerbeck, 1824
μελάνθιον	III, 93	μαυροκούκκι, μαυρο-κούκουλι	Nigella sativa L.		Fraas, 1845
μελάνθιον	III, 93	μαυροκούκκι, μαυρο-κούκουλι	Nigella sativa L.		Lenz, 1859

b) Ethnobotanical field studies

Plant name	Plant	Study region	References
mavrococco	Nigella sativa L.	Cyprus	Arnold-Apostolides, 1985
mavrococco	Nigella damascena L.	Cyprus	Arnold-Apostolides, 1985
--	--	Thessaloniki (herbal markets)	Hanlidou, 2004
--	--	Cyprus (wild food plants)	Della, 2006
--	--	Cyprus (herbal markets)	Karousou, 2011
μαυρόκοκκο	Nigella sativa L.	Cyprus (monasteries)	Lardos, 2016
μαυρόκοκκο	Nigella damascena L.	Cyprus (monasteries)	Lardos, 2016
--	--	North Aegean islands	Axiotis, 2018
--	--	Central Macedonia	Tsioutsiou, 2019

Reference details: see separate list

Suggested botanical identity according to the literature

Correspondence between plant name in JC and DMM

Plant name JC (Headword)	Plant name in DMM	Chapter in DMM (Wellmann edition)
πολυπόδιον	πολυπόδιον	I, 186
**Comments**: The names cited in JC and DMM are consistent.		

Information from the literature

a) Botanical texts with reference to the plants in DMM

Plant name in DMM	Chapter in DMM (edition)	Additional names	Plant	Relevant details	Reference
πολυπόδιον	IV, 188		Polypodium vulgare		Billerbeck, 1824
πολυπόδιον	IV, 188		Polypodium phegopteris		Billerbeck, 1824
πολυπόδιον	IV, 185	Modern: πολυπόδι, δενδροφθείρι	Polypodium vulgare L.		Fraas, 1845
πολυπόδιον	IV, 185	Modern: πολυπόδι, δενδροφθείρι	Polypodium vulgare L.		Lenz, 1859
πολυπόδιον		Modern: πολυπόδι, δενδροφθήρι	Polypodium vulgare L.		Sibthorp 1806-40
					Sibthorp 1806-40

b) Ethnobotanical field studies

Plant name	Plant	Study region	References
polypodion, dhentrophtiri	Polypodium cambricum L.	Cyprus	Arnold-Apostolides, 1985
--	--	Thessaloniki (herbal markets)	Hanlidou, 2004
--	--	Cyprus (wild food plants)	Della, 2006
--	--	Cyprus (herbal markets)	Karousou, 2011
--	--	Cyprus (monasteries)	Lardos, 2016
--	--	North Aegean islands	Axiotis, 2018
--	--	Central Macedonia	Tsioutsiou, 2019

Reference details: see separate list

Suggested botanical identity according to the literature

Correspondence between plant name in JC and DMM

Plant name JC (Headword)	Plant name in DMM	Chapter in DMM (Wellmann edition)
πράσιον	πράσιον	III, 105
**Comments**: The names cited in JC and DMM are consistent.		

Information from the literature

a) Botanical texts with reference to the plants in DMM

Plant name in DMM	Chapter in DMM (edition)	Additional names	Plant	Relevant details	Reference
πράσιον	III, 119	Modern: σκυλόχορτον	Marrubium vulgare		Billerbeck, 1824
πράσιον	III, 109	Modern: σκυλόχορτον	Marrubium vulgare		Fraas, 1845
πράσιον	III, 109		Marrubium vulgare L.		Lenz, 1859
πρασιον	not mentioned		Marrubium vulgare L.		Sibthorp 1806-40

b) Ethnobotanical field studies

Plant name	Plant	Study region	References
--	--	Cyprus	Arnold-Apostolides, 1985
marrouvio	Marrubium vulgare L.	Thessaloniki (herbal markets)	Hanlidou, 2004
--	--	Cyprus (wild food plants)	Della, 2006
--	--	Cyprus (herbal markets)	Karousou, 2011
--	--	Cyprus (monasteries)	Lardos, 2016
--	--	North Aegean islands	Axiotis, 2018
--	--	Central Macedonia	Tsioutsiou, 2019

Reference details: see separate list

Suggested botanical identity according to the literature

Correspondence between plant name in JC and DMM

Plant name JC (Headword)	Plant name in DMM	Chapter in DMM (Wellmann edition)
ράμνος	ράμνος	I, 90 (3 types)
**Comments**: The name cited in JC is consistent with the generic term cited in DMM, but can refer to any of the three types of ράμνος mentioned in DMM.		

Information from the literature

a) Botanical texts with reference to the plants in DMM

Plant name in DMM	Chapter in DMM (edition)	Additional names	Plant	Relevant details	Reference
ράμνος μέλας	I, 119		Rhamnus lycioides		Billerbeck, 1824
ράμνος λευκός	I, 119	Modern: ράμνος (?)	Lycium europaeum L.	Fraas (p. 93) disagrees	Billerbeck, 1824
ράμνος τρισσός	I, 119		Rhamnus paliurus		Billerbeck, 1824
ράμνος	I, 119		Rhamnus oleoides L.		Fraas, 1845
ράμνος έτερα λευκότερα	I, 119		Rhamnus saxatilis L.		Fraas, 1845
ράμνος τρίτη	I, 119		Paliurus australis G.		Fraas, 1845
ράμνος	I, 119		Rhamnus spp.		Lenz, 1859
--	--	Theophrastus: ράμνος	Rhamnus oleoides L.	The type with “evergreen leaves”	Lenz, 1859
ραμνος	not mentioned	Modern: ράμνος (?)	Lycium europaeum L.	Fraas (p. 93) disagrees	Sibthorp 1806-40
ραμνος τρισσος	not mentioned		Palurus aculeatus		Sibthorp 1806-40

b) Ethnobotanical field studies

Plant name	Plant	Study region	References
--	--	Cyprus	Arnold-Apostolides, 1985
paliouri	Paliurus spina-christi Mill.	Thessaloniki (herbal markets)	Hanlidou, 2004
ramnos, frangula, cascara	Rhamnus purshiana DC.	Thessaloniki (herbal markets)	Hanlidou, 2004
--	--	Cyprus (wild food plants)	Della, 2006

--	--	Cyprus (herbal markets)	Karousou, 2011
--	--	Cyprus (monasteries)	Lardos, 2016
--	--	North Aegean islands	Axiotis, 2018
paliuri, fluri	Paliurus spina-christi Mill.	Central Macedonia	Tsioutsiou, 2019

Reference details: see separate list

Suggested botanical identity according to the literature

Correspondence between plant name in JC and DMM

Plant name JC (Headword)	Plant name in DMM	Chapter in DMM (Wellmann edition)
ροδάφνη	ροδοδάφνη, see νέριον	IV, 81
**Comments**: The names cited in JC and DMM are consistent.		

Information from the literature

a) Botanical texts with reference to the plants in DMM

Plant name in DMM	Chapter in DMM (edition)	Additional names	Plant	Relevant details	Reference
ροδοδάφνη	IV, 82	DMM: νέριον	Nerium oleander		Billerbeck, 1824
ροδοδάφνη	IV, 82	DMM: νέριον	Nerium oleander L.		Fraas, 1845
ροδοδάφνη	IV, 82	DMM: νέριον; Modern: πικροδάφνη	Nerium oleander L.		Lenz, 1859
νήριον		Modern: ροδοδάφνη	Nerium oleander L.		Sibthorp 1806-40

b) Ethnobotanical field studies

Plant name	Plant	Study region	References
rhodhodhaffni	Nerium oleander L.	Cyprus	Arnold-Apostolides, 1985
--	--	Thessaloniki (herbal markets)	Hanlidou, 2004
--	--	Cyprus (wild food plants)	Della, 2006
--	--	Cyprus (herbal markets)	Karousou, 2011
αροδάφνη, ροδοδάφνη	Nerium oleander L.	Cyprus (monasteries)	Lardos, 2016
--	--	North Aegean islands	Axiotis, 2018
--	--	Central Macedonia	Tsioutsiou, 2019

Reference details: see separate list

Suggested botanical identity according to the literature

Correspondence between plant name in JC and DMM

Plant name JC (Headword)	Plant name in DMM	Chapter in DMM (Wellmann edition)
ρούδιν	ρούς	I, 108
**Comments**: The name cited in JC corresponds to the modern variant of the ancient plant name mentioned in DMM.		

Information from the literature

a) Botanical texts with reference to the plants in DMM

Plant name in DMM	Chapter in DMM (edition)	Additional names	Plant	Relevant details	Reference
ρούς	I, 147		Rhus coriaria		Billerbeck, 1824
ρούς	I, 147	Modern: βυρσιά, βυρσόκλαδα	Rhus coriaria L.		Fraas, 1845
ρούς	I, 147	Modern: βυρσιά, βυρσόκλαδα	Rhus coriaria L.		Lenz, 1859
ρους	not mentioned	Modern: sumach (Turkish)	Rhus coriaria L.		Sibthorp 1806-40

b) Ethnobotanical field studies

Plant name	Plant	Study region	References
roudhi, rouin, soumaki, sumadjin, virssya	Rhus coriaria L.	Cyprus	Arnold-Apostolides, 1985
--	--	Thessaloniki (herbal markets)	Hanlidou, 2004
--	--	Cyprus (wild food plants)	Della, 2006
--	--	Cyprus (herbal markets)	Karousou, 2011
ρούδι, ρούβι, ρούι, σουμάτζι	Rhus coriaria L.	Cyprus (monasteries)	Lardos, 2016
σουμάκι	Rhus coriaria L.	North Aegean islands	Axiotis, 2018
--	--	Central Macedonia	Tsioutsiou, 2019

Reference details: see separate list

Suggested botanical identity according to the literature

Correspondence between plant name in JC and DMM

Plant name JC (Headword)	Plant name in DMM	Chapter in DMM (Wellmann edition)
σιδερίτις	σιδερίτις	IV, 33
**Comments**: The names cited in JC and DMM are consistent.		

Information from the literature

a) Botanical texts with reference to the plants in DMM

Plant name in DMM	Chapter in DMM (edition)	Additional names	Plant	Relevant details	Reference
χαμαίπιτυς	III, 175	Pliny: σιδηρίτις, Achillea quarta;<br>Modern (Euböa): σιδερίτις	Ajuga reptans	Fraas disagrees	Billerbeck, 1824
σιδηρίτις	IV, 33		Sideritis romana		Billerbeck, 1824
--	--	Pliny: σιδηρίτις, Achillea quinta	Sideritis montana		Billerbeck, 1824
σιδηρίτις ηρακλεία	not mentioned		Stachys recta		Billerbeck, 1824
--	--	--	--		Fraas, 1845
--	--	--	--		Lenz, 1859
σιδεριτις	not mentioned		Stachys recta L.		Sibthorp 1806-40

b) Ethnobotanical field studies

Plant name	Plant	Study region	References
--	--	Cyprus	Arnold-Apostolides, 1985
--	--	Thessaloniki (herbal markets)	Hanlidou, 2004
--	--	Cyprus (wild food plants)	Della, 2006
sideritis	Sideritis cypria Post.	Cyprus (herbal markets)	Karousou, 2011
sideritis	Sideritis perfoliata L.	Cyprus (herbal markets)	Karousou, 2011
σιδερίτις, τσάϊ του βουνού	Sideritis perfoliata L.	Cyprus (monasteries)	Lardos, 2016
σιδερίτις	Sideritis sipylea Boiss.	North Aegean islands	Axiotis, 2018

aspro tsai	Sideritis montana ssp. remota (d'Urv.) F.W.Ball	Central Macedonia	Tsioutsiou, 2019
tsai tou vounou	Sideritis scardica Griseb.	Central Macedonia	Tsioutsiou, 2019

Reference details: see separate list

Suggested botanical identity according to the literature

Correspondence between plant name in JC and DMM

Plant name JC (Headword)	Plant name in DMM	Chapter in DMM (Wellmann edition)
χαμαίδαφνη	χαμαίδαφνη	IV, 144
**Comments**: The name cited in JC corresponds 1) to the first name mnetioned in DMM chapter IV, 147 and, 2) to one of the synonyms for δαφνοειδές mentioned in DMM chapter IV, 146.		

Information from the literature

a) Botanical texts with reference to the plants in DMM

Plant name in DMM	Chapter in DMM (edition)	Additional names	Plant	Relevant details	Reference
not mentioned	IV, 149	Various Greek-Roman authors: χαμαίδαφνη	Ruscus hypophyllum		Billerbeck, 1824
--	--	Various Greek-Roman authors: χαμαίδαφνη	Ruscus hypophaë L.		Billerbeck, 1824
--	--	Theophr. h. p. 3,18: χαμαιδάφνη	Ruscus racemosus		Fraas, 1845
--	--		--		Lenz, 1859
χαμαιδαφνη	not mentioned	Modern: κοραλλοβότανον	Ruscus hypophyllum		Sibthorp 1806-40

b) Ethnobotanical field studies

Plant name	Plant	Study region	References
--	--	Cyprus	Arnold-Apostolides, 1985
--	--	Thessaloniki (herbal markets)	Hanlidou, 2004
--	--	Cyprus (wild food plants)	Della, 2006
--	--	Cyprus (herbal markets)	Karousou, 2011
--	--	Cyprus (monasteries)	Lardos, 2016
--	--	North Aegean islands	Axiotis, 2018
--	--	Central Macedonia	Tsioutsiou, 2019

Reference details: see separate list

Suggested botanical identity according to the literature

Correspondence between plant name in JC and DMM

Plant name JC (Headword)	Plant name in DMM	Chapter in DMM (Wellmann edition)
χαμαίμιλον, χαμαίμηλα	ανθεμίς: χαμαίμηλον	III, 137
**Comments**: The names cited in JC are consistent with one of the types of ανθεμίς cited in DMM.		

Information from the literature

a) Botanical texts with reference to the plants in DMM

Plant name in DMM	Chapter in DMM (edition)	Additional names	Plant	Relevant details	Reference
ανθεμίς: λευκάνθεμος, χαμαίμηλον	III, 154	CY: παπούνι	Anthemis chia		Billerbeck, 1824
ανθεμίς: λευκάνθεμος, χαμαίμηλον	III, 144	χαμομηλιά	Matricaria chamomilla L.		Fraas, 1845
ανθεμίς: λευκάνθεμος, χαμαίμηλον	III, 144	χαμομηλεά	Matricaria chamomilla L.		Lenz, 1859
ανθεμίς: χαμαίμηλον	Not stated		Anthemis chia L.		Sibthorp 1806-40
--	--	χαμόμηλα	Matricaria chamomilla L.		Sibthorp 1806-40

b) Ethnobotanical field studies

Plant name	Plant	Study region	References
khamomilli, asprokhorto	Matricaria recutitia L. var. coronata (Gay ex Boiss.) Gruenb.-Fertig	Cyprus	Arnold-Apostolides, 1985
hamomilo, hamomili	Chamomilla recutita (L.) Rauschert	Thessaloniki (herbal markets)	Hanlidou, 2004
--	--	Cyprus (wild food plants)	Della, 2006
chamomili	Matricaria chamomilla L.	Cyprus (herbal markets)	Karousou, 2011
χαμομήλι	Matricaria recutitia L.	Cyprus (monasteries)	Lardos, 2016
χαμομήλι	Matricaria chamomilla L.	North Aegean islands	Axiotis, 2018
chamomili	Matricaria chamomilla L.	Central Macedonia	Tsioutsiou, 2019

Reference details: see separate list

Suggested botanical identity according to the literature

Correspondence between plant name in JC and DMM

Plant name JC (Headword)	Plant name in DMM	Chapter in DMM (Wellmann edition)
χαμαίπιτυς	χαμαίπιτυς / έτερα χαμαίπιτυς / τρίτη χαμαίπιτυς	III, 158 (3 types)
**Comments**: The name cited in JC corresponds to the generic name of the three types of plants mentioned in the respective chapter.		

Information from the literature

a) Botanical texts with reference to the plants in DMM

Plant name in DMM	Chapter in DMM (edition)	Additional names	Plant	Relevant details	Reference
χαμαίπιτυς	III, 175		Ajuga reptans	Fraas disagrees	Billerbeck, 1824
χαμαίπιτυς secunda	III, 176		Teucrium iva		Billerbeck, 1824
χαμαίπιτυς τρίτη	III, 176		Teucrium chamaepitys		Billerbeck, 1824
υπέρικον	III, 171	DMM: χαμαίπιτυς	Hypericum crispum		Billerbeck, 1824
χαμαίπιτυς	III, 165		Ajuga iva L.		Fraas, 1845
χαμαίπιτυς ετέρα	III, 166	Theophr. h.p. 6,2.1,16: κνέωρος μέλας; Modern: άγρια γερόκλαδα	Passerina hirsuta L.	Sibthorp disagrees	Fraas, 1845
χαμαίπιτυς τρίτη	III, 166		Ajuga chia L.		Fraas, 1845
χαμαίπιτυς	III, 165	Modern: pityorysis, orizelos, olokyron, ionia, sideritis,	Ajuga iva Schreber		Lenz, 1859
χαμαίπιτυς	III, 165		Teucrium iva L.		Lenz, 1859
χαμαίπιτυς τρίτη	III, 166		Ajuga chia Schreber		Lenz, 1859
χαμαιπιτυς	III, 175		Ajuga iva Schreb.		Sibthorp 1806-40
χαμαίπιτυς τρίτη	not mentioned	Modern: δοκεκάνθη	Ajuga chamaepitys Schreb.		Sibthorp 1806-40

b) Ethnobotanical field studies

Plant name	Plant	Study region	References
--	--	Cyprus	Arnold-Apostolides, 1985
--	--	Thessaloniki (herbal markets)	Hanlidou, 2004

--	--	Cyprus (wild food plants)	Della, 2006
--	--	Cyprus (herbal markets)	Karousou, 2011
--	--	Cyprus (monasteries)	Lardos, 2016
--	--	North Aegean islands	Axiotis, 2018
--	--	Central Macedonia	Tsioutsiou, 2019

Reference details: see separate list

Suggested botanical identity according to the literature

Correspondence between plant name in JC and DMM

Plant name JC (Headword)	Plant name in DMM	Chapter in DMM (Wellmann edition)
χελιδονεα	χελιδόνιον μέγα	II, 180
χελιδονεα	χελιδόνιον το μικρόν	II, 181
**Comments**: The name cited in JC is consistent with the generic name cited in DMM, but can refer to both types of χελιδόνιον mentioned and DMM.		

Information from the literature

a) Botanical texts with reference to the plants in DMM

Plant name in DMM	Chapter in DMM (edition)	Additional names	Plant	Relevant details	Reference
χελιδόνιον μέγα	2, 211		Chelidonium majus		Billerbeck, 1824
χελιδόνιον μέγα	2, 211		Chelidonium quercifolium		Billerbeck, 1824
χελιδόνιον το μικρόν	2, 211		Ranunculus ficaria		Billerbeck, 1824
χελιδόνιον μέγα	2, 211		Chelidonium majus L.	rare in Greece, abundant in Italy	Fraas, 1845
χελιδόνιον το μικρόν	2, 212	Modern: σφουρδάκλα	Ranunculus ficaria L.	rare in Greece, abundant in Italy	Fraas, 1845
χελιδόνιον μέγα	2, 212		Chelidonium majus L.	rare in Greece, abundant in Italy	Lenz, 1859
χελιδόνιον το μικρόν	2, 212		Ranunculus ficaria L.		Lenz, 1859
χελιδονιον	not mentioned	Modern: χελιδόνιον ?	Chelidonium majus	Fraas doubtful about the modern name	Sibthorp 1806-40
χελιδόνιον μικρόν	not mentioned		Ranunculus ficaria		Sibthorp 1806-40

b) Ethnobotanical field studies

Plant name	Plant	Study region	References
sfourdhakla	Ranunculus ficaria L.	Cyprus	Arnold-Apostolides, 1985
--	--	Thessaloniki (herbal markets)	Hanlidou, 2004
--	--	Cyprus (wild food plants)	Della, 2006
chelidonio	Chelidonium majus L.	Cyprus (herbal markets)	Karousou, 2011
--	--	Cyprus (monasteries)	Lardos, 2016

--	--	North Aegean islands	Axiotis, 2018
--	--	Central Macedonia	Tsioutsiou, 2019

Reference details: see separate list

Suggested botanical identity according to the literature

Correspondence between plant name in JC and DMM

Plant name JC (Headword)	Plant name in DMM	Chapter in DMM (Wellmann edition)
σκολοπένδριον, σκολόπενδρα	σκολοπένδριον, see άσπληνος	III, 134
**Comments**: The names cited in JC and DMM are consistent.		

Information from the literature

a) Botanical texts with reference to the plants in DMM

Plant name in DMM	Chapter in DMM (edition)	Additional names	Plant	Relevant details	Reference
σκολοπένδριον	III, 151	DMM: σκολόπενδρα, άσπληνον, ημιόνιον	Ceterach officinarum Wildenov.		Billerbeck, 1824
σκολοπένδριον	III, 141	DMM: σκολόπενδρα, άσπληνον, ημιόνιον	Asplenium ceterach L.		Fraas, 1845
σκολοπένδριον	III, 141	DMM: άσπληνον, ημιόνιον; Modern: σκορπίδι, χρυσόχορτον	Asplenium ceterach L.		Lenz, 1859
ασπλήνιον		Modern: σκορπίδι, χρυσόχορτον	Gymnogramma ceterach		Sibthorp 1806-40

b) Ethnobotanical field studies

Plant name	Plant	Study region	References
skorpiti, khrissokhorto	Asplenium ceterach L.	Cyprus	Arnold-Apostolides, 1985
skorpidi	Asplenium ceterach L.	Thessaloniki (herbal markets)	Hanlidou, 2004
--	--	Cyprus (wild food plants)	Della, 2006
--	--	Cyprus (herbal markets)	Karousou, 2011
--	--	Cyprus (monasteries)	Lardos, 2016
--	--	North Aegean islands	Axiotis, 2018
--	--	Central Macedonia	Tsioutsiou, 2019

Reference details: see separate list

Suggested botanical identity according to the literature

Correspondence between plant name in JC and DMM

Plant name JC (Headword)	Plant name in DMM	Chapter in DMM (Wellmann edition)
σκορπίουρον	σκορπίουρον, see ηλιοτρόπιον το μέγα	IV, 190
**Comments**: The name cited in JC is consistent with one of the synonyms cited in DMM for ηλιοτρόπιον το μέγα.		

Information from the literature

a) Botanical texts with reference to the plants in DMM

Plant name in DMM	Chapter in DMM (edition)	Additional names	Plant	Relevant details	Reference
σκορπίουρος	IV, 193	DMM: ηλιοτρόπιον το μέγα<br>Modern: ηλιοτρόπιον, βρομόχορτον	Heliotropium europaeum		Billerbeck, 1824
σκορπίουρος	IV, 190	DMM: ηλιοτρόπιον το μέγα<br>Modern: βρωμόχορτον	Heliotropium villosum Desf.		Fraas, 1845
--	--	--	--	Pages missing in Lenz PDF	Lenz, 1859
ηλιοτροπιον μεγα	not mentioned	Modern: ηλιοτρόπιον, βρομόχορτον	Heliotropium europaeum		Sibthorp 1806-40

b) Ethnobotanical field studies

Plant name	Plant	Study region	References
--	--	Cyprus	Arnold-Apostolides, 1985
--	--	Thessaloniki (herbal markets)	Hanlidou, 2004
--	--	Cyprus (wild food plants)	Della, 2006
--	--	Cyprus (herbal markets)	Karousou, 2011
--	--	Cyprus (monasteries)	Lardos, 2016
--	--	North Aegean islands	Axiotis, 2018
--	--	Central Macedonia	Tsioutsiou, 2019

Reference details: see separate list

Suggested botanical identity according to the literature

Correspondence between plant name in JC and DMM

Plant name JC (Headword)	Plant name in DMM	Chapter in DMM (Wellmann edition)
φλόμος	φλόμος	IV, 103
**Comments**: The name cited in JC is consistent with the generic name cited in DMM, but can refer to any of the types of φλόμος mentioned in DMM.		

Information from the literature

a) Botanical texts with reference to the plants in DMM

Plant name in DMM	Chapter in DMM (edition)	Additional names	Plant	Relevant details	Reference
φλόμος, φλομίς λευκή άρρην	IV, 104		Verbascum thapsus		Billerbeck, 1824
φλόμος, φλομίς λευκή θηλεία	IV, 104		Verbascum plicatum		Billerbeck, 1824
φλόμος μέλας	IV, 104		Verbascum sinuatum		Billerbeck, 1824
φλόμος, φλομίς αγρία	IV, 104	Modern: φλόμο	Phlomis fruticosa L.		Billerbeck, 1824
φλόμος, φλόμίς αγρία	IV, 104	DMM: λυχνίτις, θρυαλλίς	Phlomis lychnitis L.		Billerbeck, 1824
φλόμος, φλομίδες δίπλαι, hirsutae, humilis	IV, 104		Primula veris L.,		Billerbeck, 1824
φλόμος, φλομίδες δίπλαι, hirsutae, humilis	IV, 104		Primula elatior L.		Billerbeck, 1824
φλόμος λευκή η άρρην	IV, 102		Verbascum thapsus L.	rare in Greece	Fraas, 1845
φλόμος λευκή η θηλεία	IV, 102	Modern: γλώσσα, φτιλεά	Verbascum plicatum Sibth.		Fraas, 1845
φλόμος η μέλαινα	IV, 102	Modern: γλώσσα	Verbascum sinuatum L.		Fraas, 1845
φλόμος αγρία	IV, 102		Phlomis fruticosa L.		Fraas, 1845
φλόμος, φλομίς λυχνίτις	IV, 102	DMM: θρυαλλίς	Verbascum limnense M.		Fraas, 1845
φλόμος, φλομίδες διπλαί	IV, 102		Sideritis romana L.		Fraas, 1845
φλόμος, φλομίδες διπλαί	IV, 102		Sideritis elegans L.		Fraas, 1845
φλόμος	IV, 102	Modern: γαιδαρόσφακα	Verbascum thapsus L.,	rare in Greece	Lenz, 1859
φλόμος	IV, 102	Modern: γαιδαρόσφακα	Verbascum phlomoides L.	rare in Greece	Lenz, 1859
φλόμος	IV, 102	Modern: γαιδαρόσφακα	Verbascum sinuatum L.		Lenz, 1859

φλόμος	IV, 102	Modern: γαιδαρόσφακα	Verbascum plicatum Sibth.		Lenz, 1859
φλόμος αγρία	IV, 102	Modern: γαιδαρόσφακα	Phlomis fruticosa L.		Lenz, 1859
φλομος λευκη αρρην	not mentioned		Verbascum thapsus L.		Sibthorp 1806-40
φλομος λευκη θηλη	IV, 104	Modern: φλόμος	Verbascum plicatum		Sibthorp 1806-40
φλομος μελας	IV, 104	Modern: φλόμος	Verbascum sinuatum L.		Sibthorp 1806-40
φλομος μελας	not mentioned		Verbascum nigrum		Sibthorp 1806-40
φλομος αγρια	not mentioned	Modern: φλομό, σφάκα, γαδαρόσφακα	Phlomis fruticosa L.		Sibthorp 1806-40

b) Ethnobotanical field studies

**Plant name**	**Plant**	**Study region**	**References**
phlomos, splonos, ghlossa	Verbascum sinuatum L.	Cyprus	Arnold-Apostolides, 1985
primula	Primula veris L.	Thessaloniki (herbal markets)	Hanlidou, 2004
--	--	Cyprus (wild food plants)	Della, 2006
--	--	Cyprus (herbal markets)	Karousou, 2011
--	--	Cyprus (monasteries)	Lardos, 2016
--	--	North Aegean islands	Axiotis, 2018
--	--	Central Macedonia	Tsioutsiou, 2019

Reference details: see separate list

Suggested botanical identity according to the literature

Correspondence between plant name in JC and DMM

Plant name JC (Headword)	Plant name in DMM	Chapter in DMM (Wellmann edition)
στρύχνον (το λεγομένον βρωμοβότανον, οπου ενεν τα φυλλα του μικρα μαυρουτζικα)	στρύχνον κηπαίων	IV, 70
**Comments**: DMM describes various types of στρύχνον. In JC it is mentioned that the type having “small and dark leaves” (οπου ενεν τα φυλλα του μικρα μαυρουτζικα) should be used. Of all the types described in DMM, this essentially fits only to στρύχνον κηπαίων. The part used in the corresponding recipes in JC, being the leaf or the juice of the herb, also corresponds to the parts used of this type in DMM.		

Information from the literature

a) Botanical texts with reference to the plants in DMM

Plant name in DMM	Chapter in DMM (edition)	Additional names	Plant	Relevant details	Reference
στρύχνος κηπαίος	IV, 71-74	Theophr.: στρύνος ήμερος; Modern: αγριομελιττάνα, μελισάνα	Solanum melongena L.		Billerbeck, 1824
στρύχνος μανικός	IV, 71-74		Solanum nigrum		Billerbeck, 1824
στρύχνος κηπαίος	IV, 71		Solanum nigrum L.		Fraas, 1845
στρύχνος κηπαίος	IV, 71		Solanum flavum		Fraas, 1845
στρύχνος κηπαίος	IV, 71	DMM: στρύχνος εδώδιμος	Solanum melongena L.		Lenz, 1859
στρύχνος κηπαίος	IV, 71		Solanum nigrum L.		Lenz, 1859
στρύχνος κηπαίος			Solanum nigrum L.		Sibthorp 1806-40

b) Ethnobotanical field studies

Plant name	Plant	Study region	References
strikhnos o melas, strifnos, pomilokhorton	Solanum nigrum L.	Cyprus	Arnold-Apostolides, 1985
strifnos, pomilokhorton	Solanum villosum Mill.	Cyprus	Arnold-Apostolides, 1985
strikhnos i melinzana, melintzana, batlindjana, vasanya	Solanum melongena L.	Cyprus	Arnold-Apostolides, 1985

Plant name	Plant	Study region	References
--	--	Thessaloniki (herbal markets)	Hanlidou, 2004
fyto ai giorkou	Solanum nigrum L.	Cyprus (wild food plants)	Della, 2006
pomiloroua	Solanum villosum Mill.	Cyprus (wild food plants)	Della, 2006
--	--	Cyprus (herbal markets)	Karousou, 2011
του Αη Γιωρκού	Solanum nigrum L.	Cyprus (monasteries)	Lardos, 2016
--	--	North Aegean islands	Axiotis, 2018
--	--	Central Macedonia	Tsioutsiou, 2019

Reference details: see separate list

Suggested botanical identity according to the literature

Correspondence between plant name in JC and DMM

Plant name JC (Headword)	Plant name in DMM	Chapter in DMM (Wellmann edition)
τετράγκαθον	τραγάκανθα	III, 20
**Comments**: The name cited in JC corresponds to one of the modern names of the DMM plant τραγάκανθα (see Fraas, 1845: p. 59). This is also supported by information in Gennadios (1914). Fraas (1845: p. 59) claims that the modern name specifically refers to the root of the plant, hence to the herbal drug known as *spina alba*. This is the source of gum tragacanth.		

Information from the literature

a) Botanical texts with reference to the plants in DMM

Plant name in DMM	Chapter in DMM (edition)	Additional names	Plant	Relevant details	Reference
τραγάκανθα	III, 23	Modern: τραγάκανθα, κολλώστουσα	Astragalus aristatus		Billerbeck, 1824
τραγάκανθα	III, 23	Modern: τετράγκαθα, κολλώσκουπα	Astragalus aristatus L'Her.		Fraas, 1845
τραγάκανθα	III, 23	Modern: τετράγκαθα, κολλώσκουπα	Astragalus creticus Sibth.		Fraas, 1845
τραγάκανθα	III, 23	Modern: τετράγκαθα, τραγάκανθα	Astragalus creticus Lam.		Lenz, 1859
τραγακανθα	not mentioned		Astragalus creticus		Sibthorp 1806-40
ποτηριον	not mentioned	Modern: τετράγκαθο	Astragalus aristatus		Sibthorp 1806-40

b) Ethnobotanical field studies

Plant name	Plant	Study region	References
--	--	Cyprus	Arnold-Apostolides, 1985
--	--	Thessaloniki (herbal markets)	Hanlidou, 2004
--	--	Cyprus (wild food plants)	Della, 2006
--	--	Cyprus (herbal markets)	Karousou, 2011
--	--	Cyprus (monasteries)	Lardos, 2016
--	--	North Aegean islands	Axiotis, 2018
--	--	Central Macedonia	Tsioutsiou, 2019

Reference details: see separate list

Suggested botanical identity according to the literature

Correspondence between plant name in JC and DMM

Plant name JC (Headword)	Plant name in DMM	Chapter in DMM (Wellmann edition)
τρίβολος	τρίβολος χερσαίος / τρίβολος ένυδρος	IV, 15 (2 types)
**Comments**: The name cited in JC corresponds to the generic name of the two types of τρίβολος mentioned in the respective DMM chapter. It remains unknown to which type the JC plant refers to.		

Information from the literature

a) Botanical texts with reference to the plants in DMM

Plant name in DMM	Chapter in DMM (edition)	Additional names	Plant	Relevant details	Reference
τρίβολος χερσαίος	IV, 15	Modern: τρίβολι	Tribulus terrestris		Billerbeck, 1824
τρίβολος ένυδρος	IV, 15		Trapa natans		Billerbeck, 1824
τρίβολος χερσαίος	IV, 15	Modern: τριβολάς	Tribulus terrestris L.		Fraas, 1845
τρίβολος χερσαίος	IV, 15	Modern: τριβολάς	Tribulus terrestris L.		Lenz, 1859
τρίβολος ένυδρος	IV, 15		Trapa natans L.		Lenz, 1859
τριβολος χερσαιος	not mentioned		Tribulus terrestris L.		Sibthorp 1806-40
τριβολος ενυδροσς	not mentioned		Trapa natans L.		Sibthorp 1806-40
--	--	Modern: τρίβολι	Onobrychis crista galli		Sibthorp 1806-40

b) Ethnobotanical field studies

Plant name	Plant	Study region	References
trivouli, kollotssidha	Tribulus terrestris L.	Cyprus	Arnold-Apostolides, 1985
--	--	Thessaloniki (herbal markets)	Hanlidou, 2004
--	--	Cyprus (wild food plants)	Della, 2006
--	--	Cyprus (herbal markets)	Karousou, 2011
--	--	Cyprus (monasteries)	Lardos, 2016
τρίβολας	Tribulus terrestris L.	North Aegean islands	Axiotis, 2018
--	--	Central Macedonia	Tsioutsiou, 2019

Reference details: see separate list

Suggested botanical identity according to the literature

Correspondence between plant name in JC and DMM

Plant name JC (Headword)	Plant name in DMM	Chapter in DMM (Wellmann edition)
τριφύλλι	τρίφυλλον	III, 109
**Comments**: The names cited in JC and DMM are consistent.		

Information from the literature

a) Botanical texts with reference to the plants in DMM

Plant name in DMM	Chapter in DMM (edition)	Additional names	Plant	Relevant details	Reference
τρίφυλλον	IV, 171	DMM: λωτός ήμερος;<br>Modern: τριφύλλι	Trifolium messanense		Billerbeck, 1824
τρίφυλλον	III, 123	DMM: οξύφυλλον, μηνυανθές, ασφάλτιον	Psoralea bituminosa		Billerbeck, 1824
λωτός ήμερος	IV, 171	Modern: ήμερον τριφύλλι	Trifolium messanensis L.		Fraas, 1845
τρίφυλλος εν χορτοκοπείοις	II, 176	DMM: λωτός	Trifolium fragiferum L.		Fraas, 1845
--	--	Modern: τριφύλλι	Trifolium spp.	τριφύλλι can apply to various trefoil species	Fraas, 1845
μελίλωτος	III, 41	Modern: τριφύλλι	Melilotus cretica L.		Fraas, 1845
--	--	Modern: τριφύλλι	Melilotus vulgaris L.	rare in Greece	Fraas, 1845
--	--	Modern: τριφύλλι	Melilotus spp.	τριφύλλι can apply to various trefoil species	Fraas, 1845
--	--	Modern: τριφύλλι	Trifolium spp.	τριφύλλι can apply to various trefoil species	Fraas, 1845
λωτός άγριος	IV, 171	Modern: ήμερον τριφύλλι	Trigonella elatior Sibth.		Fraas, 1845
λωτός	III, 113	Modern: άγριο τριφύλλι	Psoralea bituminosa L.		Fraas, 1845
τρίφυλλον	III, 109	DMM: λωτός ήμερος	Trifolium spp.		Lenz, 1859
--		Homer (Il, 2, v.576): λωτός	Trifolium fragiferum L.		Lenz, 1859
λωτος ημερος	not mentioned	Modern: τριφύλλι	Trifolium messanense L.		Sibthorp 1806-40
λωτος ημερος	not mentioned		Melilotus messanensis		Sibthorp 1806-40
λωτος ημερος	not mentioned		Melilotus officinalis		Sibthorp 1806-40

λωτος αγριος	not mentioned		Trigonella elatior		Sibthorp 1806-40
τρίφυλλον	not mentioned		Psoralea bituminosa		Sibthorp 1806-40
--	--	Modern: τριφύλλι	Melilotus spp.		Sibthorp 1806-40
--	--	Modern: τριφύλλι	Trifolium spp.		Sibthorp 1806-40

b) Ethnobotanical field studies

**Plant name**	**Plant**	**Study region**	**References**
--	--	Cyprus	Arnold-Apostolides, 1985
άγριο τριφύλλι	Trifolium pratense L.	Thessaloniki (herbal markets)	Hanlidou, 2004
--	--	Cyprus (wild food plants)	Della, 2006
--	--	Cyprus (herbal markets)	Karousou, 2011
--	--	Cyprus (monasteries)	Lardos, 2016
μελίλοτος	Melilotus indicus (L.) All.	North Aegean islands	Axiotis, 2018
--	--	Central Macedonia	Tsioutsiou, 2019

Reference details: see separate list

Suggested botanical identity according to the literature

Correspondence between plant name in JC and DMM

Plant name JC (Headword)	Plant name in DMM	Chapter in DMM (Wellmann edition)
ψύλλεον	ψύλλιον	IV, 69
**Comments**: The names cited in JC and DMM are consistent.		

Information from the literature

a) Botanical texts with reference to the plants in DMM

Plant name in DMM	Chapter in DMM (edition)	Additional names	Plant	Relevant details	Reference
ψύλλιον	IV, 70		Plantago psyllium		Billerbeck, 1824
ψύλλιον	IV, 70	ψυλλόχορτον	Plantago psyllium L.		Fraas, 1845
ψύλλιον	IV, 70		Plantago arenaria L.		Lenz, 1859
ψύλλιον	IV, 70	ψυλλόχορτον	Plantago psyllium L.		Lenz, 1859
ψυλλιον	Not stated	ψυλλόχορτον	Plantago psyllium L.		Sibthorp 1806-40

b) Ethnobotanical field studies

Plant name	Plant	Study region	References
psillokhorto	Plantago afra L.	Cyprus	Arnold-Apostolides, 1985
--	--	Thessaloniki (herbal markets)	Hanlidou, 2004
--	--	Cyprus (wild food plants)	Della, 2006
--	--	Cyprus (herbal markets)	Karousou, 2011
--	--	Cyprus (monasteries)	Lardos, 2016
--	--	North Aegean islands	Axiotis, 2018
--	--	Central Macedonia	Tsioutsiou, 2019

Reference details: see separate list

Reference list - Reports of suggested botanical identities according to the literature

Botanical and botanical-philological studies on the flora in Ancient Greek and Roman classical texts

Billerbeck, J., 1824. Flora Classica. J.C. Heinrichssche Buchhandlung, Leipzig.

Fraas, C., 1845. Synopsis plantarum florae classicae. E.A. Fleischmann, München.

Lenz, H.O., 1859. Botanik der alten Griechen und Römer. E.F. Thienemann, Gotha.

Sibthorp, J.; Eds. Smith, J.E., Lindley, J., 1806-1840. Flora Graeca, volumes 1-10. Typis Richardi Taylor, Londoni. Online access: Digitale Sammlungen Darmstadt, Technische Univ. Darmstadt: http://tudigit.ulb.tu-darmstadt.de/

Ethnobotanical field studies from Cyprus and Greece

Arnold-Apostolides, N., 1985. *Contribution à la connaissance ethnobotanique et médicinale de la flore de Chypre*. Paris: Université René Descartes.

Axiotis E, Halabalaki M, Skaltsounis LA. An Ethnobotanical Study of Medicinal Plants in the Greek Islands of North Aegean Region. Front Pharmacol. 2018 May 23;9:409. doi: 10.3389/fphar.2018.00409. PMID: 29875656; PMCID: PMC5974156.

Della, A., Hadjichambi, D.P., Hadjichambis, A.Ch., 2006. An ethnobotanical survey of wild edible plants of Paphos and Larnaca countryside of Cyprus. *Journal of Ethnobiology and Ethnomedicine* 2, 34.

Hanlidou E, Karousou R, Kleftoyanni V, Kokkini S. The herbal market of Thessaloniki (N Greece) and its relation to the ethnobotanical tradition. J Ethnopharmacol. 2004 Apr;91(2-3):281-99. doi: 10.1016/j.jep.2004.01.007. PMID: 15120452.

Karousou, R., Deirmentzoglou, S., 2011. The Herbal Market of Cyprus: Traditional Links and Cultural Exchanges. *Journal of Ethnopharmacology* 133, 191–203.

Lardos, A. 2016. Ιατροσοφικά κείμενα και σημερινή χρήση των φυτών σε μοναστήρια στην Κύπρο [Iatrosophia texts and modern use of plant in monasteries on Cyprus]. Λίστα των φυτών που αναφέρθηκαν από τα μοναστήρια κατά την εθνοβοτανική έρευνα [List of the plants reported by the monasteries in the ethnobotanical field study]. Outreach project of the PhD study “Historical iatrosophia texts and modern plant usage in monasteries on Cyprus” conducted at the School of Pharmacy, Univ. London, 2006-2012. Private publication.

Tsioutsiou EE, Giordani P, Hanlidou E, Biagi M, De Feo V, Cornara L. Ethnobotanical Study of Medicinal Plants Used in Central Macedonia, Greece. Evid Based Complement Alternat Med. 2019 Apr 1;2019:4513792. doi: 10.1155/2019/4513792. PMID: 31057648; PMCID: PMC6463668.
