# Supplementary material for: A systematic methodology to assess the identity of plants in historical texts: A case study based on the Byzantine pharmacy text John the Physician’s Therapeutics
Source: J Ethnopharmacol. Author manuscript; Available in PMC 2024 Mar 25. (PMC7615571; doi:10.1016/j.jep.2023.117622)
Supplement: Figure S1 [file EMS193501-supplement-Figure_S1.pptx]

## Slide 1
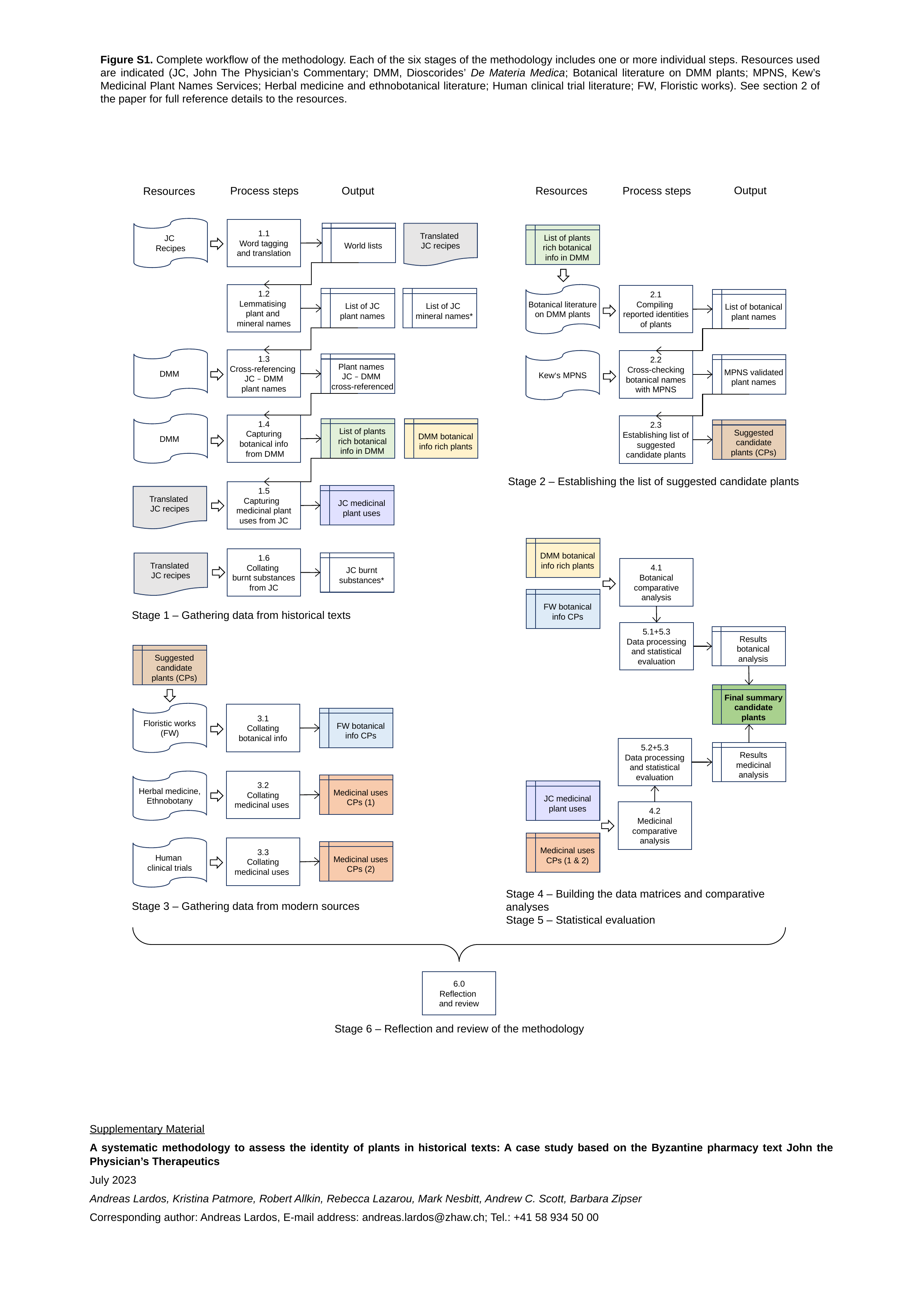

Figure S1. Complete workflow of the methodology. Each of the six stages of the methodology includes one or more individual steps. Resources used are indicated (JC, John The Physician’s Commentary; DMM, Dioscorides’ De Materia Medica; Botanical literature on DMM plants; MPNS, Kew’s Medicinal Plant Names Services; Herbal medicine and ethnobotanical literature; Human clinical trial literature; FW, Floristic works). See section 2 of the paper for full reference details to the resources.
Output
Process steps
Resources
Output
Process steps
Resources
JC
Recipes
1.1
Word tagging
and translation
World lists
Translated
JC recipes
1.2
Lemmatising
plant and
mineral names
List of JC
plant names
List of JC
mineral names*
DMM
1.3
Cross-referencing
JC – DMM
plant names
Plant names
JC – DMM
cross-referenced
DMM
1.4
Capturing
botanical info
 from DMM
List of plants
rich botanical info in DMM
DMM botanical info rich plants
1.5
Capturing
medicinal plant uses from JC
JC medicinal plant uses
Translated
JC recipes
1.6
Collating
burnt substances from JC
JC burnt substances*
Translated
JC recipes
Stage 1 – Gathering data from historical texts
List of plants
rich botanical info in DMM
Botanical literature
on DMM plants
2.1
Compiling
reported identities of plants
List of botanical plant names
Kew‘s MPNS
2.2
Cross-checking botanical names
with MPNS
MPNS validated plant names
2.3
Establishing list of suggested candidate plants
Suggested candidate plants (CPs)
Stage 2 – Establishing the list of suggested candidate plants
DMM botanical info rich plants
4.1
Botanical comparative analysis
FW botanical info CPs
5.1+5.3
Data processing and statistical evaluation
Results botanical analysis
Final summary candidate plants
5.2+5.3
Data processing and statistical evaluation
Results medicinal analysis
JC medicinal plant uses
4.2
Medicinal comparative analysis
Medicinal uses CPs (1 & 2)
Stage 4 – Building the data matrices and comparative analyses
Stage 5 – Statistical evaluation
Suggested candidate plants (CPs)
Floristic works (FW)
3.1
Collating
botanical info
FW botanical info CPs
Herbal medicine, Ethnobotany
3.2
Collating medicinal uses
Medicinal uses CPs (1)
Human
clinical trials
3.3
Collating medicinal uses
Medicinal uses CPs (2)
Stage 3 – Gathering data from modern sources
Stage 6 – Reflection and review of the methodology
6.0
Reflection
and review
Supplementary Material
A systematic methodology to assess the identity of plants in historical texts: A case study based on the Byzantine pharmacy text John the Physician’s Therapeutics
July 2023
Andreas Lardos, Kristina Patmore, Robert Allkin, Rebecca Lazarou, Mark Nesbitt, Andrew C. Scott, Barbara Zipser
Corresponding author: Andreas Lardos, E-mail address: andreas.lardos@zhaw.ch; Tel.: +41 58 934 50 00
