# Supplementary material for: A systematic methodology to assess the identity of plants in historical texts: A case study based on the Byzantine pharmacy text John the Physician’s Therapeutics
Source: J Ethnopharmacol. Author manuscript; Available in PMC 2024 Mar 25. (PMC7615571; doi:10.1016/j.jep.2023.117622)
Supplement: Figure S1 legend [file EMS193501-supplement-Figure_S1_legend.docx]

**Figure S1.** Complete workflow of the methodology. Each of the six stages of the methodology includes one or more individual steps. Resources used are indicated (JC, John The Physician’s Commentary; DMM, Dioscorides’ *De Materia Medica*; Botanical literature on DMM plants; Kew’s MPNS, Kew’s Medicinal Plant Names Services; Herbal medicine and ethnobotanical literature; Human clinical trial literature; FW, Floristic works). See section 2 of the paper for full reference details to the resources.
