# Supplementary material for: A systematic methodology to assess the identity of plants in historical texts: A case study based on the Byzantine pharmacy text John the Physician’s Therapeutics
Source: J Ethnopharmacol. Author manuscript; Available in PMC 2024 Mar 25. (PMC7615571; doi:10.1016/j.jep.2023.117622)
Supplement: Table S1 [file EMS193501-supplement-Table_S1.docx]

**Table S1.** Cross-referencing the 289 plant names in John’s Commentary (JC) with plant names in Dioscorides’ *De Materia Medica* (DMM).

| **Lemma tag** | **JC plant name** | **Category JC plant name** | **#Citat.** | **Connection with DMM** | **References** | **DMM plant name** | **DMM type** | **DMM chapter** | **Category DMM plant name** |
| --- | --- | --- | --- | --- | --- | --- | --- | --- | --- |
| JCLP_002 | ἀγρία | Plant | 8 | Synonym | Sophokles, 1914 | ἀγρώστις | -- | IV, 29 | Plant |
| JCLP_001 | ἀγριαγγουρέα | Plant | 3 | Synonym | Gennadios, 1914 | σίκυς ἄγριος | -- | IV, 150 | Plant |
| JCLP_053 | ἀγριελαία | Plant | 6 | Same name | -- | ἀγριελαία | -- | I, 105 | Plant |
| JCLP_077 | ἀγριοκάρδαμον | Plant | 5 | Unclear | Gennadios, 1914 | λεπίδιον ? | -- | ΙΙ, 174 | Plant |
| JCLP_003 | ἀγριοσταπίδα, ἀγριοσταφίδα | Plant | 19 | Synonym | Sophokles, 1914 | σταφίς ἀγρία | -- | IV, 152 | Plant |
| JCLP_207 | ἀγριοσυκέα | Plant | 3 | Same name | -- | ἄγρια συκή, see σῦκα | -- | See I, 128 | Plant |
| JCLP_004 | ἀδίαντον | Plant | 12 | Same name | -- | ἀδίαντον | -- | IV, 134 | Plant |
| JCLP_005 | ἀθαλή (κενταύριον) | Plant (Synonym) | 3 | Synonym | -- | See κενταύριον | -- | See III, 6-7 | -- |
| JCLP_006 | αἴρα | Plant | 5 | Same name | -- | αἴρα | -- | II, 100 | Plant |
| JCLP_007 | ἀκακία | Plant | 1 | Same name | -- | ἀκακία | -- | I, 101 | Plant |
| JCLP_008 | ἄκανθα, λέγεται τρίβολον | Plant | 2 | Synonym | Linguistic context | See τρίβολος | -- | See IV, 15 | -- |
| JCLP_008 | ἀκάνθη ἄσπρη | Plant | 1 | Synonym | Linguistic context | ἄκανθα λευκή (1) | -- | ΙΙΙ, 12 | Plant |
| JCLP_008 | ἀκάνθη ἄσπρη *ditto* | -- | -- | Synonym | Linguistic context | ἀσπράκανθα (2) | -- | III, 19 | Plant |
| JCLP_009 | ἄκορον | Plant | 2 | Same name | -- | ἄκορον | -- | I, 2 | Plant |
| JCLP_010 | ἀκροαγούρα | Plant | 1 | Synonym | Gennadios, 1914 | See σίκυς ἄγριος | -- | See IV, 150 | -- |
| JCLP_011 | ἀλθαία | Plant | 1 | Same name | -- | ἀλθαία | -- | III, 146 | Plant |
| JCLP_012 | ἀλόη | Plant | 14 | Same name | -- | ἀλόη | -- | III, 22 | Plant |
| JCLP_013 | ἄλυσσος | Plant | 1 | Same name | -- | ἄλυσσον | -- | III, 91 | Plant |
| JCLP_014 | ἀμαράκινον ἐλαίον | Product | 1 | Same name | -- | ἀμαράκινον | -- | I, 58, see ΙΙΙ, 39 | Product |
| JCLP_078 | ἀμάραντον | Plant | 13 | Same name | -- | ἀμάραντον | -- | IV, 57 | Plant |
| JCLP_015 | ἀμμωνιακόν | Plant substance | 27 | Same name | -- | ἀμμωνιακόν | -- | III, 84 | Plant substance |
| JCLP_016 | ἄμπελος | Plant | 10 | Same name | -- | ἄμπελος οἰνόφορος (1) | -- | V, 1 | Plant |
| JCLP_016 | ἄμπελος *ditto* | -- | -- | Same name | -- | ἄμπελος ἄγρια (2) | -- | V, 2; IV, 181 | Plant |
| JCLP_017 | ἀμύγδαλα | Plant | 17 | Same name | -- | ἀμυγδάλη | -- | I, 123 | Plant |
| JCLP_017 | ἀμυγδαλέλαιον | Product | 3 | Same name | -- | ἀμυγδάλινον ἔλαιον | -- | I, 33, see I, 123 | Product |
| JCLP_018 | ἀμώμον | Plant | 3 | Same name | -- | ἄμωμον | -- | I, 15 | Plant substance |
| JCLP_019 | ἀνδράχνη | Plant | 2 | Same name | -- | ἀνδράχνη | -- | II, 124 | Plant |
| JCLP_073 | ἀνηθέλαιον | Product | 8 | Synonym | Linguistic context | ἀνήθου συσκευασία | -- | I, 51, see III, 85 | Product |
| JCLP_073 | ἀνηθίνη, διά | Product | 1 | Source plant | -- | See ἄνηθον | -- | See III, 85 | -- |
| JCLP_073 | ἄνηθον | Plant | 11 | Same name | -- | ἄνηθον | -- | III, 85 | Plant part |
| JCLP_020 | ἄνισον | Plant | 1 | Same name | -- | ἄνησσον | -- | III, 56 | Plant part |
| JCLP_068 | ἀντύβια | Plant | 3 | Synonym | Langkavel, 1866 | See ἰντύβου, in σέρις | -- | See II, 132 | -- |
| JCLP_021 | ἀπίδιν | Plant | 4 | Synonym | Linguistic context | ἄπιον | -- | I, 116 | Plant part |
| JCLP_080 | ἀποβάλσαμον | Plant | 1 | Same name | -- | ὀποβάλσαμον, in βάλσαμον | -- | See I, 19 | Plant substance |
| JCLP_022 | ἀριστολοχία | Plant | 2 | Same name | -- | ἀριστολοχεία | στρογγύλη, θήλεια | III, 4 | Plant |
| JCLP_022 | ἀριστολοχία *ditto* | -- | -- | Same name | Gennadios, 1914 | ἀριστολοχεία | κλιματίτις | III, 4 | Plant |
| JCLP_022 | ἀριστολοχία *ditto* | -- | -- | Same name | -- | ἀριστολοχεία | μακρά, ἄρρην, δακτυλίτις | III, 4 | Plant |
| JCLP_023 | ἀρνόγλωσσον | Plant | 3 | Same name | -- | ἀρνόγλωσσον | μικρόν | II, 126 | Plant |
| JCLP_023 | ἀρνόγλωσσον *ditto* | -- | -- | Same name | -- | ἀρνόγλωσσον | μεῖζον | II, 126 | Plant |
| JCLP_024 | ἄρτος | Product | 7 | Same name | -- | ἄρτος, in πυροί | -- | See II, 85 | Product |
| JCLP_025 | ἄσαρ | Plant | 7 | Same name | -- | ἄσαρον | -- | I, 10 | Plant |
| JCLP_026 | ἀσπαράγγι, ἀσπάραγγος, ἀσπάραγος, σπαράγγι | Plant | 8 | Same name | -- | ἄσπαραγος (πετραῖος ἢ μυάκανθος) | -- | II, 125 | Plant |
| JCLP_027 | ἀσφόδελος | Plant | 18 | Same name | -- | ασφόδελος | -- | II,169 | Plant |
| JCLP_028 | ἀψίνθιον | Plant | 34 | Same name | -- | ἀψίνθιον | -- | III,23 | Plant |
| JCLP_030 | βαλσαμέλαιον, βαρσαμέλαιον | Product | 3 | Source plant | Linguistic context | See βάλσαμον | -- | See I, 19 | -- |
| JCLP_030 | βάλσαμον | Plant | 2 | Same name | -- | βάλσαμον | -- | I, 19 | Plant |
| JCLP_029 | βαμβάκιν, βαμβάκιον | Plant | 2 | No connection | Gennadios, 1914 | -- | -- | -- | -- |
| JCLP_031 | βασιλικόν | Plant | 1 | Synonym | Gennadios, 1914 | ὤκιμον | -- | II, 141 | Plant |
| JCLP_031 | βασιλικόσπορος | Plant part | 2 | Source plant | -- | See ὤκιμον | -- | See II, 141 | -- |
| JCLP_032 | βάτος | Plant | 22 | Same name | -- | βάτος | -- | IV,37 | Plant |
| JCLP_033 | βάτραχος | Plant | 1 | Same name | -- | βατράχιον | βατράχιον | II, 175 | Plant |
| JCLP_033 | βάτραχος *ditto* | -- | -- | Same name | -- | βατράχιον | ἕτερον, σέλινον ἄγριον | II, 175 | Plant |
| JCLP_033 | βάτραχος *ditto* | -- | -- | Same name | -- | βατράχιον | τρίτον | II, 175 | Plant |
| JCLP_033 | βάτραχος *ditto* | -- | -- | Same name | -- | βατράχιον | τέταρτον | II, 175 | Plant |
| JCLP_034 | βδέλλιον | Plant | 2 | Same name | -- | βδέλλιον | -- | I, 67 | Plant substance |
| JCLP_135 | βερονίκη, βερονίκιν | Plant | 4 | No connection | Langkavel, 1866 | -- | -- | -- | -- |
| JCLP_035 | βετονίκη | Plant | 3 | Same name | -- | βεττονίκη, in κέστρον | -- | IV, 1 | Plant |
| JCLP_040 | βλεσκούνι | Plant | 10 | Synonym | Gennadios, 1914 | See γλήχων | -- | See I, 31 | -- |
| JCLP_036 | βολβός | Plant | 4 | Same name | -- | βολβός ἐδώδιμος | -- | II, 170 | Plant part |
| JCLP_037 | βρυωνία | Plant | 1 | Same name | -- | ἄμπελος λευκή, βρυωνία (1) | -- | IV, 182 | Plant |
| JCLP_037 | βρυωνία *ditto* | -- | -- | Same name | -- | ἄμπελος μέλαινα, βρυωνία (2) | -- | IV, 183 | Plant |
| JCLP_038 | βρωμοβότανον (στρύχνον) | Plant (Synonym) | 8 | Synonym | -- | See στρύχνον κηπαίων | -- | See IV, 70 | -- |
| JCLP_039 | γεντιανή | Plant | 2 | Same name | -- | γεντιανή | -- | III, 3 | Plant |
| JCLP_041 | γλιστρίδα, γλυστρίδα (ἀνδράχνη) | Plant (Synonym) | 8 | Synonym | -- | See ἀνδράχνη | -- | See II, 124 | -- |
| JCLP_042 | γλυκοκάλαμον | Plant | 1 | Synonym | Sophokles, 1914 | ἐπί τῶν καλάμων σάκχαρον εὑρισκόμενον, in μέλι | -- | II, 82 | Plant |
| JCLP_043 | γλυκόριζον | Plant | 10 | Same name | -- | γλυκύρριζα | -- | III, 5 | Plant |
| JCLP_040 | γλυχούνη, γληχούνιν | Plant | 8 | Same name | -- | γλήχων | -- | I, 31 | Plant |
| JCLP_044 | γογγύλις | Plant | 2 | Same name | -- | γογγύλη | -- | II, 110 | Plant |
| JCLP_045 | γυμνοκρίθον | Plant | 6 | Synonym | Linguistic context | See κριθή | -- | See II, 86 | -- |
| JCLP_046 | δᾴδιν | Plant part | 3 | No connection | Lidell & Scott, 1883 | -- | -- | -- | -- |
| JCLP_047 | δαμασκηνέα, δαμάσκηνον | Plant | 5 | Synonym | Linguistic context | τῶν ἐν Δαμάσκῳ, in κοκκομηλέα | -- | See I, 121 | Plant |
| JCLP_048 | δαυκίν | Plant | 2 | Synonym | Gennadios, 1914 | σταφύλινος | ἄγριος | III, 52 | Plant |
| JCLP_048 | δαυκίν *ditto* | -- | -- | Same name | -- | δαῦκος | τρίτος | ΙΙΙ, 72 | Plant |
| JCLP_048 | δαυκίν *ditto* | -- | -- | Same name | -- | δαῦκος | ἕτερος | ΙΙΙ, 72 | Plant |
| JCLP_048 | δαυκίν *ditto* | -- | -- | Synonym | Gennadios, 1914 | σταφύλινος | κηπαῖος | III, 52 | Plant |
| JCLP_048 | δαυκίν *ditto* | -- | -- | Same name | -- | δαῦκος | Κρητικός | ΙΙΙ, 72 | Plant |
| JCLP_049 | δαφνέλαιον | Product | 9 | Synonym | Linguistic context | δάφνινον | -- | I, 40, see I, 78 | Product |
| JCLP_049 | δάφνη | Plant | 15 | Same name | -- | δάφνη | -- | I, 78 | Plant |
| JCLP_050 | δενδρολίβανος | Plant | 12 | Synonym | Sophokles, 1914 | λιβανωτίς | -- | III, 75 | Plant |
| JCLP_051 | δρακοντία | Plant | 12 | Same name | -- | δρακοντία μεγάλη | -- | II, 166 | Plant |
| JCLP_051 | δρακοντία *ditto* | -- | -- | Same name |  | δρακοντία μικρά | -- | II, 167 | Plant |
| JCLP_052 | δρῦς | Plant | 1 | Same name | -- | δρῦς | -- | I, 106 | Plant |
| JCLP_053 | ἐλαία | Plant | 19 | Same name | -- | ἐλαία | -- | I, 105 | Plant |
| JCLP_053 | ἐλαία κολυμβάδα | Product | 1 | Source plant | -- | See ἐλαία | -- | See I,105 | -- |
| JCLP_053 | ἔλαιον | Product | 72 | Same name | -- | ἔλαιον | -- | I, 30, see I, 105 | Product |
| JCLP_054 | ἐλελιφασκον | Plant | 2 | Same name | -- | ἐλελίσφακον | -- | III, 33 | Plant |
| JCLP_229 | ἐπίθυμον | Plant | 1 | Same name | -- | ἐπίθυμον | -- | IV, 177 | Plant |
| JCLP_055 | ἐρεβίνθιν | Plant | 11 | Same name | -- | ἐρέβινθος | ἥμερος | II, 104 | Plant |
| JCLP_055 | ἐρεβίνθιν *ditto* | -- | -- | Same name |  | ἐρέβινθος | κριός | II, 104 | Plant |
| JCLP_055 | ἐρεβίνθιν *ditto* | -- | -- | Same name |  | ἐρέβινθος | ἄγριος | II, 104 | Plant |
| JCLP_056 | εὔζωμον | Plant | 2 | Same name | -- | εὔζωμον | -- | II, 140 | Plant |
| JCLP_057 | εὐπατώριος | Plant | 3 | Same name | -- | εὐπατόριος | -- | IV, 41 | Plant |
| JCLP_058 | ἐφόλβιον, εὐφόρβιον | Plant | 5 | Same name | -- | εὐφόρβιον | -- | ΙΙΙ, 82 | Plant |
| JCLP_059 | ζέα | Plant | 1 | Same name | -- | ζέα | -- | II, 89 | Plant |
| JCLP_060 | ζίγγιβερ | Plant | 4 | Same name | -- | ζιγγίβερι | -- | II, 160 | Plant |
| JCLP_062 | ζοχίν | Plant | 3 | Synonym | Linguistic context | σόγχος | ἀγριώτερον | II, 131 | Plant |
| JCLP_062 | ζοχίν *ditto* | -- | -- | Synonym |  | σόγχος | τρυφερώτερον | II, 131 | Plant |
| JCLP_063 | ἡδυόσμος | Plant | 22 | Same name | -- | ἡδύοσμον | -- | III, 34 | Plant |
| JCLP_064 | θέρμια | Plant | 1 | Same name | -- | θέρμος | -- | II, 109 | Plant |
| JCLP_065 | θρύμβος | Plant | 2 | Same name | -- | θύμβρα | -- | III, 37 | Plant |
| JCLP_066 | θύμος, θυμάρι | Plant | 7 | Same name | -- | θύμος | -- | III, 36 | Plant |
| JCLP_068 | ἰντύβιον | Plant | 3 | Same name | -- | ἰντουβου(μ), in σέρις | ἥμερος, ἰντουβου(μ) ἀγρέστε | II, 132 | Plant |
| JCLP_067 | ἰξός τοῦ δρυός | Plant | 2 | Same name | -- | ἰξός | -- | III, 89 | Plant |
| JCLP_069 | ἵππουρις | Plant | 6 | Same name | -- | ἵππουρις | -- | IV, 46 | Plant |
| JCLP_070 | ἶρις | Plant | 1 | Same name | -- | ἶρις | -- | I, 1 | Plant |
| JCLP_071 | ἰσχάδιον, ἰσχάδα | Product | 9 | Synonym | Sophokles, 1914 | ξυρά σῦκα, in σῦκα | -- | See I, 128 | Product |
| JCLP_072 | ἰτέα | Plant | 4 | Same name | -- | ἰτέα | -- | I, 104 | Plant |
| JCLP_072 | ἰτέων, δια | Product | 3 | Source plant | -- | See ἰτέα | -- | See I, 104 | -- |
| JCLP_074 | καλαμίνθη | Plant | 10 | Same name | -- | καλαμίνθη | ὀρινότερα | III, 35 | Plant |
| JCLP_074 | καλαμίνθη *ditto* | -- | -- | Same name |  | καλαμίνθη | ἄγρια, νεπέτα | III, 35 | Plant |
| JCLP_074 | καλαμίνθη *ditto* | -- | -- | Same name |  | καλαμίνθη | τρίτη | III, 35 | Plant |
| JCLP_074 | καλαμίνθη, δια | Product | 2 | Source plant | -- | See καλαμίνθη | -- | See III, 35 | -- |
| JCLP_075 | κάλαμος | Plant | 9 | Same name | -- | κάλαμος | ναστός | I, 85 | Plant |
| JCLP_075 | κάλαμος *ditto* | -- | -- | Same name |  | κάλαμος | θήλυς | I, 85 | Plant |
| JCLP_075 | κάλαμος *ditto* | -- | -- | Same name |  | κάλαμος | συρίγγιας | I, 85 | Plant |
| JCLP_075 | κάλαμος *ditto* | -- | -- | Same name |  | κάλαμος | δόναξ, Κύπριος | I, 85 | Plant |
| JCLP_075 | κάλαμος *ditto* | -- | -- | Same name |  | κάλαμος | φραγμίτης | I, 85 | Plant |
| JCLP_238 | καναβούριν | Plant part | 1 | Synonym | Lidell & Scott, 1883 | καρπόν, in κάνναβις | -- | ΙΙΙ, 148 | Plant part |
| JCLP_239 | κάπνεος, κάπνιος | -- | 2 | Same name | -- | καπνός | -- | IV, 109 | Plant |
| JCLP_076 | κάππαρις | Plant | 15 | Same name | -- | κάππαρις | -- | II,173 | Plant |
| JCLP_077 | καρδάμον | Plant | 7 | Same name | -- | κάρδαμον | -- | II, 155 | Plant |
| JCLP_077 | καρδαμόσπορον | Plant part | 6 | Source plant | -- | See κάρδαμον | -- | See II, 155 | -- |
| JCLP_079 | καρύδι, καρύδιον | Plant part | 11 | Same name | -- | κάρυα βασίλικα | -- | Ι, 125 | Plant part |
| JCLP_081 | καυκαλίδα | Plant | 1 | Same name | -- | καυκαλίς | -- | II, 139 | Plant |
| JCLP_082 | κέγχρος, κεγχρίς | Plant | 4 | Same name | -- | κέγχρος | -- | II, 97 | Plant part |
| JCLP_083 | κέδρεα | Plant substance | 17 | Same name | -- | κεδρία, in κέδρος | -- | I,77 | Plant substance |
| JCLP_085 | κεκήδιν | Plant substance | 20 | Same name | -- | κηκίς | -- | I,107 | Product |
| JCLP_084 | κευταύριον | Plant | 5 | Same name | -- | κευταύριον τὸ μέγα (1) | -- | III, 6 | Plant |
| JCLP_084 | κευταύριον *ditto* | -- | -- | Same name |  | κευταύριον τὸ λεπτόν ἢ μικρόν (2) | -- | III, 7 | Plant |
| JCLP_086 | κινάμωμον, κινάμωνον | Plant part | 7 | Same name | -- | κινάμωμον | -- | I, 14 | Plant part |
| JCLP_087 | κισσός | Plant | 18 | Same name | -- | κισσός | λευκός | II, 179 | Plant |
| JCLP_087 | κισσός *ditto* | -- | -- | Same name |  | κισσός | μέλας | II, 179 | Plant |
| JCLP_087 | κισσός *ditto* | -- | -- | Same name |  | κισσός | ἕλιξ | II, 179 | Plant |
| JCLP_088 | κιτίου, διά | Product | 2 | Same name | -- | κίτριον, Μηδίκά ἢ Περσικά ἢ κεδρόμηλα | -- | I, 115.5 | Plant |
| JCLP_089 | κιχώριον, ἰχώριον | Plant | 5 | Same name | -- | σέρις | ἄγρια, πικρίς, κιχόριον | II, 132 | Plant |
| JCLP_089 | κιχώριον, ἰχώριον *ditto* | -- | -- | Same name |  | σέρις | κηπευτή | II, 132 | Plant |
| JCLP_090 | κλήμαν | Plant | 4 | Synonym | Gennadios, 1914 | See ἄμπελος οἰνόφορος | -- | See V, 1 | -- |
| JCLP_091 | κολίανδρος | Plant | 2 | Synonym | Sophokles, 1914 | κόριον | -- | III, 63 | Plant |
| JCLP_092 | κολοκύνθιν, κολοκίνθη, κολοκύνθιος | Plant | 10 | Same name | -- | κολόκυνθα ἐδώδιμος | -- | II, 134 | Plant |
| JCLP_093 | κολοφώνια | Plant substance | 1 | Same name | -- | κολοφώια, in τέρμινθος | -- | I, 71 | Plant substance |
| JCLP_240 | κομίδιν | Plant substance | 5 | Same name | -- | κόμμι, in κεράσια (1) | -- | I, 113 | Plant substance |
| JCLP_240 | κομίδιν *ditto* | -- | -- | Same name |  | κόμμι, in άμηγδάλη (2) | -- | See I, 123 | Plant substance |
| JCLP_240 | κομίδιν *ditto* | -- | -- | Same name |  | κόμμι, in κοκκυμηλέα (3) | -- | See I, 121 | Plant substance |
| JCLP_094 | κονιδοβότανον (αγριοσταπίδαν) | Plant (Synonym) | 1 | Synonym | -- | See σταφίς ἄγρια | -- | See IV, 152 | -- |
| JCLP_095 | κόνυζα | Plant | 1 | Same name | -- | κόνυζα | μικρά | III, 121 | Plant |
| JCLP_095 | κόνυζα *ditto* | -- | -- | Same name |  | κόνυζα | τρίτον | III, 121 | Plant |
| JCLP_095 | κόνυζα *ditto* | -- | -- | Same name |  | κόνυζα | μείζων | III, 121 | Plant |
| JCLP_096 | κόστος | Plant | 3 | Same name | -- | κόστος | -- | I, 16 | Plant part |
| JCLP_097 | κουκουνάρι | Plant part | 3 | Synonym | Lidell & Scott, 1883 | κώνος τῆς πιτύδος καὶ τῆς πεύκης, see πίτυς, πεύκη | -- | See Ι, 69 | Plant part |
| JCLP_098 | κράμβη | Plant | 17 | Same name | -- | κράμβη | -- | II, 120 | Plant |
| JCLP_099 | κρασίν | Product | 231 | Synonym | Sophokles, 1914 | See οἶνος | -- | See V, 6 | -- |
| JCLP_099 | κρασίν τὸ μυρωδιατόν | Product | 1 | Synonym | Linguistic context | See οἶνος | -- | See V, 6 | -- |
| JCLP_100 | κριθάλευρον | Product | 25 | Source plant | Linguistic context | See κριθή | -- | See II, 86 | -- |
| JCLP_100 | κριθάριν | Plant | 3 | Same name | -- | κριθή | -- | II,86 | Plant |
| JCLP_100 | κρίθινον ἀλεύριν | Product | 2 | Source plant | Linguistic context | See κριθή | -- | See II, 86 | -- |
| JCLP_101 | κρίνον | Plant | 9 | Same name | -- | κρίνον | -- | III, 102 | Plant |
| n/a | κρόκος | Plant part | 11 | Same name | -- | κρόκος | -- | I, 26 | Plant part |
| JCLP_102 | κρομμύδιν, κρόμμυον | Plant | 12 | Same name | -- | κρόμυον | -- | II, 151 | Plant |
| JCLP_103 | κυδωνάτον | Product | 4 | Source plant | Linguistic context | See κυδώνι | -- | See I, 115 | -- |
| JCLP_103 | κυδωνέα | Plant | 2 | Same name | -- | κυδωνία | -- | I, 115 | Plant |
| JCLP_103 | κυδώνι | Plant part | 1 | Same name | -- | κυδώνι, see κυδωνία | -- | See I, 115 | Plant part |
| JCLP_104 | κυκλάμινος | Plant | 17 | Same name | -- | κυκλάμινος | -- | II, 164 | Plant |
| JCLP_105 | κύμινον, κούμινον | Plant | 25 | Same name | -- | κύμινον | -- | III, 59 | Plant |
| JCLP_106 | κυπάρισσος | Plant | 3 | Same name | -- | κυπάρισσος | -- | I, 74 | Plant |
| JCLP_149 | κύπερος, κύπεριν | Plant | 6 | Same name | -- | κύπερος | -- | I, 4 | Plant |
| JCLP_107 | κυπρινέλαιον | Product | 2 | Synonym | Linguistic context | See κύπρινον | -- | See Ι, 55 | -- |
| JCLP_107 | κύπρινον | Product | 1 | Same name | -- | κύπρινον | -- | Ι, 55, see I, 95 | Product |
| JCLP_107 | κύπρος | Plant | 1 | Same name | -- | κύπρος | -- | Ι, 95 | Plant |
| JCLP_108 | κωνάρι | Plant part | 2 | Same name | -- | κῶνος, in πίτυς, πεύκη | -- | See Ι, 69 | Plant part |
| JCLP_109 | κώνειον | Plant | 2 | Same name | -- | κώνειον | -- | IV, 78 | Plant |
| JCLP_110 | λάδανος | Plant substance | 12 | Same name | -- | λάδανος, in κίσθος – λήδον | -- | I, 97.3 | Plant substance |
| JCLP_111 | λάπαθον, λάπατον | Plant | 12 | Same name | -- | λάπαθον | ὀξυλάπαθον | II, 114 | Plant |
| JCLP_111 | λάπαθον, λάπατον *ditto* | -- | -- | Same name |  | λάπαθον | ὀξαλίδα, ἀναξυρίδα, λάπαθον ἄγριον | II, 114 | Plant |
| JCLP_111 | λάπαθον, λάπατον *ditto* | -- | -- | Same name |  | λάπαθον | κηπευτόν | II, 114 | Plant |
| JCLP_111 | λάπαθον, λάπατον *ditto* | -- | -- | Same name |  | λάπαθον | ἄγριον | II, 114 | Plant |
| JCLP_112 | λάχανα | Plant | 5 | No connection | Lidell & Scott, 1883 | -- | -- | -- | -- |
| JCLP_113 | λειχήνη | Plant | 1 | Same name | -- | μυρσίνη ἄγρια, λειχήνη | -- | IV, 144 | Plant |
| JCLP_114 | λίβανος | Plant substance | 29 | Same name | -- | λίβανος | -- | I, 68 | Plant substance |
| JCLP_115 | λιγαία, λιγέα | Plant | 7 | Same name | -- | λύγος, ἄγνος | -- | I, 103 | Plant |
| JCLP_116 | λινόκουκον | Plant part | 2 | Synonym | Linguistic context | See λινόσπερμον | -- | See II,103 | -- |
| JCLP_116 | λινόσπορος | Plant part | 15 | Same name | -- | λινόσπερμον | -- | II,103 | Plant part |
| JCLP_116 | λίνουν πανίον | Product | 1 | No connection | Lidell & Scott, 1883 | -- | -- | -- | -- |
| JCLP_117 | λουλάκιν | Product | 1 | Synonym | Gennadios, 1914 | ἰνδικόν | -- | V, 92 | Product |
| JCLP_118 | λουπηνάρια, λυπηνάρια (θέρμια) | Plant (Synonym) | 21 | Synonym | -- | See θέρμος | -- | See II, 109 | -- |
| JCLP_124 | μαγγούνα | Plant | 1 | Synonym | Langkavel, 1866 | See κώνειον | -- | See IV, 78 | -- |
| JCLP_120 | μακεδονήσιον, μακεδόνιος | Plant | 6 | Synonym | Gennadios, 1914 | See πετροσέλινον | -- | See III, 66 | -- |
| JCLP_121 | μακρόπεπερι | Plant | 1 | Same name | -- | μακρόν πέπερι, in πέπερι | μακρόν πέπερι | II,159 | Plant |
| JCLP_123 | μάλαθρον, μάραθον | Plant | 19 | Same name | -- | μάραθον | -- | III, 70 | Plant |
| JCLP_122 | μανδραγόρας | Plant | 3 | Same name | -- | μανδραγόρας | θήλυς, μέλας, θριδακίας | IV, 75 | Plant |
| JCLP_122 | μανδραγόρας *ditto* | -- | -- | Same name |  | μανδραγόρας | ἄρρην, λευκός, μώριον | IV, 75 | Plant |
| JCLP_119 | μαρούλιον, μαιούλια | Plant | 6 | Synonym | Gennadios, 1914 | θρίδαξ ἥμερος | -- | II, 136 | Plant |
| JCLP_125 | μαστίχη | Plant substance | 18 | Same name | -- | μαστίχη, σχοινίνη, in σχίνος | -- | See I, 70 | Plant substance |
| JCLP_126 | μελάνθιον | Plant | 22 | Same name | -- | μελάνθιον | -- | III,79 | Plant |
| JCLP_127 | μελία | Plant | 1 | Same name | -- | μελία | -- | I, 80 | Plant |
| JCLP_128 | μελίλωτον | Plant | 2 | Same name | -- | μελίλωτος | -- | III, 40 | Plant |
| JCLP_129 | μελισσόφυλλον | Plant | 4 | Same name | -- | μελισσόφυλλον | -- | III, 104 | Plant |
| JCLP_130 | μέσπιλον | Plant | 1 | Same name | -- | μέσπιλον | -- | I, 118 | Plant |
| JCLP_131 | μηλέα | Plant | 1 | Same name | -- | μηλέα | -- | I, 115 | Plant |
| JCLP_131 | μήλον | Plant part | 2 | Same name | -- | μῆλον, in μηλέα | -- | See I, 115 | Plant part |
| JCLP_132 | μολόχιον | Plant | 2 | Same name | -- | μολόχη κηπευτή | -- | II, 118 | Plant |
| JCLP_132 | μολόχιον ἄγριον, ἀγριομολόνχη | Plant | 6 | Synonym | Linguistic context | μολόχη χερσαίας | -- | II, 118 | Plant |
| JCLP_134 | μυρσινέλαιον | Product | 5 | Same name | -- | μυρσινέλαιον | -- | I, 39, see I, 112 | Product |
| JCLP_134 | μυρσίνη | Plant | 14 | Same name | -- | μυρσίνη | -- | I, 112 | Plant |
| JCLP_136 | ναρδέλαιον | Product | 8 | Synonym | Linguistic context | νάρδινον μῦρον | -- | I, 62, see I, 7 | Product |
| JCLP_136 | ναρδοστάχυον | Plant | 1 | Synonym | Gennadios, 1914 | νάρδος | Συριακή | I, 7 | Plant |
| JCLP_136 | ναρδοστάχυον *ditto* | -- | -- | Synonym |  | νάρδος | ᾽Ινδική (Γαγγίτις) | I, 7 | Plant |
| JCLP_136 | ναρδοστάχυον *ditto* | -- | -- | Synonym |  | νάρδος | Σαμφαριτική | I, 7 | Plant |
| JCLP_137 | νάρθηκας, ναρθήκιον | Plant | 2 | Same name | -- | νάρθηξ | -- | III, 77 | Plant |
| JCLP_138 | ξυλαλάς | Plant substance | 1 | Synonym | Gennadios, 1914 | ἀγάλοχον | -- | I, 22 | Plant substance |
| JCLP_140 | ξυλοζιζίβεριν, ξυλοτζιτζίβεριν (ζίγγιβερ) | Plant part (Synonym) | 4 | Synonym | -- | See ζιγγίβερι | -- | See II, 160 | -- |
| JCLP_139 | ξυλοκέρατον | Plant part | 6 | Synonym | Gennadios, 1914 | κεράτια | -- | I, 114 | Plant part |
| JCLP_143 | οἰνάνθη | Plant | 4 | Same name | -- | οἰνάνθη (1) | -- | III, 120 | Plant |
| JCLP_143 | οἰνάνθη *ditto* | -- | -- | Same name |  | οἰνάνθη (2) | -- | V, 4, see V, 1 | Plant part |
| JCLP_144 | οἶνον | Product | 99 | Same name | -- | οἶνος | -- | V, 6, see V, 1 | Product |
| JCLP_142 | ὄξος, ὀξείδιν, ὄξει | Product | 229 | Same name | -- | ὄξος | -- | V, 13, see V, 1 | Product |
| JCLP_145 | ὄπιον | Plant substance | 1 | Same name | -- | ὀπός, in μήκων | κηπευτός, θυλακίτις | ΙV, 64 | Plant substance |
| JCLP_145 | ὄπιον *ditto* | -- | -- | Same name |  | ὀπός, in μήκων | ἄγρια, πιθίτις | ΙV, 64 | Plant substance |
| JCLP_145 | ὄπιον *ditto* | -- | -- | Same name |  | ὀπός, in μήκων | ἀγριωτέρα, μικροτέρα | ΙV, 64 | Plant substance |
| JCLP_141 | ὀρίγανος | Plant | 10 | Same name | -- | ὀρίγανος | -- | III, 27 | Plant |
| JCLP_146 | ὀρόβιν | Plant | 2 | Same name | -- | ὄροβος | -- | II, 108 | Plant |
| JCLP_147 | ὀρύζη | Plant | 2 | Same name | -- | ὄρυζα | -- | II, 95 | Plant |
| JCLP_148 | ὄσπρια | Plant | 6 | No connection | Lidell & Scott, 1883 | -- | -- | -- | -- |
| JCLP_150 | παλιουρέα, παλλιουρέα | Plant | 3 | Same name | -- | παλίουρος | -- | Ι, 92 | Plant |
| JCLP_151 | πανίν | Product | 50 | No connection | Lidell & Scott, 1883 | -- | -- | -- | -- |
| JCLP_152 | πεντάνευρον | Plant | 3 | Synonym | Gennadios, 1914 | See ἀρνόγλωσσον, πολύπλευρον | -- | See ΙΙ, 126 | -- |
| JCLP_154 | πεπέρεων τρίων, διὰ | Product | 3 | Source plant | -- | See πέπερι | -- | See II, 159 | -- |
| JCLP_154 | πέπερι | Plant | 42 | Same name | -- | πέπερι | τέλειον, λευκόν | II,159 | Plant |
| JCLP_153 | πέπων | Plant | 1 | Same name | -- | σίκυς ἥμερος, πέπων | -- | II, 135 | Plant part |
| JCLP_155 | πετροσέλινον | Plant | 2 | Same name | -- | πετροσέλινον | -- | III, 66 | Plant |
| JCLP_156 | πεῦκος | Plant | 1 | Same name | -- | πίτυς, πεύκη | -- | I, 69 | Plant |
| JCLP_157 | πηγανέλαιον | Product | 12 | Source plant | -- | See πήγανον | -- | See III,45 | -- |
| JCLP_157 | πηγανεράς, διὰ | Product | 2 | Source plant | -- | See πήγανον | -- | See III,45 | -- |
| JCLP_157 | πήγανον | Plant | 44 | Same name | -- | πήγανον | ὄρειον | III,45 | Plant |
| JCLP_157 | πήγανος *ditto* | -- | -- | Same name |  | πήγανον | κηπευτόν | III,45 | Plant |
| JCLP_158 | πίσσα | Plant substance | 9 | Same name | -- | πίσσα | -- | I, 72, see I, 69 | Plant substance |
| JCLP_159 | πιστάκια | Plant part | 1 | Same name | -- | πιστάκια | -- | I, 124 | Plant part |
| JCLP_160 | πίτυρον | Product | 25 | Same name | -- | πίτυρον, in πυροί | -- | See II, 85 | Product |
| JCLP_161 | πλάτανος, πλατάνη | Plant | 3 | Same name | -- | πλάτανος | -- | I, 79 | Plant |
| JCLP_162 | πλατυκύμινον | Plant | 1 | Synonym | Langkavel, 1866 | See κύμινον | -- | See III, 59 | -- |
| JCLP_164 | πολύγονον, πολύγονιον | Plant | 4 | Same name | -- | πολύγονον | -- | IV, 4 | Plant |
| JCLP_163 | πολύκομπος, πολλόκομπιν (ἵππουρις) | Plant (Synonym) | 7 | Synonym | -- | See ἵππουρις | -- | See IV, 46 | -- |
| JCLP_165 | πολυπόδιον | Plant | 3 | Same name | -- | πολυπόδιον | -- | IV, 186 | Plant |
| JCLP_166 | πολύτριχον | Plant | 3 | Same name | -- | πολύτριχον, see ἀδίαντον | -- | See IV, 134 | -- |
| JCLP_167 | ποταμογείτανος (καλαμίνθη) | Plant (Synonym) | 15 | Synonym | -- | See καλαμίνθη | -- | See III, 35 | -- |
| JCLP_168 | ποτυρίδα | Plant | 1 | No connection | No reference found | -- | -- | -- | -- |
| JCLP_169 | πράσα, πράσον | Plant | 12 | Same name | -- | πράσον κεφαλωτόν | -- | II, 149 | Plant |
| JCLP_241 | πράσιον | Plant | 1 | Same name | -- | πράσιον | -- | III, 105 | Plant |
| JCLP_241 | πρασίου, διὰ | Product | 1 | Source plant | -- | See πράσιον | -- | See III, 105 | -- |
| JCLP_170 | πρινοκούκιν | Plant substance | 1 | Synonym | Gennadios, 1914 | κόκκος βαφική | -- | IV, 48 | Product |
| JCLP_171 | προυνέα | Plant | 1 | Synonym | Langkavel, 1866 | κοκκυμηλέα | κοκκυμηλέα | I, 121 | Plant |
| JCLP_171 | προυνέα *ditto* | -- | -- | Synonym |  | κοκκυμηλέα | ἄγρία | I, 121 | Plant |
| JCLP_172 | πτελέα | Plant | 4 | Same name | -- | πτελέα | -- | I, 84 | Plant |
| JCLP_173 | πύρεθρος | Plant | 2 | Same name | -- | πύρεθρος | -- | III, 73 | Plant |
| JCLP_174 | ῥάμνος | Plant | 2 | Same name | -- | ῥάμνος | μέλας | I, 90 | Plant |
| JCLP_174 | ῥάμνος *ditto* | -- | -- | Same name |  | ῥάμνος | ἑτέρα | I, 90 | Plant |
| JCLP_174 | ῥάμνος *ditto* | -- | -- | Same name |  | ῥάμνος | τρίτη | I, 90 | Plant |
| JCLP_175 | ῥέον | Plant | 5 | Same name | -- | ῥᾶ, ῥέον | -- | III, 2 | Plant |
| JCLP_176 | ῥεπάνιν, ῥεφάνιν, ῥαφάνιν | Plant | 18 | Same name | -- | ῥαφανίς | -- | II, 112 | Plant |
| JCLP_176 | ῥεπανίου ἐλάδιν, ῥεφανέλαιον | Product | 2 | Synonym | Lidell & Scott, 1883 | ῥαφάνινον | -- | I, 37, see II, 112 | Product |
| JCLP_177 | ῥητίνη, ἐρητίνη | Plant substance | 10 | Same name | -- | ῥητίνη, in τέρμινθος | -- | I, 71 | Plant substance |
| JCLP_178 | ῥόβιν | Plant | 2 | Synonym | Gennadios, 1914 | See ὄροβος | -- | See II, 108 | -- |
| JCLP_181 | ῥόδα | Plant | 14 | Same name | -- | ῥόδον | -- | I,99 | Plant |
| JCLP_179 | ῥοδακινέα | Plant | 1 | Synonym | Gennadios, 1914 | Περσικά μῆλα, in μηλέα | -- | I, 115.4 | Plant part |
| JCLP_179 | ῥοδάκινον | Plant part | 1 | Synonym | Gennadios, 1914 | Περσικά μῆλα, in μηλέα | -- | I, 115.4 | Plant part |
| JCLP_180 | ῥοδάφνη | Plant | 4 | Same name | -- | νέριον, ῥοδοδάφνη | -- | IV, 81 | Plant |
| JCLP_181 | ῥοδέλαιον, | Product | 54 | Synonym | Linguistic context | ῥοδίνου σκευασία | -- | I, 43, see I, 99 | Product |
| JCLP_182 | ῥόδιν, ῥοΐδιν, ῥοΐδιον, ῥοΐδινον | Plant part | 20 | Source plant | Gennadios, 1914 | See ῥόα | -- | See I, 110 | -- |
| JCLP_181 | ῥοδόσταγμα | Product | 9 | No connection | Gennadios, 1914 | -- | -- | -- | -- |
| JCLP_182 | ῥοϊδέα | Plant | 1 | Synonym | Gennadios, 1914 | ῥόα | -- | I, 110 | Plant |
| JCLP_242 | ῥούδιν | Plant | 1 | Synonym | Gennadios, 1914 | ῥοῦς | -- | I, 108 | Plant |
| JCLP_183 | σαγαπήνον | Plant | 1 | Same name | -- | σαγαπήνον | -- | III, 81 | Plant |
| JCLP_184 | σαμψύχον | Plant | 6 | Same name | -- | σαμψούχινον, ἀμάρακος | -- | III, 39 | Plant |
| JCLP_185 | σάχαρ | Product | 6 | Same name | -- | σάκχαρον, in μέλι | -- | II, 82 | Product |
| JCLP_186 | σέλινος, σέληνος | Plant | 18 | Same name | -- | σέλινος | -- | III, 64 | Plant |
| JCLP_186 | σελινόσπορος, σεληνόσπροος | Plant part | 2 | Source plant | -- | See σέλινος | -- | See III, 64 | -- |
| JCLP_187 | σεμίδαλις | Product | 3 | Same name | -- | σεμίδαλις, in πυροί | -- | See II, 85 | Product |
| JCLP_188 | σερφάν (ψύλλεον το λεγομένον) | Plant | 2 | Unclear | -- | σέριφον or ψύλλιον ? | -- | III, 23.5 or IV, 69 | Plant |
| JCLP_189, 236 | σεῦτλον, σεῦκτον | Plant | 11 | Same name | -- | σεῦτλον | -- | II, 123 | Plant |
| JCLP_189 | σευτλόφυλλον | Plant part | 2 | Source plant | -- | See σεῦτλον | -- | See II, 123 | -- |
| JCLP_190 | σησαμέλαιον | Product | 1 | Synonym | Linguistic context | σησάμινον, in βαλάνινον | -- | I, 34, see II, 99 | Product |
| JCLP_190 | σησάμιν | Plant | 3 | Same name | -- | σήσαμον | -- | II, 99 | Plant |
| JCLP_191 | σιδερίτις | Plant | 1 | Same name | -- | σιδερίτις | -- | IV, 33 | Plant |
| JCLP_243 | σίδια (φλούδια ῥοϊδίων) | Plant part | 1 | Source plant | Linguistic context | See ῥόα | -- | See I, 110 | -- |
| JCLP_192 | σινάπιν, συνάπιν | Plant | 9 | Same name | -- | σίνηπι | -- | II, 154 | Plant |
| JCLP_193 | σίτινον ἀλεύριν | Product | 1 | Source plant | Linguistic context | See πυροί, σητάνιος | -- | See II, 85 | -- |
| JCLP_194 | σίτος, σιτάριν | Plant | 6 | Same name | -- | πυροί, σητάνιος | -- | II, 85 | Plant |
| JCLP_196 | σκολοπένδριον, σκολόπενδρα (βοτάνιν τὸ λεγόμενον) | Plant | 4 | Same name | -- | ἄσπληνος, σκολοπένδριον | -- | III, 134 | Plant |
| JCLP_197 | σκόρδον, σκόροδον | Plant | 14 | Same name | -- | σκόρδον | ἥμερον, κηπευτόν | II, 152 | Plant |
| JCLP_197 | σκόρδον, σκόροδον *ditto* | -- | -- | Same name |  | σκόρδον | ὀφιόσκορδον | II, 152 | Plant |
| JCLP_198 | σκορπίουρον | Plant | 2 | Same name | -- | ἡλιοτρόπιον τὸ μέγα, σκορπίουρον | -- | IV, 190 | Plant |
| JCLP_195 | σκυλλοκρέμμυδον, σκυλλοκρόμμυδον | Plant | 8 | Same name | -- | σκίλλα | -- | II, 171 | Plant |
| JCLP_133 | σμύρνα | Plant substance | 16 | Same name | -- | σμύρνα | -- | I, 64 | Plant |
| JCLP_199 | σπαθοβότανον | Plant | 1 | Synonym | Langkavel, 1866 | ξίφιον | -- | IV, 20 | Plant |
| JCLP_200 | σταφίδα, σταπίδα | Plant part | 5 | Same name | -- | σταφίς, in σταφυλή | -- | V, 3, see V, 1 | Plant part |
| JCLP_237 | σταφυλή | Plant part | 1 | Same name | -- | σταφυλή | -- | V, 3, see V, 1 | Plant part |
| JCLP_201 | στάχος | Plant | 5 | Synonym | Langkavel, 1866 | See νάρδος | -- | See I, 7 | -- |
| JCLP_202 | στοιχάς | Plant | 1 | Same name | -- | στοιχάς | -- | III, 26 | Plant |
| JCLP_203 | στρούθιον | Plant | 2 | Same name | -- | στρούθιον | -- | II, 163 | Plant |
| JCLP_204 | στρύχνον | Plant | 4 | Synonym | Langkavel, 1866 | στρύχνον κηπαίων | -- | IV, 70 | Plant |
| JCLP_205 | στυππεῖον | Plant | 7 | No connection | Lidell & Scott, 1883 | -- | -- | -- | -- |
| JCLP_206 | στύρακας, στύραξ | Plant substance | 6 | Same name | -- | στύραξ | -- | I, 66 | Plant substance |
| JCLP_207 | σῦκα | Plant part | 5 | Same name | -- | σῦκα | -- | I, 128 | Plant part |
| JCLP_208 | συκαμινέα | Plant | 7 | Same name | -- | μορέα, συκάμινον | -- | I, 126 | Plant |
| JCLP_207 | συκέα (ἥμερος), συκή | Plant | 4 | Same name | -- | συκῆ (ἥμερος), in σῦκα | -- | I, 128 | Plant |
| JCLP_061 | συρικά ζίντζιφα | Plant | 1 | No connection | Langkavel, 1866 | -- | -- | -- | -- |
| JCLP_209 | σχίνος | Plant | 13 | Same name | -- | σχίνος | -- | I, 70 | Plant |
| JCLP_210 | τετράγκανθον | Plant | 1 | Synonym | Gennadios, 1914 | τραγάκανθα | -- | III, 20 | Plant |
| JCLP_211 | τζουκνίδας | Plant | 6 | Synonym | Gennadios, 1914 | ἀκαλήφη, κνίδη | ἀγριωτέρα, τραχυτέρα | IV, 93 | Plant |
| JCLP_211 | τζουκνίδας *ditto* | -- | -- | Synonym |  | ἀκαλήφη, κνίδη | ἑτέρα, οὐχ ὁμοίως τραχεία | IV, 93 | Plant |
| JCLP_212 | τήλης | Plant | 21 | Same name | -- | τήλις | -- | II,102 | Plant |
| JCLP_213 | τριαντάφυλλον, τριακοντάφυλλον | Plant | 10 | Synonym | Langkavel, 1866 | See ῥόδον | -- | See I, 99 | -- |
| JCLP_214 | τρίβολον | Plant | 1 | Same name | -- | τρίβολος | χερσαίος | IV, 15 | Plant |
| JCLP_214 | τρίβολον *ditto* | -- | -- | Same name |  | τρίβολος | ἔνυδρος | IV, 15 | Plant |
| JCLP_215 | τριφύλλι | Plant | 3 | Same name | -- | τρίφυλλον | -- | III, 109 | Plant |
| JCLP_215 | τριφύλλι *ditto* | -- | -- | Same name | -- | τριφύλλο, in λωτός ἄγριος | -- | IV, 111 | Plant |
| JCLP_215 | τριφύλλι *ditto* | -- | -- | Same name | -- | τρίφυλλον, in λωτός | -- | IV, 110 | Plant |
| JCLP_244 | ὑγροπίσσιν | Plant substance | 13 | Synonym | Linguistic context | πίσσα ἡ μὲν ὑγρά, in πίσσα | -- | I, 72 | Plant substance |
| JCLP_217 | ὑοσκύαμος | Plant | 9 | Same name | -- | ὑοσκύαμος | ἄνθη ὑποπόρφυρα | IV, 68 | Plant |
| JCLP_217 | ὑοσκύαμος *ditto* | -- | -- | Same name |  | ὑοσκύαμος | ἄνθη μηλινοειδή | IV, 68 | Plant |
| JCLP_217 | ὑοσκύαμος *ditto* | -- | -- | Same name |  | ὑοσκύαμος | τρίτος, ἄνθη λευκά | IV, 68 | Plant |
| JCLP_216 | ὕσσωπος | Plant | 20 | Same name | -- | ὕσσωπος | -- | III, 25 | Plant |
| JCLP_218 | φάβα | Plant | 3 | Synonym | Langkavel, 1866 | κύαμος ῾Ελληνικός | -- | II, 105 | Plant |
| JCLP_218 | φαβάτινον ἀλεύριν, φάβατων ἀλεύριν | Product | 2 | Source plant | -- | See κύαμος ῾Ελληνικός | -- | See II, 105 | -- |
| JCLP_219 | φακῆ, φακίν | Plant | 15 | Same name | -- | φακός | -- | II,107 | Plant |
| JCLP_220 | φασούλια | Plant | 1 | No connection | Gennadios, 1914 | -- | -- | -- | -- |
| JCLP_221 | φλόμος | Plant | 2 | Same name | -- | φλόμος | λευκή, θηλεία | IV, 103.1 | Plant |
| JCLP_221 | φλόμος *ditto* | -- | -- | Same name |  | φλόμος | λευκή, ἄρρην | IV, 103.1 | Plant |
| JCLP_221 | φλόμος *ditto* | -- | -- | Same name |  | φλόμος | μέλαινα | IV, 103.2 | Plant |
| JCLP_221 | φλόμος *ditto* | -- | -- | Same name |  | φλόμος | ἄγρία | IV, 103.2 | Plant |
| JCLP_221 | φλόμος *ditto* | -- | -- | Same name |  | φλόμος | φλομίδες δίπλαι 1 | IV, 103.2 | Plant |
| JCLP_221 | φλόμος *ditto* | -- | -- | Same name |  | φλόμος | φλομίδες δίπλαι 2 | IV, 103.2 | Plant |
| JCLP_221 | φλόμος *ditto* | -- | -- | Same name |  | φλόμος | φλομίς, λυχνίτις, θρυαλλίς | IV, 103.2 | Plant |
| JCLP_222 | φοινίκι, φοινίκον, φοινικείον | Plant part | 20 | Same name | -- | φοινικία | -- | I, 109 | Plant |
| JCLP_224 | χαλβάνη, χαλβάνι | Plant | 12 | Same name | -- | χαλβάνι | -- | III, 83 | Plant |
| JCLP_225 | χαμαιδάφνη | Plant | 1 | Same name | -- | χαμαιδάφνη | -- | IV, 147 | Plant |
| JCLP_226 | χαμαίδρυς | Plant | 1 | Same name | -- | χαμαίδρυς | -- | III, 98 | Plant |
| JCLP_227 | χαμαιμηλέλαιον, χαμεμηλέλαιον | Product | 2 | Synonym | Linguistic context | χαμαίμηλον: -ελαίου, in ἀνθεμίς | -- | III, 137 | Product |
| JCLP_227 | χαμαίμιλον, χαμαίμηλα | Plant | 8 | Same name | -- | χαμαίμηλον, in ἀνθεμίς | -- | III, 137 | Plant |
| JCLP_228 | χαμαίπιτυς | Plant | 2 | Same name | -- | χαμαίπιτυς | χαμαίπιτυς | ΙΙΙ, 158 | Plant |
| JCLP_228 | χαμαίπιτυς *ditto* | -- | -- | Same name | -- | χαμαίπιτυς, in ὑπερικόν |  | ΙΙΙ, 154 | Plant |
| JCLP_228 | χαμαίπιτυς *ditto* | -- | -- | Same name |  | χαμαίπιτυς | ἑτέρα | ΙΙΙ, 158 | Plant |
| JCLP_228 | χαμαίπιτυς *ditto* | -- | -- | Same name |  | χαμαίπιτυς | τρίτη | ΙΙΙ, 158 | Plant |
| JCLP_230 | χαρτίν | Product | 1 | Same name | -- | χάρτης, in πάπυρος | -- | I, 86 | Product |
| JCLP_230 | χαρτίν βαμβακερόν | Product | 1 | No connection | Linguistic context | -- | -- | -- | -- |
| JCLP_231 | χελιδονέα, χελιδόνιον | Plant | 4 | Same name | -- | χελιδόνιον μέγα (1) | -- | II, 180 | Plant |
| JCLP_231 | χελιδονέα, χελιδόνιον *ditto* | -- | -- | Same name |  | χελιδόνιον μικρόν (2) | -- | II, 181 | Plant |
| JCLP_232 | χρηστέλαιον | Product | 57 | Synonym | Linguistic context | See ἔλαιον | -- | See I, 30 | -- |
| JCLP_233 | χρυσολάχανον | Plant | 3 | Synonym | Gennadios, 1914 | ἀνδράφαξυς | -- | II, 119 | Plant |
| JCLP_234 | χυλάριν (ζωμίν τοῦ γυμνουκρίθου) | Product | 2 | Source plant | -- | See κριθή | -- | See II, 86 | -- |
| JCLP_235 | ψύλλεον | Plant | 8 | Same name | -- | ψύλλιον | -- | IV, 69 | Plant |

| **Table columns** |  |  |
| --- | --- | --- |
| Lemma tag | Unique identifier of the lemmatised JC plant name. |  |
| JC plant name | Spelling variants of the respective name are also mentioned. |  |
| Category JC plant name | Plant names can refer to the plant as a whole, a plant part, a plant substance (e.g. resins or gums) or a product manufactured of the plant (e.g. oil) |  |
| #Citation | Number of times the respective plant name is mentioned in JC. |  |
| Connection to DMM | Same name – JC plant name is identical with DMM plant name; Source plant – The plant from which the respective plant part, plant substance or products is sourced, is already in the list with a separate entry; Synonym – JC plant name is regarded as a synonym of the respective DMM plant name; No connection – The JC plant name could not be linked with any plant name in DMM. |  |
| References | References used to link JC names with DMM names in case of differing names (see the paper for details to the references) |  |
| DMM plant name | Name in DMM according to Beck (2005) and cross-checked with Wellmann (1907). |  |
| DMM type | Name of “types” of the respective DMM plant as stated in Wellmann (1907). |  |
| DMM chapter | Book volume and chapter in DMM according to Beck (2005). |  |
| Category DMM plant name | Plant names can refer to the plant as a whole, a plant part, a plant substance (e.g. resins or gums) or a product manufactured of the plant (e.g. oil) |  |
|  |  | |
