# Supplementary material for: A systematic methodology to assess the identity of plants in historical texts: A case study based on the Byzantine pharmacy text John the Physician’s Therapeutics
Source: J Ethnopharmacol. Author manuscript; Available in PMC 2024 Mar 25. (PMC7615571; doi:10.1016/j.jep.2023.117622)
Supplement: Table S2 [file EMS193501-supplement-Table_S2.docx]

**Table S2.** Illustration of the botanical comparative analysis at the example of the JC plant name *alyssos* / ἄλυσσος (JCLP013). 1) Descriptions for plants in DMM (Beck, 2005) can be broken down into 36 possible characters (morphological, organoleptic, ecological, geographical). In the case of JCLP013, botanical information for 9 of 36 characters is available in DMM (column A). Three suggested candidate plants (CPs) are reported in the literature: *Fibigia clypeata* (L.) Medik. (CP129), *Odontarrhena alpestris* (L.) Ledeb. (CP130), *Scutellaria galericulata* L. (CP132) (accepted names according to Kew's MPNS). From the Flora of Turkey (FT) (Davis et al., 1965-85), for each CP botanical information referring to the characters mentioned in DMM is extracted (columns B1-3). The information in FT is compared with the corresponding information in DMM and classified (columns C1-3). For the statistical analysis the data in columns C1-3 is codified (columns D1-3).

| **Characters** | **A** | **B1** | **B2** | **B3** | **C1** | **C2** | **C3** | **D1** | **D2** | **D3** |
| --- | --- | --- | --- | --- | --- | --- | --- | --- | --- | --- |
|  | JCLP013 | CP129 | CP130 | CP132 | CP129 | CP130 | CP132 | CP129 | CP130 | CP132 |
| Life form | undershrub (phyriganion) | suffrutescent herb | perennial | perennial herb | partial | partial | partial | 1 | 1 | 1 |
| Habitus general |  |  |  |  |  |  |  | 0 | 0 | 0 |
| Habitus detail 1 | somewhat rough | asperous with stellate hairs | indumentum of stellate hairs |  | good | good | absent | 2 | 2 | 0 |
| Habitus detail 2 |  |  |  |  |  |  |  | 0 | 0 | 0 |
| Height | small | stems usually more than 30 cm | up to 20 cm | 15-50 cm | partial | good | partial | 1 | 2 | 1 |
| Stem general | single stalked |  | with fertile stems | simple or branched | absent | no | partial | 0 | -2 | 1 |
| Stem detail 1 |  |  |  |  |  |  |  | 0 | 0 | 0 |
| Stem detail 2 |  |  |  |  |  |  |  | 0 | 0 | 0 |
| Stem detail 3 |  |  |  |  |  |  |  | 0 | 0 | 0 |
| Leaf general |  |  |  |  |  |  |  | 0 | 0 | 0 |
| Leaf size |  |  |  |  |  |  |  | 0 | 0 | 0 |
| Leaf shape | round | radical leaves oblong, stem leaves linear | obovate-spathulate or oblanceolate | elliptic to lanceolate, cordate, weakly crenate | partial | no | no | 1 | -2 | -2 |
| Leaf edge |  |  |  |  |  |  |  | 0 | 0 | 0 |
| Leaf surface |  |  |  |  |  |  |  | 0 | 0 | 0 |
| Leaf detail 1 |  |  |  |  |  |  |  | 0 | 0 | 0 |
| Leaf detail 2 |  |  |  |  |  |  |  | 0 | 0 | 0 |
| Flower general |  |  |  |  |  |  |  | 0 | 0 | 0 |
| Flower colour |  |  |  |  |  |  |  | 0 | 0 | 0 |
| Flower detail 01 |  |  |  |  |  |  |  | 0 | 0 | 0 |
| Fruit general | adjacent to the leaves |  |  |  | absent | absent | absent | 0 | 0 | 0 |
| Fruit shape | resembling small double shields | flattened siliculae, oblong-elliptic to ovate-oblong, 6 seeds per loculus | siliculae oblong, narrowly to broadly elliptic, inflated, 1 loculate | nutlets depressed-globose to ellipsoid | partial | good | no | 1 | 2 | -2 |
| Fruit structure |  |  |  |  |  |  |  | 0 | 0 | 0 |
| Fruit colour |  |  |  |  |  |  |  | 0 | 0 | 0 |
| Fruit detail 1 |  |  |  |  |  |  |  | 0 | 0 | 0 |
| Seed general | somewhat wide | wing accounting for half or less of the total wide |  | nutlets subglobose or ovoid, commonly tuberculate | partial | absent | no | 1 | 0 | -2 |
| Seed colour |  |  |  |  |  |  |  | 0 | 0 | 0 |
| Seed detail 1 |  |  |  |  |  |  |  | 0 | 0 | 0 |
| Root general |  |  |  |  |  |  |  | 0 | 0 | 0 |
| Root colour |  |  |  |  |  |  |  | 0 | 0 | 0 |
| Root detail 1 |  |  |  |  |  |  |  | 0 | 0 | 0 |
| Root detail 2 |  |  |  |  |  |  |  | 0 | 0 | 0 |
| Smell properties |  |  |  |  |  |  |  | 0 | 0 | 0 |
| Taste properties |  |  |  |  |  |  |  | 0 | 0 | 0 |
| Ethnobotany |  |  |  |  |  |  |  | 0 | 0 | 0 |
| Habitat | mountains,  rough places | rocky slopes, 500-2300m | screes, slopes, forest, 200-3500 m | marshy ground, edges of lakes, rivers, 0-2000m | good | partial | no | 2 | 1 | -2 |
| Distribution |  |  |  |  |  |  |  | 0 | 0 | 0 |

**Table columns**:

A) Description of the DMM plant (book III, chapter 91) corresponding to the JC plant name alyssos/ἄλυσσος with the lemma tag JCLP013;

B) Botanical description in FT for each of the three CPs (CP129: vol.1, p.357; CP130: vol.1, p.401; CP132: vol.7, p.80);

C) Categorisation of the botanical information of each CPs in terms of congruence with the information in DMM (good, partial, no match, absent);

D) Codification of the category of congruence for the statistical analysis (2=good, 1=partial, 0=absent, -2=no match).
