# Supplementary material for: A systematic methodology to assess the identity of plants in historical texts: A case study based on the Byzantine pharmacy text John the Physician’s Therapeutics
Source: J Ethnopharmacol. Author manuscript; Available in PMC 2024 Mar 25. (PMC7615571; doi:10.1016/j.jep.2023.117622)
Supplement: Table S3 [file EMS193501-supplement-Table_S3.docx]

**Table S3.** Statistical evaluation of the botanical comparative analysis (see Table S2) of the example with the JC plant name *alyssos* / ἄλυσσος (JCLP013). Data are shown for the three suggested Candidate Plants (CPs): *Fibigia clypeata* (L.) Medik. (CP129), *Odontarrhena alpestris* (L.) Ledeb. (CP130), *Scutellaria galericulata* L. (CP132).

|  | **A** | **B** | | **C** | **D** |
| --- | --- | --- | --- | --- | --- |
| **CP_ID** | **# Present categories** | **# Congruent categories** | **# Incongruent categories** | **Overall Score** | **Status** |
| CP129 | 7 | 7 | 0 | 7 | STRONG |
| CP130 | 7 | 5 | 2 | 3 | MODERATE |
| CP132 | 7 | 3 | 4 | -1 | WEAK |

**Table columns**: A – Number of available feature categories for each CP; B – Of the categories available (column A), the number demonstrating congruence with JC/DMM and the number demonstrating incongruence; C – Score subtracting incongruent feature categories from congruent feature categories; D – Final candidate strength measure using a scale as follows: Strong (score ≥5), Moderate (score 3-4), Weak (≤2).
